# Systematic revision of the genus *Isodacrys* Sharp, 1911 (Coleoptera: Curculionidae: Entiminae: Tanymecini)

**DOI:** 10.7717/peerj.10191

**Published:** 2020-10-28

**Authors:** Kevin A. Cortés-Hernández, Juan J. Morrone

**Affiliations:** Museo de Zoología “Alfonso L. Herrera”, Departamento de Biología Evolutiva, Facultad de Ciencias, Universidad Nacional Autónoma de México (UNAM), Mexico City, Mexico

**Keywords:** Coleoptera, Curculionidae, Systematics, Phylogenetics, New species

## Abstract

The genus *Isodacrys* Sharp, 1911 is revised. Twenty species of the genus are recognized ranging from south United States of America, Mexico, Guatemala and Honduras of which eight are herein described as new. These species are *Isodacrys antrum* Cortés-Hernández, new species (Mexico: Tamaulipas, Chiapas; Guatemala: Baja Verapaz); *Isodacrys carlae* Cortés-Hernández, new species (Mexico: Coahuila, Hidalgo, Nuevo León, San Luis Potosí, Tamaulipas); *Isodacrys confusum* Cortés-Hernández, new species (Mexico: Tamaulipas); *Isodacrys fasciatum* Cortés-Hernández, new species (Mexico: Coahuila, Durango, Nuevo León); *Isodacrys frontalis* Cortés-Hernández, new species (Mexico: Oaxaca; Guatemala: Sacatepéquez, Guatemala); *Isodacrys kuchii* Cortés-Hernández, new species (Mexico: Puebla); *Isodacrys obrienorum* Cortés-Hernández, new species (Guatemala: Totonicapán, Jalapa, San Marcos); and *Isodacrys okuiltontli* Cortés Hernández, new species (Mexico: Oaxaca). Insights into the monophyly of *Isodacrys* and its phylogenetic relationships with other Tanymecini based on adult morphology are given by implementing a phylogenetic analysis of 43 terminals (21 ingroup, 22 outgroup) coded for 72 adult morphological characters. Characters were discussed and highlighted for the inclusion in the phylogenetic analysis. Final analysis yielded two most-parsimonious cladograms of 242 steps, which support the monophyly of *Isodacrys*. *Isodillex* Cortés-Hernández, new genus is here described to accommodate *Isodillex minutum* (Sharp, 1911), new combination and *Isodillex plumosum* Cortés-Hernández, new species (Mexico: Zacatecas). *Isodillex* was recovered as sister group of *Isodacrys*. Key to separate *Isodacrys* species, occurrence map and habitus photographs are also provided.

## Introduction

Tanymecini (Curculionidae: Entiminae) is a cosmopolitan tribe of broad-nosed weevils with nearly 96 genera, including fossils (Alonso-Zarazaga & Lyal, 1999). The tribe has been traditionally characterized by the presence of modified setae at the anterolateral margin of the prothorax called “postocular vibrissae” (Lacordaire, 1863; LeConte & Horn, 1876; Van Emden, 1944; Howden, 1970), which are long, stout setae projecting towards eyes. They are commonly set on a tuft (as opposed to a fringe) and may be reduced in number or size. In addition to the presence of postocular vibrissae, which is not unique to this weevil tribe, there are additional characters to identify them: eyes in lateral view nearly round; antennal scape usually not reaching anterior margin of prothorax; anterior margin of prothorax in lateral view straight to dorsally produced anteriorly, never forming postocular lobes; mesepimeron not ascending, similar in size to mesepisternum; and metapisternum dilated at its anterior end, with acute process projected inwards between the mesepimeron and the metasternum (Lacordaire, 1863; LeConte & Horn, 1876).

Only the subtribe Tanymecina is represented in the New World (Del Río & Lanteri, 2019b), with 16 genera and approximately 311 described species. The study of the Tanymecini of the Americas has been greatly advanced by the contributions of Anne Howden, who studied them for over 50 years (Anderson & Smith, 2017; Anderson, 2019). During that time, Howden described several genera and numerous species (Howden, 1959, 1961, 1966, 1970, 1982, 2011), highlighting adult characters potentially phylogenetically informative that had not been considered before. Nonetheless, the relationships and boundaries among New World tanymecines remain poorly understood (Howden, 1993a).

Based on Howden’s remarkable contributions, one of the logical and subsequent steps to contribute to the knowledge of the American Tanymecini is the discussion and evaluation of the boundaries among these lineages and discussion of their evolutionary history through phylogenetic analyses (Gillet et al., 2018). As taxonomic concepts, relationships and classifications within Curculionidae are frequently tentative or poorly supported, the choice among mid-level classifications generally remains a matter of author preferences (Oberprieler, 2014). Nevertheless, focusing on a taxonomically manageable lineage such as a genus may yield rather compelling results and contribute to our understanding of weevil evolution (Franz & Engel, 2010). In this context, some recent advances towards understanding the phylogenetic relationships among New World Tanymecini have been made (Jansen & Franz, 2015, 2018). The present study focuses on the delimitation of *Isodacrys* and its phylogenetic relationships with other Tanymecini.

*Isodacrys* Sharp, 1911 is a genus of flightless tanymecines comprised of 13 described species, distributed from southern United States of America to Honduras. Little is known about their natural history: adults have been found on at least nine plant families, feeding on leaves (Burke, 1959) and stems, or in leaf litter and under rocks. *Isodacrys* species have been found in a variety of environments from xerophytic vegetation to pine-oak forests. Because males are unknown in six of the thirteen described species, it has been proposed that they may reproduce by parthenogenesis (Howden, 1961), as it has been confirmed to be the case in other broad-nosed weevils (Lanteri & Normark, 1995). Immature stages remain unknown, but it is assumed that they feed in the soil on roots as other tanymecines do (Howden, 1993a).

The history of the classification of *Isodacrys* is summarized as follows. The genus was described and initially placed within Sciaphilina by Sharp (1911), based on the absence of postocular lobes and its apterous condition, even though it does not have connate tarsal claws as in other Sciaphilina. It included five species: *Isodacrys guatemalenum* Sharp, 1911, *I. minutum* Sharp, 1911, *I. mexicanum* Sharp, 1911, *I. orizabae* Sharp, 1911 and *I. schwarzi* Champion, 1911. Pierce (1913) subsequently designated *I. guatemalenum* as the type species of the genus and transferred *Pandeleteius ovipennis* Schaeffer, 1908 to *Isodacrys*. Apparently based on Sharp’s observations, Pierce included *Isodacrys*, *Pandeleteius* Schönherr, 1834, *Polydacrys* Schönherr, 1834 and *Isodrusus* Sharp, 1911 in a new tribe named Pandeleteini, within the subfamily Tanymecinae (Mitchell & Pierce, 1911), which was characterized by the frequent reduction or absence of postocular vibrissae and separated procoxae. Posteriorly, Van Emden (1944) omitted Pierce’s amendments and included all Tanymecinae within Tanymecini (*sensu* LeConte & Horn, 1876). Howden (1961) reviewed *Isodacrys* and supported its inclusion within Tanymecini, highlighting its affinities with *Pandeleteinus* Champion, 1911, *Isodrusus* and *Minyomerus* Horn, 1876, and posteriorly with the West Indian genera *Paululusus* Howden, 1970 and *Paradacrys* Howden, 1970 (Howden, 1970).

Regardless of efforts to elucidate the boundaries of *Isodacrys* based on adult morphology, many of the character states among putatively related genera overlap or are poorly understood, requiring further discussion of putative homologies that remain obscure. Following a phylogenetic analysis using explicitly defined morphological adult character states, the circumscription of *Isodacrys* is herein reexamined and insights into its generic interrelationships are discussed. Although *Isodacrys* seems to be seldom collected and therefore uncommon in collections, an extensive number of specimens were obtained for study from various museums. As a result, eight *Isodacrys* species new to science are described here. A comprehensive phylogenetic analysis was conducted for 43 terminals (21 outgroups, 22 ingroups) accompanied by a list of the characters included in the analysis and a brief discussion of them, when necessary. From the 72 characters included (53 binary and 19 multistate), 63 comprised external morphology and nine from male and female genitalia. The monophyly of *Isodacrys* is supported by one synapomorphy and two homoplastic characters. In addition, *Isodillex* Cortés-Hernández, new genus is hereby erected to accommodate *Isodillex minutum* (Sharp, 1911), new combination and *Isodillex plumosum* Cortés-Hernández, new species. *Isodillex* is recovered as the sister group to *Isodacrys*.

## Materials and Methods

### Taxon sampling

Tribal concepts follow Alonso-Zarazaga & Lyal (1999), except for the genus *Platyaspistes* Schönherr, 1840 which is considered to belong to Leptopiini Oke, 1951 instead of Tropiphorini Marseul, 1863 (see Marvaldi et al., 2018). An exemplar approach based on morphology was followed (Prendini, 2001). Given that generic relationships among New World Tanymecini remain obscure and that there has only been one specific attempt to estimate the boundaries of one genus (Jansen & Franz, 2015), a comprehensive taxon sampling (Maddison, Donoghue & Maddison, 1984; Nixon & Carpenter, 1993) of putatively related genera (Howden, 1959, 1961, 1963, 1969, 1970) was intended. A total of 43 species was included in order to test the monophyly of *Isodacrys* and elucidate its relationships with other members of the tribe.

The outgroup comprised 21 species representing eight tanymecine genera (Table 1), and one member of the tribes Leptopiini (*Platyaspistes prasinus* [Erichson, 1834], recently transferred from Piazomiina Reitter (1913) by Marvaldi et al. (2018) and Naupactini Gistel (1856) (*Megalostylus albicans* [Lacordaire]), all distributed in the Americas. Specimens of the type species of each outgroup genus were included when available. The ingroup contains 22 species, including the 13 previously described *Isodacrys* species and eight additional herein described. *Isodillex plumosum* new species and *I. minutum* new combination (see results) were also included as part of the ingroup. Specimens of *Isodacrys schwarzi* Champion, 1911 were not available for study, nonetheless, the species was scored and included in the analysis based on habitus photographs of a “cotype” provided by the United States National Museum and Howden’s (1961) redescription. A list of the 43 species included in the analysis is provided (Table 1).

**Table 1:** List of 43 terminal taxa included in the cladistic analysis of *Isodacrys* as well as distributional data. Type species marked with an asterisk.

Taxon	Distribution
Tribe Naupactini Gistel	
*Megalostylus albicans* (Lacordaire)	Mexico
Tribe Leptopiini Oke	
*Platyaspistes prasinus* (Erichson, 1834)*	Chile
Tribe Tanymecini Lacordaire	
*Hadromeropsis brevicoma* Howden, 1982	Mexico
*Minyomerus microps* (Say, 1831)*	Canada, United states of America
*Minyomerus laticeps* (Casey, 1888)	United states of America, Mexico
*Pandeleteius hilaris* (Herbst, 1797)*	Canada, United states of America
*Pandeleteius rotundicollis* (Fall, 1907)	United states of America, Mexico
*Pandeleteius inflatus* Champion, 1911	Mexico
*Scalaventer cyrillae* Howden, 1970*	Jamaica
*Scalaventer jamaicensis* Howden, 1970	Jamaica
*Scalaventer subtropicus* (Fall, 1907)	United states of America, Cuba
*Paululusus hispaniolae* Howden, 1970	Dominican Republic, Haiti
*Paululusus constanzae* Howden, 1970	Dominican Republic
*Isodrusus debilis* Sharp, 1911*	United States of America, Mexico, Guatemala, Honduras
*Isodrusus guajavus*, Howden, 1970	Jamaica
*Aff. Isodrusus* sp.	Colombia
*Pandeleteinus submetallicus* (Schaeffer, 1908)*	United states of America, Mexico
*Pandeleteinus subcancer* Howden, 1969	Mexico
*Pandeleteinus elytroplanatus* Howden, 1959	United States of America, Mexico
*Aff. Pandeleteinus* sp.	Brazil
*Paradacrys ensiformis* Howden, 1970	Bahamas
*Isodillex minutum* (Sharp, 1911) new combination	Mexico
*Isodillex plumosum* new species	Mexico
*Isodacrys antrum* new species	Mexico, Guatemala
*Isodacrys apicale* Howden, 1961	Mexico
*Isodacrys brevirostre* Howden, 1961	Mexico
*Isodacrys buchanani* Howden, 1961	United States of America, Mexico
*Isodacrys burkei* Howden, 1961	United States of America
*Isodacrys carlae* new species	Mexico
*Isodacrys confusum* new species	Mexico
*Isodacrys crispum* Howden, 1961	Mexico
*Isodacrys ellipticum* Howden, 1961	Guatemala, Honduras
*Isodacrys fasciatum* new species	Mexico
*Isodacrys frontalis* new species	Mexico, Guatemala
*Isodacrys geminatum* Howden, 1961	Mexico
*Isodacrys guatemalenum* Sharp, 1911*	Guatemala, Honduras
*Isodacrys kuchii* new species	Mexico
*Isodacrys mexicanum* Sharp, 1911	Mexico
*Isodacrys obrienorum* new species	Guatemala
*Isodacrys okuiltontli* new species	Mexico
*Isodacrys orizabae* Sharp, 1911	Mexico
*Isodacrys ovipennis* (Schaeffer, 1908)	United States of America, Mexico
*Isodacrys schwarzi* Champion, 1911	Mexico

The specimens examined are deposited in the following institutions: MZFC, Museo de Zoología “Alfonso L. Herrera”, Facultad de Ciencias, Universidad Nacional Autónoma de México, Mexico City, Mexico (Juan J. Morrone); IEXA, Colección Entomológica del Instituto de Ecología A.C., Xalapa, Veracruz, Mexico (Leonardo Delgado); CNIN, Colección Nacional de Insectos, Instituto de Biología, Universidad Nacional Autónoma de México, Mexico City, Mexico (Alejandro Zaldívar); UAQE, Colección de Insectos de la Licenciatura en Biología, Facultad de Ciencias Naturales de la Universidad Autónoma de Querétaro, Campus Juriquilla, Querétaro, Mexico (Robert W. Jones); ICZ, Insect Collection of Zacapa, Instituto de Investigaciones del Centro Universitario de Zacapa, Universidad de San Carlos de Guatemala, Zacapa, Guatemala (Manuel A. Barrios-Izás); CMNC, Canadian Museum of Nature Collection, Ottawa, Ontario, Canada (François Génier); TAMUIC, Texas A&M University Insect Collection, Department of Entomology, Texas A&M University, College Station, Texas, United States of America (Karen W. Wright); CAS, California Academy of Sciences, Department of Entomology, San Francisco, California, United States of America (Christopher C. Grinter); ASUCOB, Arizona State University Charles O’Brien Collection, Tempe, Arizona, United States of America (Emmy Engasser); and CNC, Canadian National Collection of Insects, Arachnids and Nematodes, Agriculture and Agri-Food Canada, Ottawa, Canada (Patrice Bouchard).

### Morphological analysis

The analysis was based on 72 discrete characters (53 binary and 19 multistate) of the adults, 63 from external morphology, five from female genitalia and four from male genitalia. Observations and measurements of externally visible and dissected structures were made with a Leica MZ6 stereomicroscope. Habitus photographs were taken using a Leica Z16 APOA stereomicroscope and multilayer images were processed using Leica Application Suite version 4.3.0 software. Photographs of head, ventrites and legs were taken with a Hitachi SU3500 II Scanning Electron Microscope. Genitalia photographs were taken using an AXIO Zoom.V16 stereomicroscope equipped with an AxioCam MRc5 and images were processed using ZEN 2012 software. Illustrations were prepared to exemplify multiple character states, which are highlighted with arrows, with an indication of character numbers and applicable states given in parentheses.

Selection of characters was based on previous literature as noted in the character discussion or based on original observations and comparison of the studied species. Terminology for the external morphology was mainly in accordance with Torre-Bueno (Nichols, 1989) and Howden (1959, 1961, 1969, 1970). Additional specialized terms were used for female (Howden, 1995, 2011; Lanteri & Del Río, 2008) and male genitalia (Bruhn, 1947; Wanat, 2007; Oberprieler, Anderson & Marvaldi, 2014). Specifically, we use the term temones for the apodemes of the median lobe, the term manubrium for the apodeme of tegmen and the term spiculum gastrale for the apodeme of sternite IX (*sensu Oberprieler, Anderson & Marvaldi, 2014)*.

### Phylogenetic analyses

Character statements (i.e., characters and character states) are in accordance with Sereno (2007). An iterative approach was applied to reevaluate and improve homology statements (Hennig, 1966; Franz, 2005). As part of the phylogenetic research cycle, excessively vague and homoplastic characters were reformulated to reflect more accurately synapomorphic conditions in particular lineages (Jenner, 2004; Franz, 2012, 2014). Character states of species that could not be observed were treated as missing data (Maddison, 1993), scored with a “?”. Character states with inapplicable entries on various terminals were scored with a “–”, based on absence statements of the structures under study (Maddison, 1993; Hawkins, Hughes & Scotland, 1997).

The data matrix of 43 terminal taxa and 72 morphological characters (Table 2; Supplemental Information 1) was compiled and edited using WINCLADA version 1.00.08 (Nixon, 2002). Phylogenetic analyses were performed under maximum parsimony as optimality criterion in TNT v1.5 (Goloboff & Catalano, 2016) using heuristic traditional search under equal weights. Cladograms obtained were rooted with *Megalostylus albicans* Champion, 1911 (Entiminae: Naupactini) based on recent analyses of Entiminae weevils depicting the phylogenetic closeness of Naupactini and Tanymecini (Marvaldi et al., 2018; contra Gillet et al., 2018). Most parsimonious trees (MPT’s) were consistently obtained through several rounds of analyses modifying the number of replicates and trees retained per replicate. Final trees were obtained with the following commands: random seed = 1, 300 random addition sequences, swapping algorithm tree bisection and reconnection (TBR), holding 200 trees per replication. From the equally most parsimonious trees, a strict consensus was calculated. The resulting cladograms and character state transformations were examined in WINCLADA under various optimizations. Bremer branch support and parsimony Jackknife values were also calculated in TNT. For Bremer support (Bremer, 1994), 10,000 suboptimal trees up to 15 steps longer than MPT’s were retained. Jackknife values (Farris et al., 1996; Davis, 2011) were computed using 1,000 replications.

**Table 2:** Data matrix of 43 taxa and 72 morphological characters of *Isodacrys* and outgroups, used for the cladistic analysis. Inapplicable and missing character states are indicated with a “–“ and a “?”, respectively. Numbers inside square brackets represent polymorphisms.

Taxon/character	1	2	3	4	5	6	7	8	9	10	11	12	13	14	15	16	17	18
*Megalostylus albicans*	0	0	0	0	0	0	0	–	–	–	0	0	0	0	0	0	–	–
*Platyaspistes prasinus*	0	0	1	0	0	0	0	–	–	–	0	0	0	0	0	0	–	–
*Hadromeropsis brevicoma*	1	0	1	0	0	0	0	–	–	–	1	0	0	1	0	0	–	–
*Minyomerus microps*	2	0	1	0	0	0	1	0	0	0	0	0	1	0	1	0	–	–
*Minyomerus laticeps*	2	0	1	0	0	0	1	0	0	0	0	0	1	0	1	0	–	–
*Pandeleteius hilaris*	0	1	1	2	–	1	1	1	0	0	1	0	1	1	1	0	–	–
*Pandeleteius rotundicollis*	0	1	1	0	0	1	1	0	1	0	1	0	0	1	1	0	–	–
*Pandeleteius inflatus*	0	1	1	2	–	1	1	1	0	0	1	0	0	1	1	0	0	1
*Scalaventer cyrillae*	0	1	2	0	1	1	1	0	1	1	0	0	1	1	2	1	2	1
*Scalaventer jamaicensis*	0	1	2	0	1	1	1	0	1	1	0	0	1	1	2	0	2	1
*Scalaventer subtropicus*	0	1	2	0	1	1	1	0	1	1	0	0	1	1	2	1	2	1
*Paululusus hispaniole*	0	1	2	0	1	1	1	2	1	1	0	0	1	1	2	0	1	0
*Paululusus constanzae*	0	1	2	0	1	1	1	2	1	1	0	0	1	1	2	0	1	0
*Isodrusus debilis*	1	1	1	1	–	1	1	2	0	0	0	0	0	1	4	1	2	2
*Isodrusus guajavus*	0	1	2	1	–	1	1	2	0	0	0	0	1	1	4	1	2	2
*aff. Isodrusus* sp.	0	1	1	0	1	1	1	2	0	0	0	0	1	1	3	1	2	2
*Pandeleteinus submetallicus*	1	1	1	0	0	0	1	2	0	0	1	0	0	1	2	0	2	0
*Pandeleteinus subcancer*	1	1	1	0	0	1	1	2	0	0	0	0	1	1	2	1	2	1
*Pandeleteinus elytroplanatus*	1	1	1	0	0	1	1	2	0	0	1	0	0	1	2	1	2	0
*aff Pandeleteinus* sp.	0	1	1	0	1	1	1	2	0	0	0	0	1	1	3	1	2	2
*Paradacrys ensiformis*	0	1	1	0	1	1	1	2	0	1	0	0	1	1	3	0	2	1
*Isodillex minutum*	0	1	1	0	1	1	1	2	0	0	0	0	0	1	2	1	1	2
*Isodillex plumosum*	0	1	1	0	1	1	1	2	0	0	0	0	0	1	2	1	1	2
*Isodacrys antrum*	1	1	1	0	1	1	1	2	0	0	0	1	0	1	3	1	2	2
*Isodacrys apicale*	1	1	1	0	1	1	1	2	0	0	0	0	1	1	2	1	1	0
*Isodacrys brevirostre*	[01]	1	1	0	1	1	1	2	0	0	0	1	0	1	2	1	1	1
*Isodacrys buchanani*	1	1	1	0	1	1	1	2	0	0	0	0	1	1	2	1	2	1
*Isodacrys burkei*	1	1	1	0	1	1	1	2	0	1	0	0	0	1	2	1	2	0
*Isodacrys carlae*	1	1	1	0	1	1	1	2	0	0	0	0	0	1	2	1	2	0
*Isodacrys confusum*	1	1	1	0	1	1	1	2	0	0	0	0	0	1	2	1	2	0
*Isodacrys crispum*	1	1	1	0	1	1	1	2	0	0	0	0	0	1	3	1	2	1
*Isodacrys ellipticum*	1	1	1	0	1	1	1	2	0	1	0	0	1	1	2	1	1	1
*Isodacrys fasciatum*	1	1	1	0	1	1	1	2	0	0	0	0	0	1	2	1	2	0
*Isodacrys frontalis*	1	1	1	0	1	1	1	2	0	0	0	1	1	1	2	1	1	0
*Isodacrys geminatum*	1	1	1	0	1	1	1	2	0	0	0	1	0	1	3	1	1	2
*Isodacrys guatemalenum*	1	1	1	0	1	1	1	2	0	1	0	0	1	1	2	1	1	1
*Isodacrys kuchii*	1	1	1	0	1	1	1	2	0	0	0	0	1	1	2	1	2	1
*Isodacrys mexicanum*	1	1	1	0	1	1	1	2	0	0	0	0	1	1	2	1	1	0
*Isodacrys obrienorum*	0	1	1	0	1	1	1	2	0	0	0	1	1	1	3	1	2	2
*Isodacrys okuiltontli*	1	1	1	0	1	1	1	2	0	0	0	0	1	1	2	1	1	2
*Isodacrys orizabae*	1	1	1	0	1	1	1	2	0	0	0	0	0	1	2	1	1	0
*Isodacrys ovipennis*	1	1	1	0	1	1	1	2	0	1	0	0	1	1	2	1	1	0
*Isodacrys schwarzi*	1	1	1	?	?	?	1	2	0	0	0	0	?	1	2	1	2	0

Taxon/character	19	20	21	22	23	24	25	26	27	28	29	30	31	32	33	34	35	36
*Megalostylus albicans*	0	0	0	0	0	0	0	0	0	0	0	0	0	0	–	–	0	–
*Platyaspistes prasinus*	0	1	1	0	1	0	0	0	0	0	0	0	0	–	–	–	0	–
*Hadromeropsis brevicoma*	1	1	0	0	1	1	–	1	1	1	0	1	0	1	0	0	0	0
*Minyomerus microps*	1	0	0	0	0	0	0	1	2	2	0	1	1	1	1	2	0	0
*Minyomerus laticeps*	1	0	0	0	0	0	0	1	2	2	0	1	1	1	0	0	0	0
*Pandeleteius hilaris*	1	1	0	1	1	1	–	1	2	1	1	1	1	1	0	0	1	0
*Pandeleteius rotundicollis*	1	1	0	1	1	1	–	1	2	1	0	1	1	1	0	0	1	0
*Pandeleteius inflatus*	1	1	0	1	1	1	–	1	2	1	1	1	1	1	0	0	1	0
*Scalaventer cyrillae*	1	1	0	1	1	1	–	1	2	2	1	1	1	0	–	–	1	1
*Scalaventer jamaicensis*	1	0	0	1	1	1	–	1	2	2	1	1	1	0	–	–	1	0
*Scalaventer subtropicus*	1	0	0	1	1	1	–	1	2	2	1	1	1	0	–	–	1	1
*Paululusus hispaniole*	1	0	0	1	1	1	–	1	2	2	1	1	1	0	–	–	1	0
*Paululusus constanzae*	1	0	0	1	1	1	–	1	2	2	1	1	1	0	–	–	1	0
*Isodrusus debilis*	1	0	0	1	1	1	–	1	2	2	2	1	1	1	1	1	1	0
*Isodrusus guajavus*	1	0	0	1	1	1	–	1	2	2	2	1	1	0	–	–	1	0
*aff. Isodrusus* sp.	1	0	0	1	1	1	–	1	2	2	1	1	1	[01]	1	2	1	0
*Pandeleteinus submetallicus*	1	1	0	0	1	1	–	1	2	1	1	1	1	1	1	1	1	0
*Pandeleteinus subcancer*	1	0	0	1	1	1	–	1	2	2	2	1	1	1	1	1	1	0
*Pandeleteinus elytroplanatus*	1	1	0	1	1	1	–	1	2	2	1	1	1	1	0	0	1	0
*aff Pandeleteinus* sp.	1	0	0	1	1	1	–	1	2	2	1	1	1	1	1	1	1	0
*Paradacrys ensiformis*	1	0	0	1	1	1	–	1	2	2	1	1	1	0	–	–	1	0
*Isodillex minutum*	1	1	0	1	1	1	–	1	2	2	1	1	1	0	–	–	1	0
*Isodillex plumosum*	1	1	0	1	1	1	–	1	2	2	1	1	1	0	–	–	1	0
*Isodacrys antrum*	1	1	0	1	1	1	–	1	2	2	1	1	1	0	–	–	1	0
*Isodacrys apicale*	1	1	0	1	1	1	–	1	2	2	1	1	1	1	0	1	1	0
*Isodacrys brevirostre*	1	1	0	1	1	1	–	1	2	2	2	1	1	0	–	–	1	0
*Isodacrys buchanani*	1	0	0	1	1	0	1	1	2	2	2	1	1	1	1	2	1	0
*Isodacrys burkei*	1	1	1	1	1	0	1	1	2	2	2	1	1	0	–	–	1	0
*Isodacrys carlae*	1	0	0	1	1	1	–	1	2	2	2	1	1	0	–	–	1	0
*Isodacrys confusum*	1	0	0	1	1	0	1	1	2	2	1	1	1	0	–	–	1	0
*Isodacrys crispum*	1	0	0	1	1	1	–	1	2	2	2	1	1	1	1	2	1	0
*Isodacrys ellipticum*	1	0	0	1	1	1	–	1	2	2	2	1	1	0	–	–	1	0
*Isodacrys fasciatum*	1	0	0	1	1	1	–	1	2	2	1	1	1	0	–	–	1	0
*Isodacrys frontalis*	1	1	0	1	1	1	–	1	2	2	1	1	1	0	–	–	1	0
*Isodacrys geminatum*	1	1	0	1	1	1	–	1	2	2	2	1	1	0	–	–	1	0
*Isodacrys guatemalenum*	1	0	0	1	1	1	–	1	2	2	2	1	1	0	–	–	1	0
*Isodacrys kuchii*	1	1	0	1	1	1	–	1	2	2	2	1	1	0	–	–	1	0
*Isodacrys mexicanum*	1	0	0	1	1	1	–	1	2	2	2	1	1	1	1	2	1	0
*Isodacrys obrienorum*	1	1	0	1	1	1	–	1	2	2	1	1	1	[01]	1	2	1	0
*Isodacrys okuiltontli*	1	0	0	1	1	1	–	1	2	2	2	1	1	0	–	–	1	0
*Isodacrys orizabae*	1	1	1	1	1	1	–	1	2	2	2	1	1	[01]	1	2	1	0
*Isodacrys ovipennis*	1	0	1	1	1	0	1	1	2	2	2	1	1	1	1	1	1	0
*Isodacrys schwarzi*	1	?	0	1	?	0	1	1	2	2	1	1	1	1	0	?	1	0

Taxon/character	37	38	39	40	41	42	43	44	45	46	47	48	49	50	51	51	53	54
*Megalostylus albicans*	0	0	0	–	0	–	0	0	0	0	0	0	0	0	0	0	0	0
*Platyaspistes prasinus*	0	0	0	–	0	–	0	0	0	0	0	0	–	0	0	0	0	1
*Hadromeropsis brevicoma*	0	1	0	–	2	0	0	0	0	0	0	0	0	0	2	1	1	1
*Minyomerus microps*	0	2	1	1	1	–	1	–	0	0	0	0	0	0	0	0	0	0
*Minyomerus laticeps*	0	2	1	1	1	–	1	–	0	0	0	0	0	0	0	0	0	0
*Pandeleteius hilaris*	0	1	0	–	2	0	0	0	1	0	1	0	0	0	1	1	1	1
*Pandeleteius rotundicollis*	0	1	0	–	2	0	0	0	0	0	1	0	0	0	2	1	1	1
*Pandeleteius inflatus*	0	3	0	–	2	1	0	0	0	0	0	0	–	0	1	1	1	1
*Scalaventer cyrillae*	0	1	0	–	2	0	0	1	1	0	1	0	0	0	1	1	1	1
*Scalaventer jamaicensis*	0	1	0	–	2	0	0	1	0	0	1	0	0	0	1	1	1	1
*Scalaventer subtropicus*	0	1	0	–	2	0	0	1	1	0	1	0	0	0	1	1	1	1
*Paululusus hispaniole*	0	1	0	–	2	0	0	1	0	0	0	0	1	1	2	1	1	1
*Paululusus constanzae*	1	3	1	0	2	1	0	1	0	0	0	0	0	1	2	1	1	1
*Isodrusus debilis*	0	1	0	–	2	0	0	1	0	0	0	0	0	0	1	0	0	0
*Isodrusus guajavus*	0	1	0	–	2	0	0	1	0	0	0	0	0	0	1	1	1	1
*aff. Isodrusus* sp.	0	3	1	1	2	1	0	0	0	0	0	0	–	0	1	1	1	1
*Pandeleteinus submetallicus*	0	1	0	–	2	0	0	1	0	0	0	0	0	0	1	1	1	1
*Pandeleteinus subcancer*	0	1	0	–	2	0	0	1	0	0	0	0	0	0	1	1	1	0
*Pandeleteinus elytroplanatus*	0	1	0	–	2	0	0	1	0	0	0	0	0	0	1	1	0	1
*aff Pandeleteinus* sp.	0	1	0	–	2	0	0	0	0	0	0	0	0	0	1	1	1	1
*Paradacrys ensiformis*	0	1	0	–	2	0	0	0	0	0	0	0	0	0	1	1	1	1
*Isodillex minutum*	0	3	1	0	2	1	0	1	0	0	0	0	–	0	1	1	1	1
*Isodillex plumosum*	0	3	1	0	2	1	0	1	0	0	0	0	0	0	1	1	1	1
*Isodacrys antrum*	0	3	1	1	2	1	0	1	0	0	0	1	0	0	1	0	0	1
*Isodacrys apicale*	0	2	1	0	2	1	0	1	0	0	0	0	0	1	1	0	0	1
*Isodacrys brevirostre*	0	3	1	1	2	1	0	1	0	0	0	1	0	0	1	0	0	0
*Isodacrys buchanani*	0	3	1	0	2	2	0	1	0	0	0	0	1	[01]	1	0	0	0
*Isodacrys burkei*	0	4	1	0	2	1	0	1	0	1	1	0	0	0	1	1	1	1
*Isodacrys carlae*	0	2	1	1	2	1	0	1	0	0	0	0	0	0	1	1	0	0
*Isodacrys confusum*	0	2	1	1	2	1	0	1	0	0	0	0	0	0	1	1	0	0
*Isodacrys crispum*	0	3	1	0	2	1	0	1	0	0	0	0	0	0	1	0	0	1
*Isodacrys ellipticum*	1	3	1	0	2	1	0	1	0	0	0	0	0	0	1	1	1	1
*Isodacrys fasciatum*	0	2	1	1	2	1	0	1	0	0	0	0	0	0	1	1	0	0
*Isodacrys frontalis*	0	3	1	1	2	1	0	1	0	0	0	1	0	0	1	0	0	0
*Isodacrys geminatum*	0	2	1	1	2	1	0	1	0	0	0	0	0	0	1	0	0	0
*Isodacrys guatemalenum*	1	3	1	0	2	1	0	1	0	0	0	0	0	0	1	1	1	1
*Isodacrys kuchii*	0	2	1	1	2	1	0	1	0	0	0	0	0	0	1	1	0	0
*Isodacrys mexicanum*	0	2	1	0	2	1	0	1	0	0	1	0	0	0	1	1	1	1
*Isodacrys obrienorum*	0	2	1	1	2	1	0	1	0	0	0	0	0	0	1	0	0	0
*Isodacrys okuiltontli*	0	2	1	0	2	1	0	1	0	0	0	0	0	0	1	0	0	1
*Isodacrys orizabae*	0	[23]	1	0	2	1	0	1	0	0	0	0	0	0	1	1	1	1
*Isodacrys ovipennis*	0	4	1	0	2	1	0	1	0	1	1	0	0	0	1	1	1	1
*Isodacrys schwarzi*	0	3	1	0	2	2	0	1	0	0	0	0	1	?	1	0	0	0

Taxon/character	55	56	57	58	59	60	61	62	63	64	65	66	67	68	69	70	71	72
*Megalostylus albicans*	0	0	–	0	0	0	0	–	–	0	–	0	0	0	0	0	0	0
*Platyaspistes prasinus*	0	0	–	1	0	1	0	–	–	?	?	?	?	?	?	?	?	?
*Hadromeropsis brevicoma*	0	1	0	2	0	0	0	–	–	2	1	?	2	0	0	0	0	0
*Minyomerus microps*	1	0	–	0	1	0	0	–	–	1	–	0	2	0	–	–	–	–
*Minyomerus laticeps*	1	0	–	0	1	0	0	–	–	1	–	0	2	0	?	?	?	?
*Pandeleteius hilaris*	0	1	0	2	0	0	0	–	–	2	0	1	1	1	1	0	0	0
*Pandeleteius rotundicollis*	0	1	0	2	0	0	0	–	–	2	0	1	1	1	0	1	0	0
*Pandeleteius inflatus*	0	1	0	2	0	0	0	–	–	2	0	1	1	1	?	?	?	?
*Scalaventer cyrillae*	0	1	1	2	0	0	1	1	0	2	1	1	1	1	0	1	0	0
*Scalaventer jamaicensis*	0	1	1	2	0	0	1	1	0	2	1	1	1	1	0	1	0	0
*Scalaventer subtropicus*	0	1	1	2	0	0	1	1	0	2	1	1	1	1	1	1	0	0
*Paululusus hispaniole*	0	1	0	2	0	0	1	0	1	2	1	1	1	1	1	1	0	0
*Paululusus constanzae*	0	1	0	2	0	0	1	0	1	2	1	1	1	1	1	1	0	?
*Isodrusus debilis*	0	1	0	2	0	1	1	1	0	2	0	1	1	1	–	–	–	–
*Isodrusus guajavus*	0	1	0	2	0	1	1	1	0	?	?	?	?	?	?	?	?	?
*aff. Isodrusus* sp.	0	1	0	2	0	1	1	1	0	?	?	?	?	?	1	?	0	?
*Pandeleteinus submetallicus*	0	1	0	2	0	0	1	0	1	2	0	1	1	1	1	1	0	2
*Pandeleteinus subcancer*	0	1	0	2	0	0	1	0	0	2	0	1	1	1	1	1	0	2
*Pandeleteinus elytroplanatus*	0	1	0	2	0	0	1	0	1	2	0	1	1	1	1	1	0	2
*aff Pandeleteinus* sp.	0	1	0	2	0	0	1	0	1	2	0	1	1	1	1	1	1	0
*Paradacrys ensiformis*	0	1	0	2	0	0	1	0	1	2	0	1	1	1	0	1	1	0
*Isodillex minutum*	0	1	0	2	0	0	0	–	–	2	1	1	1	1	–	–	–	–
*Isodillex plumosum*	0	1	0	2	0	0	0	–	–	2	1	1	1	1	1	1	0	0
*Isodacrys antrum*	0	1	0	2	0	0	1	0	1	–	–	1	1	1	–	–	–	–
*Isodacrys apicale*	0	1	0	2	0	0	1	0	1	?	?	?	?	?	?	?	?	?
*Isodacrys brevirostre*	0	1	0	2	0	0	1	0	1	2	0	1	1	1	–	–	–	–
*Isodacrys buchanani*	0	1	0	2	0	0	1	0	1	2	0	1	1	1	1	1	0	1
*Isodacrys burkei*	0	1	0	2	0	0	1	0	1	2	0	1	1	1	1	0	1	1
*Isodacrys carlae*	0	1	0	2	0	0	1	0	1	2	0	1	1	1	–	–	–	–
*Isodacrys confusum*	0	1	0	2	0	0	1	0	1	2	0	1	1	1	1	1	0	2
*Isodacrys crispum*	0	1	0	2	0	0	1	0	0	2	0	1	1	1	–	–	–	–
*Isodacrys ellipticum*	0	1	0	2	0	0	1	0	1	2	0	1	1	1	–	–	–	–
*Isodacrys fasciatum*	0	1	0	2	0	0	1	0	1	2	0	1	1	1	–	–	–	–
*Isodacrys frontalis*	0	1	0	2	0	0	1	0	1	2	0	1	1	1	–	–	–	–
*Isodacrys geminatum*	0	1	0	2	0	0	1	0	1	2	1	1	1	1	–	–	–	–
*Isodacrys guatemalenum*	0	1	0	2	0	0	1	0	1	2	1	1	1	1	1	1	1	1
*Isodacrys kuchii*	0	1	0	2	0	0	1	0	1	2	0	1	1	1	1	1	1	2
*Isodacrys mexicanum*	0	1	0	2	0	0	1	0	1	2	1	1	1	1	1	0	0	1
*Isodacrys obrienorum*	0	1	0	2	0	0	1	0	1	2	1	1	1	1	1	1	0	2
*Isodacrys okuiltontli*	0	1	0	2	0	0	1	0	1	2	1	1	1	1	1	1	1	?
*Isodacrys orizabae*	0	1	0	2	0	0	1	0	1	2	0	1	1	1	1	0	1	0
*Isodacrys ovipennis*	0	1	0	2	0	0	1	0	1	2	0	1	1	1	1	0	1	1
*Isodacrys schwarzi*	0	1	0	2	0	0	1	0	1	?	?	?	?	?	?	?	?	?

### Taxa description and nomenclature

The genus-level diagnoses of *Isodacrys* and *Isodillex* new genus highlight characters present in all their members and accounts for their variability. Species-level diagnoses, although similarly structured, represent unique complementary accounts of character states observed in each species. For ease of comparison, characters given in descriptions of new taxa follow the sequence of characters included in the phylogenetic analysis. Additional characters not included in the data matrix are displayed accordingly with the body part sequence that is presented as follows: Size, dorsal coverture of integument, rostrum, head, prothorax, elytra, legs, abdomen and genitalia. Body length and width were measured in dorsal view from the frons to the apex of the elytra and at the widest point of the elytra, respectively. An identification key to the species of *Isodacrys* is arranged with emphasis being placed on the most readily observable characters.

The electronic version of this article in Portable Document Format (PDF) will represent a published work according to the International Commission on Zoological Nomenclature (ICZN), and hence the new names contained in the electronic version are effectively published under that Code from the electronic edition alone. This published work and the nomenclatural acts it contains have been registered in ZooBank, the online registration system for the ICZN. The ZooBank LSIDs (Life Science Identifiers) can be resolved and the associated information viewed through any standard web browser by appending the LSID to the prefix http://zoobank.org/. The LSID for this publication is: [urn:lsid:zoobank.org:pub:F5D5C6F7-3FB4-4D1F-91F3-0D6380C8FF59]. The online version of this work is archived and available from the following digital repositories: PeerJ, PubMed Central and CLOCKSS.

Isodacrys antrum: Isodacrys carlae: Isodacrys confusum; Isodacrys fasciatum: Isodacrys frontalis: Isodacrys kuchii: Isodacrys obrienorum: Isodacrys okuiltontli: Isodillex: Isodillex plumosum:

### Distribution of *Isodacrys* species

For the material examined for each species of *Isodacrys*, all localities and sampling points were georeferenced and included in the occurrence maps done in QGIS Version 3.12.3-București (Quantum GIS Development Team, 2020).

## Results

### Phylogenetic analysis

**Figure 1:**
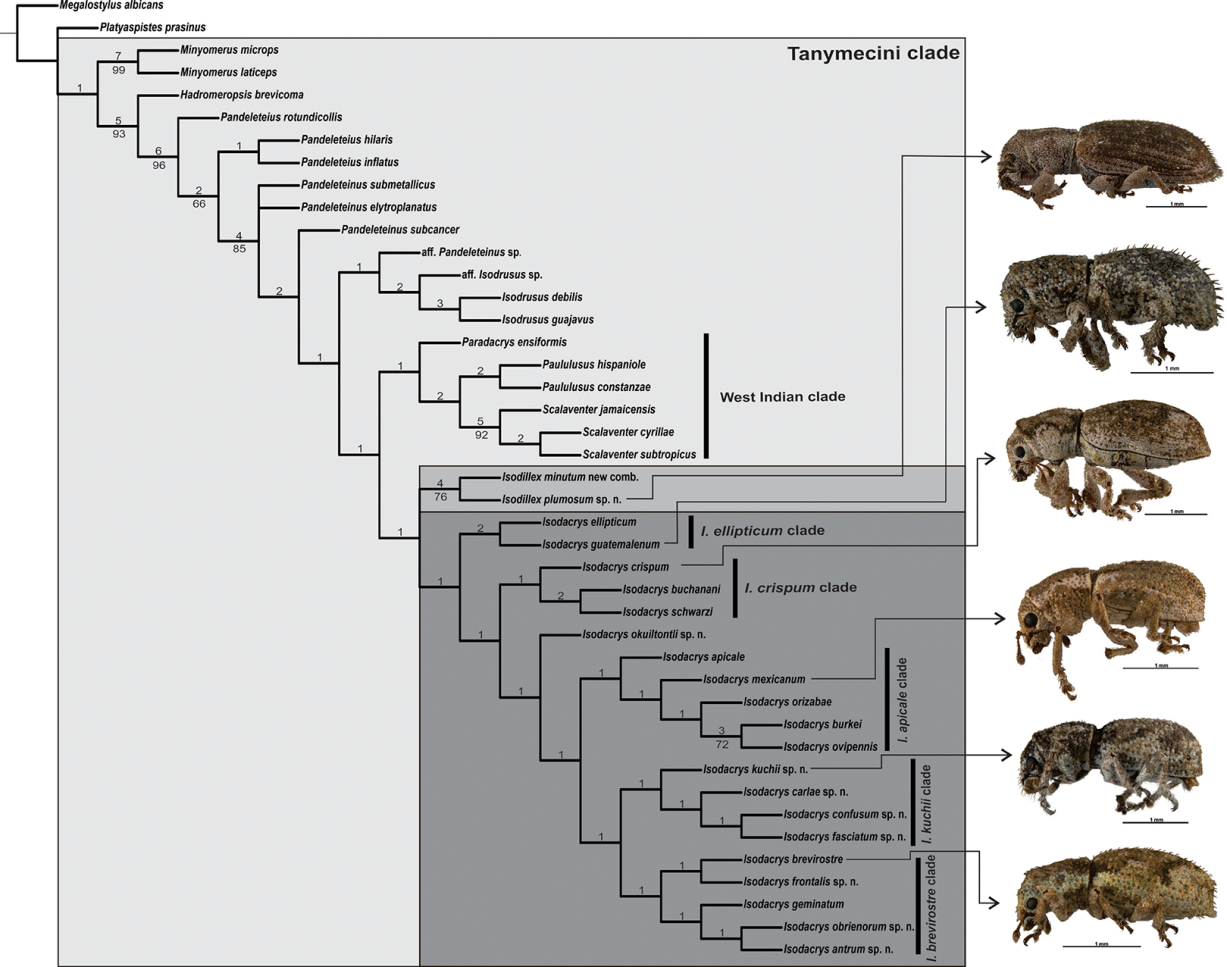
Strict consensus of two most parsimonious trees obtained from the analysis under equal weights (*L* = 244, CI = 0.38, RI = 0.69). Node regarding the position of *Pandeleteinus submetallicus* and *P. elytroplanatus* is collapsed. Numbers above and below branches indicate Bremer support and Jackknife values (cut-off at 63%), respectively. A lateral habitus photograph of one species from each clade within *Isodacrys* is provided as it is for *Isodillex*, its sister taxon (see “Discussion”).

**Figure 2:**
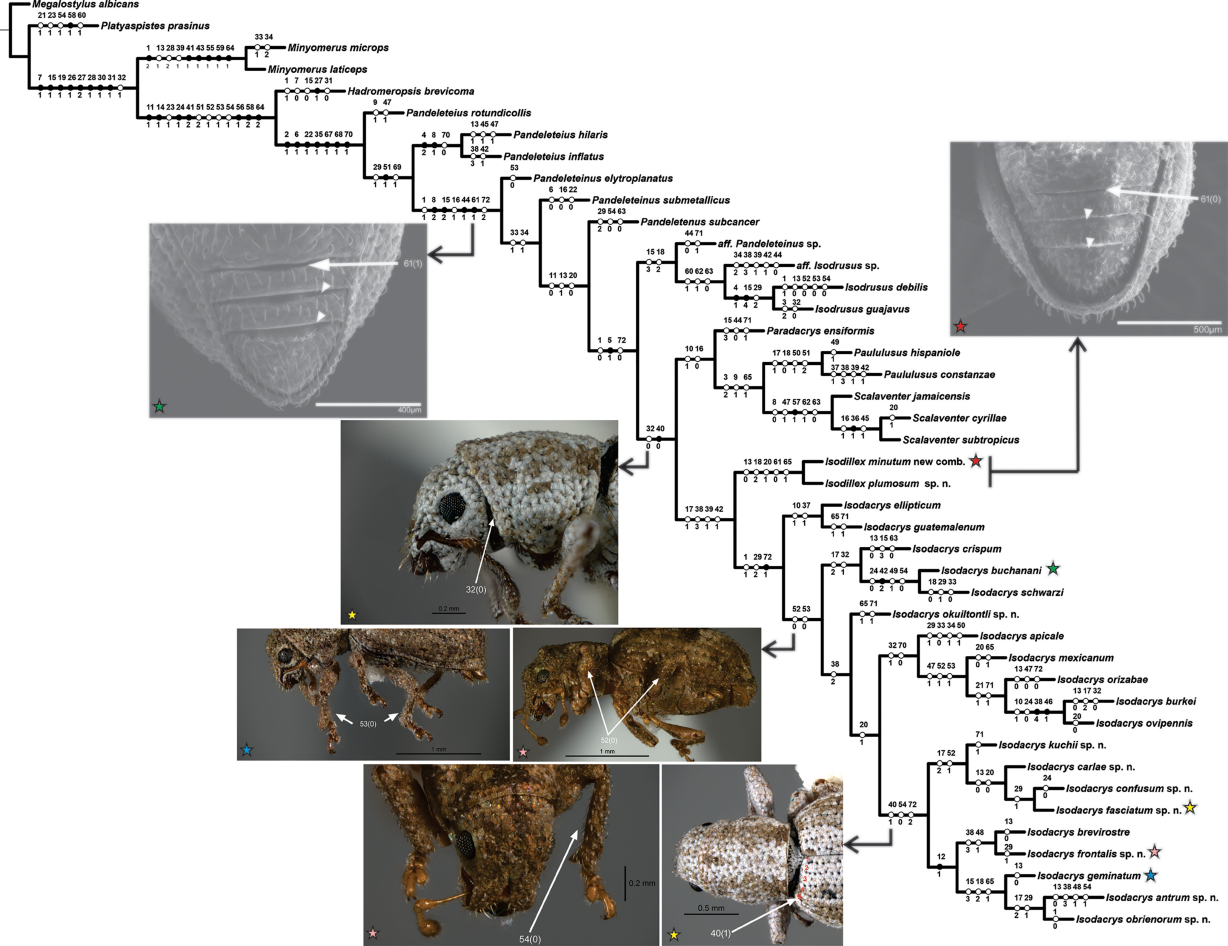
One of the two most parsimonious cladograms (*L* = 242, CI = 0.39, RI = 0.70) with preferred character state optimization (ACCTRAN: chars. 7, 15, 16, 31, 47 and 70; DELTRAN: chars. 8, 13, 23, 32, 41, 44, 54, 64 and 71). Black circles represent non-homoplastic character state transformations, whereas white circles represent homoplastic character state transformations. Numbers on and below each circle correspond to character and state codes, respectively. Colored stars indicate which species is illustrating the highlighted character states.

The heuristic search for most parsimonious trees of 43 terminals and 72 discrete characters under equal weights yielded two equally most parsimonious trees (*L* = 242, CI = 0.39, RI = 0.70). The strict consensus adds two steps to the tree length (*L* = 244, CI = 0.38, RI = 0.69), depicting one collapsed node regarding the position of *Pandeleteinus submetallicus* and *P. elytroplanatus* relative to the *Pandeleteinus subcancer*-*Isodacrys antrum* clade (Fig. 1). Bremer and Jackknife values are mapped on internal nodes of the strict consensus tree (Fig. 1). Branch support values were higher in basal clades and particular West Indian genera but lower on deeper clades related with *Isodacrys* species relationships. One of the two most parsimonious trees (Fig. 2) was chosen to illustrate character state optimizations (57 unambiguous, 6 ACCTRAN and 9 DELTRAN, see Agnarsson & Miller, 2008).

*Isodacrys* was recovered as monophyletic, including a total of 20 species in five main clades (Fig. 1). *Isodacrys* is supported by one synapomorphy and two homoplastic character states (Fig. 2). The *Isodillex* clade, which is sister to *Isodacrys* and supported by four homoplastic character states (Fig. 2), is proposed to accommodate *Isodillex minutum* (Sharp, 1911), new combination and *Isodillex plumosum* Cortés-Hernández, new species. *Isodillex* was excluded from the present circumscription of *Isodacrys* based on the combination of five homoplastic character states supporting the new taxon (Fig. 2). Some of these character states were considered relevant for taxonomic amendments. The clade *Isodacrys-Isodillex* resulted sister to the clade *Paradacrys*-*Scalaventer*, which occur in the West Indies, based on one synapomorphy and one homoplastic character state.

Regardless of the lack of diagnostic character states supporting the monophyly of *Isodacrys*, the depicted combination of character states supporting it plus more inclusive congruent information on basal clades are relevant enough to circumscribe the genus as here presented (see diagnosis of *Isodacrys*). The exclusion of the *Isodillex* is considered necessary based on the not sulcate condition of the anterior margin of ventrites III, IV and V (char. 61:0 and thus inapplicable entries in chars. 62 and 63; chars. 61:1, 62:0 and 63:1 in *Isodacrys*). Secondary characters that can also be helpful in separating *Isodillex* from *Isodacrys* are dorsal scales contiguous, not overlapping (char. 1:0; char. 1:1 in *Isodacrys*, with reversal in *I. obrienorum*), pronotum in dorsal view as wide as long (char. 29:1; char. 29:2 in *Isodacrys*, with reversals to state 1 in *I. schwarzi*, *I. apicale*, *I. confusum*-*I. fasciatum*, *I. frontalis* and *I. antrum*-*I. obrienorum*) and manubrium shorter than median lobe (char. 72:0; char. 72:1 synapomorphy for *Isodacrys* with reversal to state 0 in *I. orizabae* and with evolutionary transition to state 2 in the *I. kuchii*-*I. obrienorum* clade). Accordingly, *Isodillex* is considered a different taxon, sister group of *Isodacrys*. Otherwise, the circumscription of *Isodacrys* would be vague, needing further ad hoc hypothesis of character state evolution.

Given the terminals and characters included in the present analysis, there are five main clades within *Isodacrys* (Fig. 1). The basal division within the genus is between the *I. ellipticum* clade (Fig. 1), supported by two homoplastic character states (presence of longitudinal carina on the epistome 10:1 and setae of elytra strongly modified, conspicuously longer 37:1), and the *I. crispum*-*I. antrum* clade, which incorporates the remaining *Isodacrys* species, also supported by two homoplastic character states related to the size of the prolegs relative to the metalegs (chars. 52:0 and 53:0).

The second division is within the *Isodacrys crispum*-*I. antrum* clade, divided into two clades, the *I. crispum* clade (Fig. 1) and the clade *I. okuiltontli*-*I. antrum*, supported respectively by two (anterior portion of scrobe at least one fifth shorter than posterior portion 17:2 and presence of postocular vibrissae 32:1) and one (elytra in dorsal view obovate 38:2, but character states 3 and 4 also present within the clade) homoplastic character states. In the *Isodacrys crispum* clade, there is a basal division splitting *I. crispum* from *I. buchanani*-*I. schwarzi*, whereas in the clade *I. okuiltontli*-*I. antrum* there is a split depicting *I. okuiltontli* as the sister taxon of the *I. apicale*-*I. antrum* clade. *Isodacrys buchanani*-*I. schwarzi* is well supported by one unreversed character state (basal margin of elytra angularly emarginated 42:2) and three homoplastic characters (antennae with scape covered with scales 24:0, apical declivity of elytra concave 49:1 and inner margin of protibiae without teeth 54:0).

*Isodacrys apicale*-*I. antrum* clade, supported by one homoplastic character (presence of fovea between eyes 20:1), includes most of the *Isodacrys* species in two main clades. The first clade is *I. apicale* (Fig. 1), supported by the presence of postocular vibrissae (char. 32:1, with reversal in *I. burkei*) and by outer corner of the hemisternites VIII truncate (char. 70:0), while *I. kuchii*-*I. antrum* clade by three homoplastic character states (five visible intervals at base of elytra in dorsal view 40:1, inner margin of protibiae without teeth 54:0, with reversal in *I. antrum*, and manubrium of tegmen longer than median lobe 72:2).

Finally, the *Isodacrys kuchii*-*I. antrum* clade is divided into two clades, *I. kuchii* and *I. brevirostre* (Fig. 1). The *Isodacrys kuchii* clade is supported by two homoplastic character states (anterior portion of scrobe at least one fifth shorter than posterior portion 17:2 and profemora at least one fifth wider than metafemora 52:1). The *Isodacrys brevirostre* clade is supported by one synapomorphy (postrostrum with longitudinal sulci mesad of dorsolateral margins 12:1).

### Character discussion (Figs. 2–7)

**Figure 3:**
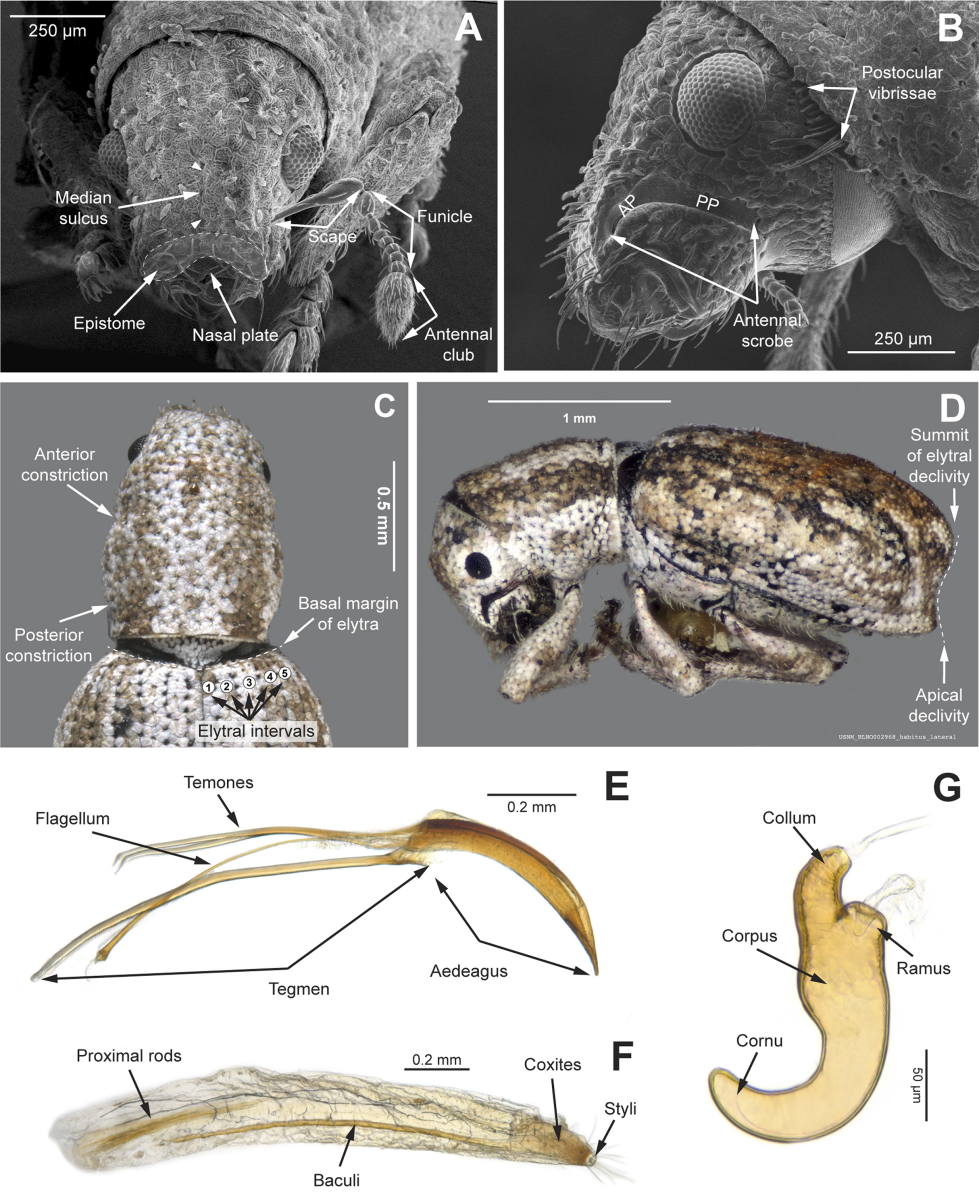
Overview of various adult characters in *Isodacrys*. (A) *Isodacrys brevirostre*, rostrum in dorsal view; small triangles indicate anterior and posterior end of epifrons’ median sulcus; (B) *I. apicale*, rostrum and head in lateral view; acronyms AP and PP refer to anterior and posterior portion of the scrobe, respectively; (C) *I. fasciatum* new species, head, prothorax and base of elytra in dorsal view; (D) *I. schwarzi* lateral habitus taken by Ashton Smith (Usage Rights: CC0 1.0, Public-domain. Available at http://scan-bugs.org/portal/collections/individual/index.php?occid=37439808); (E) *I. kuchii* new species, aedeagus in lateral view; (F) *I. fasciatum* new species, ovipositor in lateral view; (G) *I. frontalis* new species, spermatheca.

**Figure 4:**
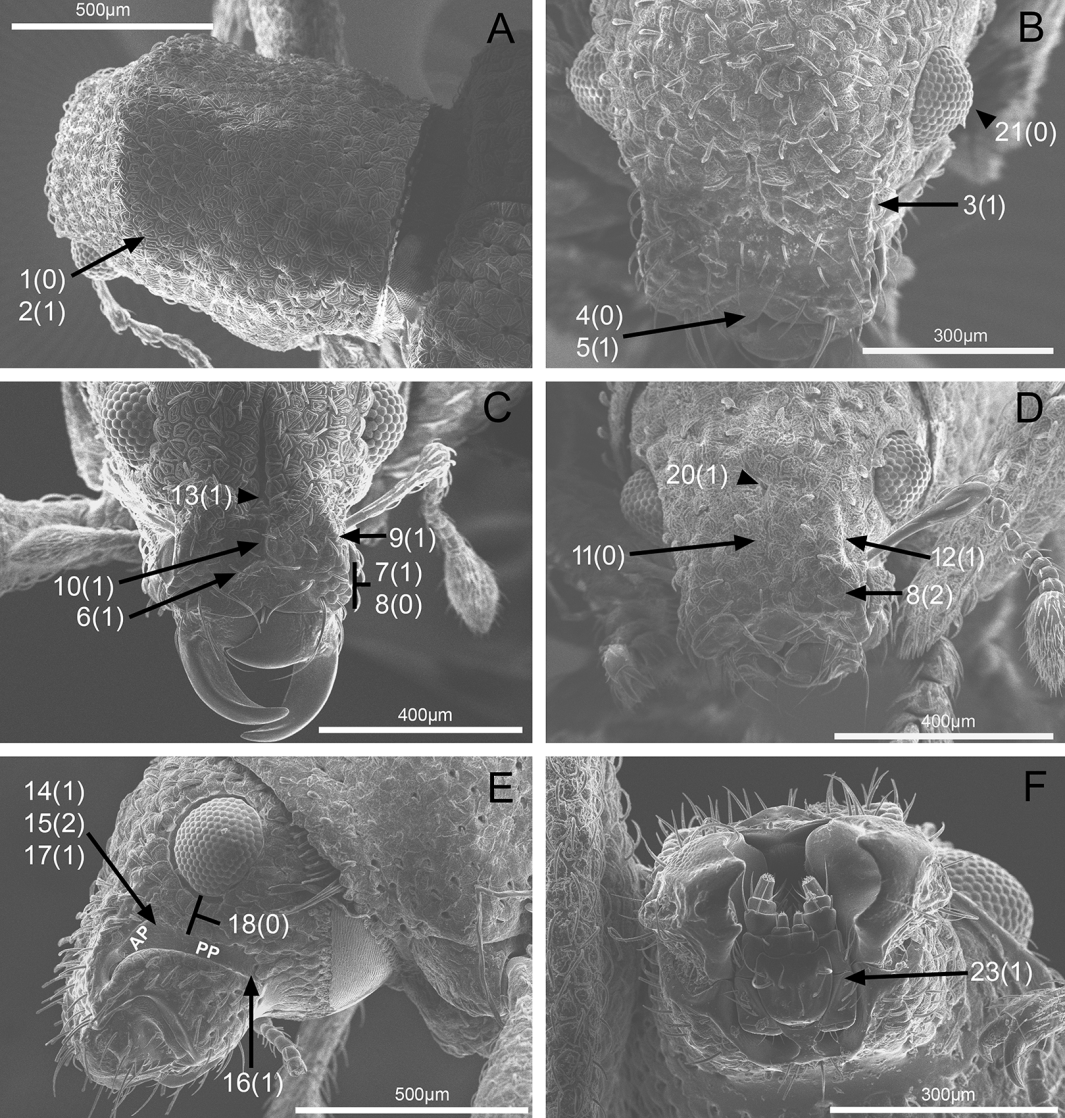
Photographs illustrating some of the characters included in the analysis. (A) *Scalaventer cyrillae*, prothorax in dorsal view; (B) *Isodacrys carlae* new species, rostrum in dorsal view; (C) *S. cyrillae*, rostrum in dorsal view. (D) *I. brevirostre*, rostrum in dorsal view; (E) *I. apicale*, rostrum and head in lateral view; acronyms AP and PP refer to the anterior and posterior portion of the scrobe, respectively; (F) *Paululusus hispaniole*, prementum. Numbers and numbers between parentheses refer to characters and their states, respectively.

**Figure 5:**
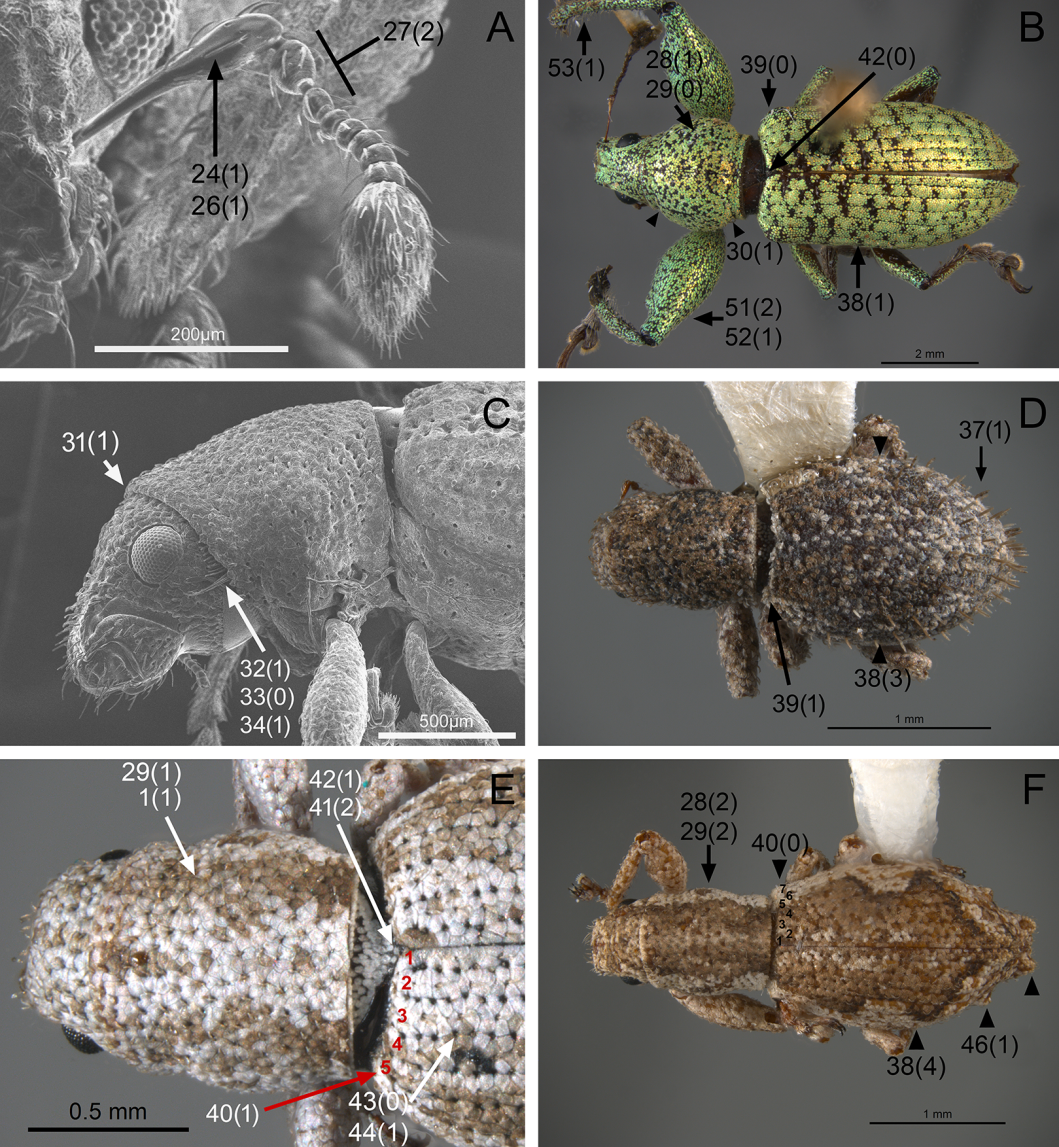
Photographs illustrating some of the characters included in the analysis. (A) *Isodacrys brevirostre*, antenna; (B) *Hadromeropsis brevicoma*, dorsal habitus; (C) *Isodacrys apicale*, rostrum, head and prothorax in lateral view; (D) *I. guatemalenum*, dorsal habitus; (E) *I. fasciatum* new species, head, prothorax and base of elytra in dorsal view; (F) *I. ovipennis*, dorsal habitus. Numbers and numbers between parentheses refer to characters and their states, respectively.

**Figure 6:**
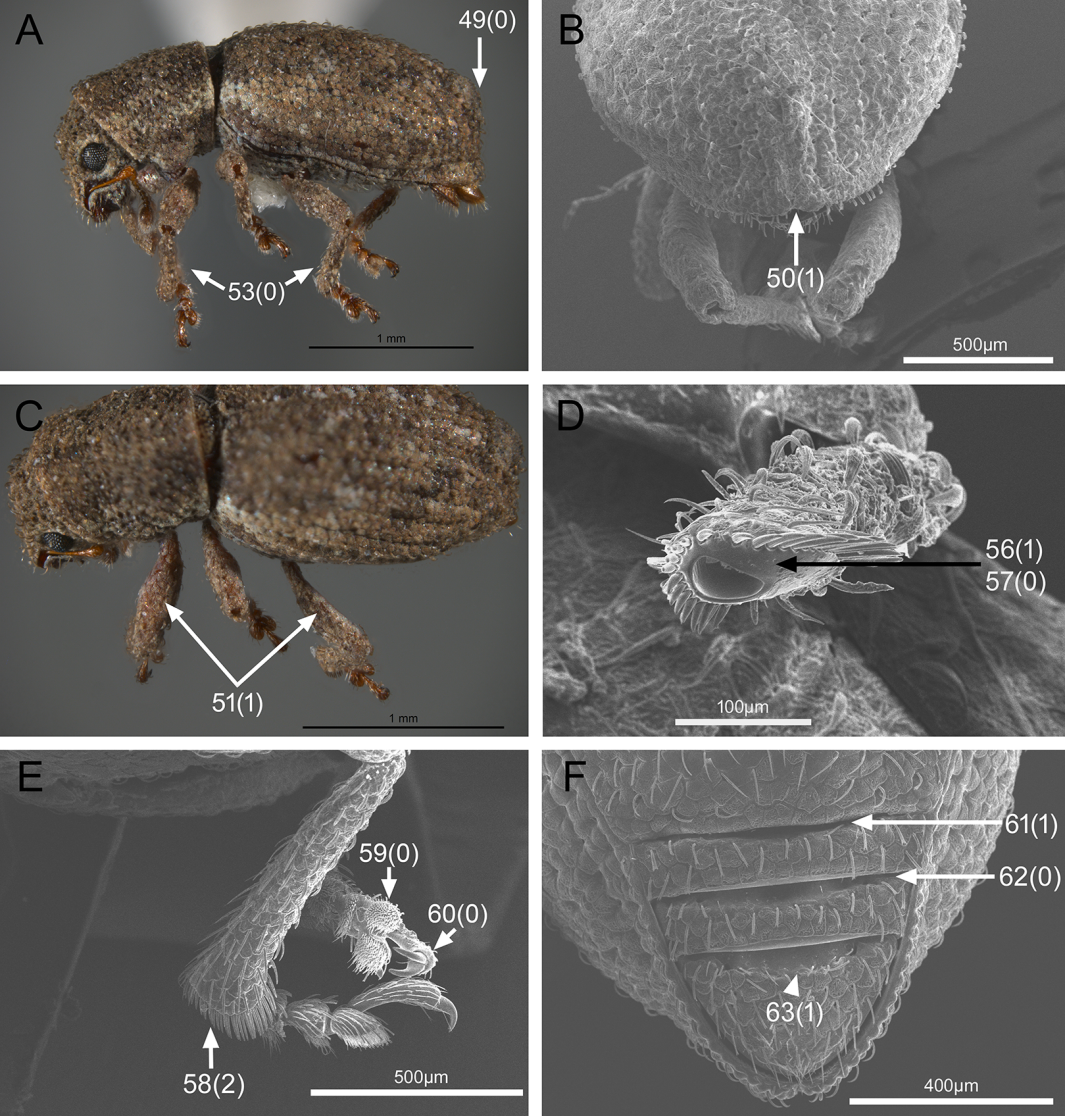
Photographs illustrating some of the characters included in the analysis. (A) *Isodacrys geminatum*, lateral habitus; (B) *I. apicale*, apex of elytra; (C) *I. geminatum*, dorsolateral habitus showing femora in dorsal view; (D) *I. frontalis*, apex of metatibia from tarsal socket view; (E) *I. mexicanum*, apex of metatibia; (F) *I. buchanani*, ventrites two, three, four and five. Numbers and numbers between parentheses refer to characters and their states, respectively.

**Figure 7:**
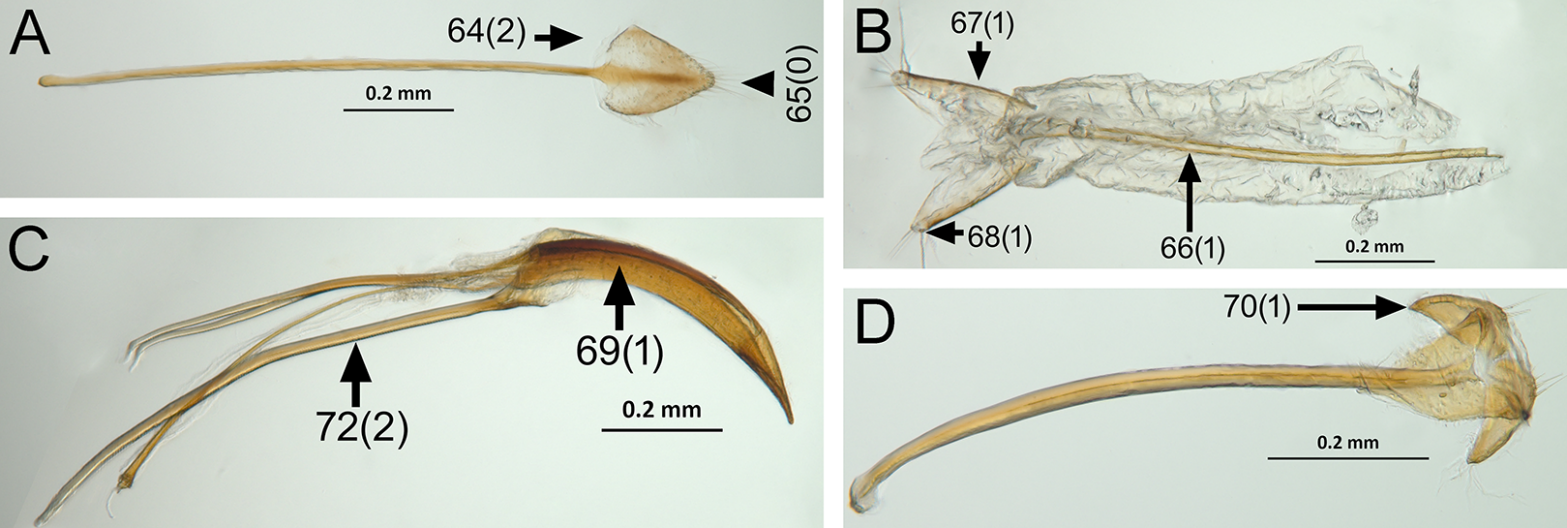
Photographs illustrating some of the characters included in the analysis. (A) *Isodacrys confusum* new species, female sternite VIII in dorsal view; (B) *I. kuchii* new species, ovipositor in dorsal view; (C) *I. kuchii* new species, aedeagus in lateral view; (D) *I. kuchii* new species, male hemisternites VIII and sternite IX in dorsal view. Numbers and numbers between parentheses refer to characters and their states, respectively.

In the following section, characters included in the phylogenetic analysis are presented and discussed. The characters are divided in subheadings: general appearance; rostrum; antennal scrobes; head; mouthparts; antenna; prothorax; elytra; legs; abdomen; and terminalia. An introductory section for each subheading is presented with highlights and explanations (when necessary) of the characters and the character states of either *Isodacrys* and/or outgroups included. The characters are presented in the following sequence: (1) character number, (2) character description, (3) character statement (character states, see Sereno, 2007), (4) additiveness (for multistate characters only), (5) applicability (when necessary; e.g., reductive coding), (6) similar characters in other studies, (7) optimization of character states in the MPT’s or/and synapomorphies, (8) character statistics (length, consistency index, retention index), and (9) additional information (particular comments for the character). See Fig. 2 for preferred character state optimizations. See Fig. 3 for an overview of several adult morphological features in *Isodacrys*, which may apply to other genera; Figs. 4–7 are referred for character states illustrated.

*General appearance* (Figs. 4–5)

The arrangement and shape of the scales in New World Tanymecini are complex along the body, varying even within the same structure (e.g., along elytra, see Howden, 1959). Variation is more evident along fasciae or vittae, when present, and along the apical declivity of the elytra. Considering scales as serialogs (see Ochoterena et al., 2019), character states proposed are based on the dominant patterns presented in Jansen & Franz (2015). The shape of the scales is defined as subcircular when their margin is evenly rounded, whereas polygonal refers to scales with angulate margins, composed by at least three sides. Polygonal scales vary along the body in form and number of sides.

1. Dorsal habitus, scales, arrangement: (0) Contiguous, not overlapping (Fig. 4A); (1) variously overlapping non-linearly (Fig. 5E); (2) only overlapping posteriorly. Non-additive. See character 1 by Jansen & Franz (2015). State 0 convergently present in *Megalostylus albicans*-*Pandeleteius inflatus*, the clade aff. *Pandeleteinus* sp.-*Isodillex* and in *Isodacrys obrienorum*; state 1 convergently present in *Hadromeropsis brevicoma*, the *Pandeleteinus sumetallicus*-*P. subcancer* clade, *Isodrusus debilis* and *Isodacrys*; state 2 synapomorphy for *Minyomerus* (l: 7, ci: 0.28, ri: 0.70).

2. Habitus dorsal, scales, shape: (0) Subcircular; (1) polygonal (Fig. 4A). Character state 1 synapomorphic for the *Pandeleteius*-*Isodacrys* clade (l: 1, ci: 1, ri:1).

*Rostrum* (Figs. 3–4)

The dorsal area of the rostrum in tanymecine weevils can be generally divided into three more or less defined regions from the apex to the frons (Fig. 3A): nasal plate, epistome and epifrons (postrostrum). These regions are herein described to propose homology statements.

The nasal plate consists of a defined, bare area located at the tip of the rostrum, between or slightly in front of the insertion of the antennae, at the apical margin of the epistome (Pierce, 1913; Vaurie, 1963; Franz, 2012). We agree with this perspective and therefore it is treated as a structure different from the epistome (Girón & Franz, 2010; Lanteri & Del Río, 2017; Girón & Howden, 2019). It can be delimited posteriorly from the remainder of the rostrum by the epistomal setae, which indicate the anterior end of the epistome and the beginning of the nasal plate (Fig. 3A). In many Neotropical entimines, the posterior margin of the nasal plate can be elevated, forming a carina (Vaurie, 1963). Howden (1959) first used the term nasal plate for Tanymecini as “the sclerite that is within the apical emargination or extend forward from the apex of the beak”, considering it as a synonym of the epistome. In the literature, the terms nasal plate and epistome have been used to refer to the same structure or different parts of the apical region of the rostrum (Pierce, 1913; Blatchley & Leng, 1916; Howden, 1959; Anderson, 2002; Franz, 2012; Oberprieler, Anderson & Marvaldi, 2014, Jansen & Franz, 2015, 2018; Marvaldi et al., 2018; Girón & Howden, 2019). In the present study, the apical emargination refers to the posterior margin of the nasal plate. Regarding the issue as to whether they are either the same or different structures, Howden (1966) avoided the term nasal plate. The epistome constitutes the reduced frontoclypeal margin or sclerite directly behind the labrum (Nichols, 1989). Although the labrum is considered absent in Curculionidae, it is unclear whether the absent condition represents a complete loss or variable fusions of the labrum and clypeus, and whether the epistomal lobe is a derived modification of the frons or a remnant of the clypeus or labrum (Davis, 2017). Herein, based on the studied species, the epistome is considered as the dorsal area of the rostrum located between the antennal insertions, distinguished by the presence of modified scales (smaller, of different shape and color and sometimes more scattered compared to remaining scales on the rostrum). Additionally, the epistome is usually irregularly covered by erect to semierect pale setae which are set in shallow foveae (Fig. 3A). Finally, the epifrons comprises the proximal region of the rostrum, extending to the anterior margin of the eyes (Oberprieler, Anderson & Marvaldi, 2014). In some tanymecines, there is a fovea at the posterior margin of the epistome and beginning of the epifrons (at anterior end of the median sulcus [median line in Howden’s terminology], when present, Fig. 3A). In several West Indian representatives this fovea is anteriorly bifurcated, forming a Y-shaped depression (Howden, 1970). This fovea could represent a rudimentary/vestigial suture delimiting the typically modified apical region of the rostrum (constituted by the epistome and the nasal plate) in Tanymecini.

The rostrum is always directed ventrally in *Isodacrys*, forming an angle between the ventral outline of the rostrum and the head, varying from obtusely angled to almost right angled. In the outgroups the rostrum can be directed ventrally or virtually in line with the main body axis.

3. Rostrum, orientation of dorsolateral margins: (0) Convergent anteriorly; (1) subparallel (Fig. 4B); (2) divergent towards apex. Additive. See character 22 in Franz (2012), character 8 in Girón & Franz (2012), character 9 in Lanteri & Del Río (2017) and character 0 in Del Río et al. (2018). State 2 is convergently present in *Isodrusus guajavus* and the *Paululusus*-*Scalaventer* clade (l: 3, ci: 0.66, ri: 0.80). In *Isodacrys* the dorsolateral margins of the rostrum are subparallel. Nonetheless, Howden (1961) considered that some *Isodacrys* species have dorsolateral margins of the rostrum slightly convergent anteriorly. According to our observations, this varies intraspecifically and in a way almost imperceptible to consider the dorsolateral margins convergent anteriorly in *Isodacrys*.

4. Rostrum, anterior margin of nasal plate, shape: (0) Emarginate (Fig. 4B); (1) straight; (2) produced anteriorly. Non-additive. See character 3 in Marvaldi et al. (2018). Character state 1 synapomorphy for the *Isodrusus debilis*-*I. guajavus* clade; character state 2 synapomorphy for the *Pandeleteius hilaris*-*P. inflatus* clade (l: 2, ci: 1, ri: 1). Homology statement among these character states is based on topological correspondence.

5. Emargination of nasal plate, degree: (0) Strongly emarginate; (1) slightly indented (Fig. 4B). Character state 1 synapomorphy for the aff. *Pandeleteinus* sp.-*Isodacrys* clade (l: 1, ci: 1, ri: 1). The anterior margin of the nasal plate in *Isodacrys* is almost straight, at most slightly directed inward medially, where it bears a small indentation, sometimes irregular.

6. Rostrum, posterior margin of nasal plate, carina: (0) Absent, posterior margin flat; (1) present (Fig. 4C). See character 10 in Jansen & Franz (2015) and character 16 in Lanteri & Del Río (2017). Character state 1 synapomorphy for the *Pandeleteius*-*Isodacrys* clade, with reversal in *Pandeleteinus submetallicus* (l: 2, ci: 0.50, ri: 0.80). Howden (1961) described several species of *Isodacrys* as having the apical emargination (which herein is termed posterior margin of the nasal plate) not carinate, but according to our observations, it is actually carinate in all *Isodacrys* species. The degree to which the posterior margin is carinate varies among individuals and species.

7. Rostrum, epistome, degree of development: (0) Indistinct; (1) distinct (Fig. 4C). ACCTRAN optimization is preferred, as DELTRAN suggests parallel origins of the epistome. Thus, character state 1 synapomorphy for the Tanymecini clade, with reversal in *Hadromeropsis brevicoma* (l: 2, ci: 0.50, ri: 0.50).

8. Rostrum, epistome, constitution relative to remainder of rostrum: (0) Strongly concave (Fig. 4C); (1) obliquely angled; (2) in a continuous plane with (Fig. 4D). Non-additive. Coded as inapplicable when the epistome is indistinct (see character 7, state 0). See character 10 in Franz (2012). DELTRAN optimization preferred in accordance with the assumption of non-additiveness of character states. Thus, state 0 present in *Minyomerus* and *Pandeleteius rotundicollis*; state 1 synapomorphy for the *Pandeleteius hilaris*-*P. inflatus* clade; state 2 synapomorphy for the *Pandeleteinus*-*Isodacrys* clade, with reversal to state 0 in *Scalaventer* (l: 3, ci: 0.66, ri: 0.83). Character state 2, present in *Isodacrys* and relatives, can be slightly depressed. This varies among individuals and species.

9. Rostrum, epistome, posterior margin: (0) Indistinct; (1) keeled (Fig. 4C). Coded as inapplicable when the epistome is indistinct (see character 7, state 0). See character 14 in Franz (2012). Character state 1 convergently present in *Pandeleteius rotundicollis* and the *Paululusus*-*Scalaventer* clade (l: 2, ci: 0.50, ri: 0.80).

10. Rostrum, epistome, longitudinal carina extending along epistome, reaching posterior margin of nasal plate: (0) Absent; (1) present (Fig. 4C). Coded as inapplicable when the epistome is indistinct (see character 7, state 0). State 1 convergently present in the *Paradacrys*-*Scalaventer* clade, the *Isodacrys ellipticum*-*I. guatemalenum* clade and the *I. burkei*-*I. ovipennis* clade (l: 3, ci: 0.33, ri: 0.77).

11. Constitution of epifrons: (0) Flat to slightly depressed mesally (Fig. 4D); (1) concave. State 1 synapomorphy for the *Hadromeropsis brevicoma*-*Pandeleteinus submetallicus* clade, with reversal to state 0 for the *Pandeleteinus subcancer*-*Isodacrys* clade (l: 2, ci: 0.50, ri: 0.80). In *Isodacrys* the epifrons is flat to slightly depressed mesally when the median sulcus and/or foveae are present. This varies among individuals and species.

12. Epifrons, longitudinal sulci mesad of dorsolateral margins: (0) Absent; (1) present (Fig. 4D). Character state 1 synapomorphy for the *Isodacrys brevirostre*-*I. antrum* clade (l: 1, ci:1, ri: 1). These longitudinal sulci are short, extending from near the antennal insertion to the deflection of the scrobe.

13. Rostrum, epifrons, fovea: (0) Absent; (1) present (Fig. 4C). DELTRAN optimization is preferred because ACCTRAN postulates several origins of the fovea in *Isodacrys*. Thus, state 1 convergently present in *Minyomerus*, *Pandeleteius hilaris* and the *Pandeleteinus subcancer*-*Isodacrys* clade, with subsequent reversals in *Isodrusus debilis*, *Isodillex*, *Isodacrys crispum*, *I. orizabae*, *I. burkei*, *I. carlae*-*I. fasciatum* clade, *I. brevirostre*, *I. geminatum* and *I. antrum* (l: 12, ci: 0.08, ri: 0.38).

*Antennal scrobes* (Figs. 3–4)

The scrobes can be curved or angled in the species included in this analysis (Figs. 3B and 4E). When angled, two discernible parts of the scrobe can be distinguished relative to orientation of the rostrum: an anterior and a posterior part (Fig. 3B, AP and PP respectively; called horizontal and vertical portion, respectively, by Howden, 1961, 1970). In some taxa the scrobe can reach the ventral part of the rostrum. The dorsal margin of the scrobe was taken as reference guide to determine the shape of the scrobe; distance from the posterior portion of the scrobe to the eye was considered at the nearest point between them.

14. Rostrum, scrobe, vestiture: (0) Present; (1) absent, scrobe glabrous (Fig. 4E). Character state 1 synapomorphy for *Hadromeropsis*-*Isodacrys* clade (l: 1, ci: 1: ri: 1). The scrobes are always glabrous in *Isodacrys*, whereas in the outgroups it varies from glabrous to covered by different types of vestiture (setae, scales, plumose scales).

15. Rostrum, scrobe in lateral view, dorsal margin, shape: (0) Evenly curved, directed to compound eye; (1) strongly curved, directed to ventral surface of rostrum; (2) obtusely angled (Fig. 4E); (3) right-angled; (4) acutely angled. Additive. See characters 14 and 12 in Lanteri & Del Río (2017) and in Marvaldi et al. (2018), respectively. ACCTRAN optimization is preferred because DELTRAN postulates two origins of character state 1. Thus, character state 1 synapomorphy for the Tanymecini clade with reversal to state 0 in *Hadromeropsis brevicoma* and subsequent evolutionary transition to state 2 in the *Pandeleteinus*-*Isodacrys* clade; state 3 convergently present in the aff. *Pandeleteinus* sp.-aff. *Isodrusus* sp. clade, *Paradacrys ensiformis*, *Isodacrys crispum* and the *I. geminatum*-*I. obrienorum* clade; character state 4 synapomorphy for the *Isodrusus debilis*-*I. guajavus* clade (l: 8, ci: 0.50, ri: 0.77).

16. Rostrum, ventral margin of scrobe, length relative to ventral surface of rostrum: (0) Ending before ventral surface; (1) reaching ventral surface (Fig. 4E). ACCTRAN optimization is preferred to preserve homology of character state 1. Thus, character state 1 synapomorphy for the *Pandeleteinus*-*Isodacrys* clade with reversals in *Pandeleteinus submetallicus* and the *Paradacrys ensiformis*-*Scalaventer jamaicensis* clade (l: 4, ci: 0.25, ri: 0.75).

17. Rostrum, scrobe, anterior portion, length relative to posterior portion: (0) At least one fifth longer; (1) subequal (Fig. 4E); (2) at least one fifth shorter. Non-additive. Coded as inapplicable when scrobes are curved (see character 15, states 0 and 1). State 0 present in *Pandeleteius inflatus*; state 1 convergently present in *Paululusus* and the *Isodillex*-*Isodacrys geminatum* clade; state 2 convergently present in the *Pandeleteinus*-*Scalaventer* clade, the *Isodacrys crispum-I. schwarzi* clade, *I. burkei*, the *I. kuchii*-*I. fasciatum* clade and the *I. antrum*-*I. obrienorum* clade (l: 7, ci: 0.28, ri: 0.61).

18. Rostrum, scrobe, posterior portion, distance to eye: (0) At least one fifth longer than width of scrobe (Fig. 4E); (1) as wide as width of scrobe; (2) at least one fifth narrower than width of scrobe. Non-additive. Coded as inapplicable when scrobes are curved (see character 15, states 0 and 1). State 0 convergently present in *Pandeleteinus elytroplanatus*-*P. submetallicus*, *Paululusus*, *Isodacrys schwarzi*, *I. apicale*-*I. ovipennis*, *I. carlae*-*I. fasciatum* and *I. frontalis*; state 1 convergently present in *Pandeleteius inflatus*, *Pandeleteinus subcancer*-*Isodacrys buchanani*, *I. kuchii* and *I. brevirostre;* state 2 convergently present in the aff. *Pandeleteinus* sp.-*Isodrusus guajavus* clade, *Isodillex*, *Isodacrys okuiltontli* and the *I. geminatum*-*I. obrienorum* clade (l: 11, ci: 0.18, ri: 0.55).

19. Rostrum in lateral view, angle relative to head: (0) Directed anteriorly, in line with main body axis; (1) directed ventrally. Character state 1 synapomorphy for the Tanymecini clade (l: 1, ci: 1: ri: 1).

*Head* (Fig. 4)

A fovea can be present between the eyes (at posterior end of the median sulcus, when present). The convexity of eyes is variable within *Isodacrys*, from slightly protruding to strongly convex, always more convex posteriorly.

20. Head, frons, fovea between eyes: (0) Absent; (1) present (Fig. 4D). State 1 convergently present in *Platyaspistes prasinus*, *Hadromeropsis brevicoma*-*Pandeleteinus submetallicus*, *Scalaventer cyrillae*, *Isodillex* and the *Isodacrys apicale*-*I. obrienorum* clade, with subsequent reversals in *Minyomerus*, the *Pandeleteinus subcancer*-*Isodacrys okuiltontli* clade, *I. mexicanum*, *I. ovipennis* and the *I. carlae*-*I. fasciatum* clade (l: 9, ci: 0.11, ri: 0.55).

21. Head, eyes, convexity: (0) Strongly convex (Fig. 4B); (1) flat to slightly convex. See Marvaldi & Lanteri (2005), character 3 in Girón & Franz (2010), character 36 in Franz (2012), character 4 in Del Río et al. (2018) and character 27 in Marvaldi et al. (2018). State 1 convergently present in *Platyaspistes prasinus* and the *Isodacrys orizabae*-*I. ovipennis* clade (l: 2, ci: 0.50, ri: 0.66).

*Mouthparts* (Fig. 4)

The mandibles of Entimine weevils can be covered by setae and scales, the latter covering either partially or apparently the entire surface of the mandibles. In *Isodacrys* (as well as in other tanymecines), the mandibles are covered by setae, without scales. The presence of scales in the mandibles has been highlighted to separate some genera of New World Tanymecini (Howden, 1982).

Exposure of the maxillary palpi within Entiminae has been observed in several tribes (Anderson, 2002; Girón & Franz, 2010; Marvaldi et al., 2014) that are not closely related. Therefore, this condition within Tanymecini as in other tribes could represent isolated cases of reversals, if adelognathy (prementum completely covering maxillae) evolved early in the subfamily and was reversed secondarily (Marvaldi et al., 2014). Nonetheless, we do not follow the traditional nomenclature (adelognathous and phanerognathous conditions, respectively) to refer to these states because there are different opinions about the limits between them (Thompson, 1992). Howden (1959) had already noticed that the lower part of the maxillae (i.e., cardo, stipes) is visible for *Pandeleteius* and *Pandeleteinus* and this is also applicable to *Isodacrys* and other tanymecine genera (see also Howden, 1993b).

22. Mouthparts, mandibles, scales: (0) Present; (1) absent. Character state 1 synapomorphy for *Pandeleteius*-*Isodacrys* clade, with reversal in *Pandeleteinus submetallicus* (l: 2, ci: 0.50, ri: 0.80).

23. Mouthparts, prementum relative to maxillae: (0) Prementum completely concealing maxillae; (1) prementum incompletely concealing maxillae (Fig. 4F). See character 19 in Marvaldi et al. (2018). DELTRAN optimization is preferred based on the assumption of no immediate relationship with *Platyaspistes prasinus* (Leptopiini). Thus, character state 1 convergently present in *P. prasinus* and the *Hadromeropsis*-*Isodacrys* clade (l: 2, ci: 0.50, ri: 0.50).

*Antenna* (Figs. 3, 5)

The antennal scape in New World Tanymecini is covered by interspersed setae along its surface (Fig. 5A). Additionally, some clades within *Isodacrys* also have appressed scales, restricted to the apical half of the scape. In the outgroups when scales are present, they evenly cover the scape. The shape of the scape in the studied Tanymecini is consistently capitate, abruptly thickened at apex (Nichols, 1989; Fig. 3A). Additionally, funicular antennomeres I and II are usually of different shape and longer than remaining funicular antennomeres (Fig. 3A).

24. Antenna, scape, squamose vestiture: (0) Present; (1) absent (Fig. 5A). See character 2 in Lanteri & Del Río (2017). State 0 convergently present in *Megalostylus albicans*-*Minyomerus*, the *Isodacrys buchanani*-*I. schwarzi* clade, the *I. burkei*-*I. ovipennis* clade and *I. confusum*; state 1 synapomorphy for the *Hadromeropsis brevicoma*-*Isodacrys* clade (l: 4, ci: 0.25, ri: 0.62).

25. Antenna, scape, squamose vestiture, arrangement: (0) Scape evenly covered by scales; (1) scape with scales restricted to dorsal surface or apex (Fig. 3D). State 1 convergently present in *Isodacrys buchanani*-*I. schwarzi*, *I. burkei*-*I. ovipennis* and *I. confusum* (l: 1, ci: 1, ri: 1).

26. Antenna, scape, shape: (0) Clavate, gradually thickening towards apex; (1) capitate, abruptly thickened at apical region (Fig. 5A). See character 30 in Franz (2012), character 12 in Girón & Franz (2012), character 29 in Lanteri & Del Río (2017), character 6 in Del Río et al. (2018) and character 30 in Marvaldi et al. (2018). Character state 1 synapomorphy for the Tanymecini clade (l: 1, ci: 1, ri: 1).

27. Antenna, funicular antennomere I, length relative to funicular antennomere II: (0) Equal; (1) shorter; (2) longer (Fig. 5A). Non-additive. See character 8 in Del Río et al. (2018) and character 34 in Marvaldi et al. (2018). Character state 1 autapomorphy for *Hadromeropsis brevicoma*; character state 2 synapomorphy for the Tanymecini clade (l: 2, ci: 1, ri: 1).

*Prothorax* (Figs. 3–5)

In *Isodacrys*, the shape of the pronotum is always subcylindrical, with constrictions near anterior and posterior margins (Fig. 3C), making the sides of the pronotum between these constrictions sinuate (Howden, 1961).

The presence of postocular vibrissae has been a traditional character for distinguishing Tanymecini from other tribes (Lacordaire, 1863; LeConte & Horn, 1876; Van Emden, 1944; Howden, 1970; Fig. 3B). Nevertheless, this character is evidently homoplastic because there is no trace of postocular vibrissae in several genera and species of New World Tanymecini, and vibrissae are also present in other tribes of broad-nosed weevils (e.g., Anomophthalmini; Morrone, 1998). These are likely used to clean the eyes of debris by pulling the rostrum down and back. Thus, it may have significant adaptive value and be subject to convergent evolution (Del Río & Lanteri, 2019b) or retentions. Notwithstanding, we consider that there may be a latent phylogenetic signal at this level. Postocular vibrissae characters follow observations made in previous works (Howden, 1961, 1969; Franz, 2012; Jansen & Franz, 2015; Marvaldi et al., 2018). Postocular vibrissae are considered vestigial when their length does not surpass at least half length of the compound eye. Additionally, vestigial postocular vibrissae are thin and usually only visible with high magnification. The well-developed ones are at least two thirds length of the eye. Franz (2012) considered the postocular vibrissae in members of Eustylini, which are set inward in a postocular lobe and as a fringe (see Franz, 2012, character 48) as non-homologous from members of *Pandeleteius*, which are finer, fewer, more arched, and more externally situated. Following Franz’s reasoning, characters of the postocular vibrissae were coded as inapplicable for *Platyaspistes prasinus*, which presents postocular lobes. According to the most parsimonious trees, there is a trend to the reduction/loss of the postocular vibrissae within New World Tanymecini.

28. Pronotum in dorsal view, shape: (0) Trapezoidal, sides convergent from the base towards the apex; (1) subglobose, sides strongly curved, maximum width near midlength (Fig. 5B); (2) subcylindrical, sides at most slightly sinuate (Fig. 5F). Additive. See Howden (1959, 1961), character 38 in Lanteri & Del Río (2017), character 10 in Del Río et al. (2018) and character 38 in Marvaldi et al. (2018). State 1 synapomorphy for Tanymecini (although only present in the *Hadromeropsis brevicoma*-*Pandeleteinus submetallicus* clade), with subsequent convergent transition to state 2 in *Minyomerus*, *Pandeleteinus elytroplanatus* and the *P. subcancer*-*Isodacrys* clade (l: 4, ci: 0.50, ri: 0.71).

29. Pronotum in dorsal view, length/width ratio: (0) Wider than long (Fig. 5B); (1) as wide as long (Fig. 5E); (2) longer than wide (Fig. 5F). Additive. Character state 1 convergently present in the *Pandeleteius hilaris*-*Isodillex* clade, *Isodacrys schwarzi*, *I. apicale*, the *I. confusum-I. fasciatum* clade, *I. frontalis* and the *I. antrum*-*I. obrienorum* clade; character state 2 convergently present in *Pandeleteinus subcancer*, the *Isodrusus debilis*-*I. guajavus* clade and the *Isodacrys* clade (l: 9, ci: 0.22, ri: 0.65). As wide as long is limited to ratios of length/width not surpassing one tenth longer/wider.

30. Pronotum in dorsal view, constrictions near anterior and posterior margins: (0) Absent; (1) present (Figs. 3C and 5B). Character state 1 synapomorphy for the Tanymecini clade (l: 1, ci: 1, ri: 1).

31. Prothorax in lateral view, anterior margin, shape: (0) Nearly straight, at level with ventral margin; (1) oblique, dorsally produced anteriorly (Fig. 5C). ACCTRAN optimization is preferred because it preserves ancestral homology of character state 1 for the Tanymecini clade, with reversal in *Hadromeropsis brevicoma* (l: 2, ci: 0.50, ri: 0.50).

32. Prothorax, postocular vibrissae: (0) Absent (Fig. 2); (1) present (Fig. 5C). See Howden (1959, 1961, 1970), character 48 in Franz (2012) and character 37 in Marvaldi et al. (2018). DELTRAN optimization is preferred as it posits the presence of postocular vibrissae as an ancestral state in the Tanymecini clade. Thus, state 1 convergently present in the *Minyomerus*-*Isodrusus debilis* clade, the *Isodacrys crispum*-*I. schwarzi* clade and the *I. apicale*-*I. ovipennis* clade, the latter with reversal in *I. burkei* (l: 6, ci: 0.16, ri: 0.68). According to our MPT’s the most plausible scenario within Tanymecini is that absence of postocular vibrissae is a derived condition in *Isodacrys*, *Isodillex*, and the West-Indian clade.

33. Prothorax, postocular vibrissae, number: (0) More than six postocular vibrissae (Fig. 5C); (1) from one to six postocular vibrissae. Coded as inapplicable when the postocular vibrissae are absent (see character 32, state 0). State 0 present in *Minyomerus laticeps*-*Pandeleteinus elytroplanatus*; state 1 convergently present in *Minyomerus microps* and the *Pandeleteinus submetallicus*-*Isodacrys* clade, with subsequent reversals in *Isodacrys schwarzi* and *I. apicale* (l: 4, ci: 0.25, ri: 0.57).

34. Prothorax, postocular vibrissae, number of well-developed postocular vibrissae: (0) More than five well-developed postocular vibrissae; (1) from one to five well-developed postocular vibrissae (Fig. 5C); (2) all postocular vibrissae short, vestigial. Additive. Coded as inapplicable when postocular vibrissae are absent (see character 32, state 0). State 0 present in *Minyomerus laticeps*-*Pandeleteinus elytroplanatus*; state 1 convergently present in the *Pandeleteinus submetallicus*-*Isodrusus debilis* clade, *Isodacrys apicale* and *I. ovipennis*; state 2 convergently present in *Minyomerus microps*, aff. *Isodrusus* sp. and *Isodacrys crispum*-*I. obrienorum* (l: 7, ci: 0.28, ri: 0.54).

35. Prosternum, intercoxal process, degree of development: (0) Not continuous, divided in two halves (anterior and posterior) not contiguous to each other; (1) continuous. See characters 18 and 19 in Jansen & Franz (2015). Character state 1 synapomorphy for the *Pandeleteius*-*Isodacrys* clade (l: 1, ci: 1, ri: 1). *Isodacrys* species always have the intercoxal process continuous and therefore the procoxae are separated (Howden, 1961).

*Elytra* (Figs. 3, 5, 6)

The vestiture of the elytra in *Isodacrys* is evenly distributed in single rows on the intervals or interspersed irregularly. There are some outgroups where setae are interspersed or absent on some intervals (e.g., *Scalaventer*, see Howden, 1970).

In entimines, humeri absence has been proposed to be associated with thickening and merging of the elytra along the elytral suture, and reductions in the metendosternite and wings (Franz, 2012). Absence of humeri is considered herein when humeral lobes sensu Torre-Bueno (Nichols, 1989) cannot be distinguished. The apterous condition, reflected by the lack of humeri, is constant in *Isodacrys* whereas in other New World Tanymecini it varies interspecifically (Howden, 1970; original observations, e.g., aff. *Isodrusus* sp.). Additionally, in wingless Tanymecini the number of visible intervals at the base of the elytra can vary from five to seven (Figs. 3C, 5E, 5F). This may be related to the development of the humeral angles. Howden (1961) considered that several *Isodacrys* species have partially developed/vestigial humeri because the elytra can be slightly produced at the very base of intervals seven to nine, where humeri take place in winged weevils. These slight elevations are not considered proper humeri herein. The basal margin of the elytra is usually emarginate in Neotropical tanymecines (e.g., Fig. 3C). Although Howden (1961) stated that some *Isodacrys* species have the basal margin of the elytra straight, the epipleural intervals of the elytra always extend more anteriorly than the dorsal ones. The shape of the emargination of the basal margin of the elytra is usually more clear-cut in winged genera. Finally, elytral intervals and the apical declivity (Figs. 3D, 5F, 6A) can provide additional characters. In some tanymecines, the odd-numbered intervals can be convex relative to the even-numbered intervals (Howden, 1970; Lanteri & Del Río, 2017). Within *Isodacrys* the intervals are evenly flat, but some species display basal or apical elevations and/or depressions (Howden, 1961). The elytra can be evenly arcuate from base to apex or deflected along apical third, forming a discernible declivity (Howden, 1961). When the apical declivity is evident, the shape below summit of the declivity can be oblique to different degrees to almost straight (Fig. 6A) or strongly concave (Fig. 3D) in some *Isodacrys* species as in other tanymecines (Howden, 1961, 1970).

36. Elytra, setae, arrangement: (0) In single rows, present in all intervals; (1) in single rows, not present in all intervals. Inapplicable when setae are interspersed. Character state 1 synapomorphy for the *Scalaventer cyrillae*-*S. subtropicus* clade (l: 1, ci: 1, ri: 1).

37. Elytral setae relative to remaining setae of dorsal surface of head and pronotum: (0) At most slightly longer; (1) strongly modified, conspicuously longer (Fig. 5D). State 1 convergently present in *Paululusus constanzae* and the *Isodacrys ellipticum*-*I. guatemalenum* clade (l: 2, ci: 0.50, ri: 0.50).

38. Elytra in dorsal view, shape: (0) Oblong, sides converging to apex from midlength; (1) subcylindrical, sides converging to apex near apical fourth (Fig. 5B); (2) obovate, widest point before midlength; (3) subelliptical, widest point near midlength (Fig. 5D); (4) ovate, widest point after midlength (Fig. 5F). Non-additive. State 1 present in the *Hadromeropsis brevicoma*-*Scalaventer subtropicus* clade; state 2 convergently present in *Minyomerus* and the *Isodacrys okuiltontli*-*I. obrienorum* clade; state 3 convergently present in *Pandeleteius inflatus*, aff. *Isodrusus* sp., *Paululusus constanzae*, the *Isodillex*-*Isodacrys schwarzi* clade, the *I. brevirostre*-*I. frontalis* clade and *I. antrum* (l: 10, ci: 0.40, ri: 0.76).

39. Elytra, humeri: (0) Present (Fig. 5B); (1) absent (Fig. 5D). See character 62 in Franz (2012), character 19 in Girón & Franz (2012), character 51 in Lanteri & Del Río (2017) and character 22 in Del Río et al. (2018). Character state 1 convergently present in *Minyomerus*, aff. *Isodrusus* sp., *Paululusus constanzae* and the *Isodillex*-*Isodacrys* clade (l: 4, ci: 0.25, ri: 0.81).

40. Elytra in dorsal view, visible intervals at base: (0) Seven (Fig. 5F); (1) five (Fig. 5E). Coded as inapplicable when humeri are present (Character 39, state 0). State 0 synapomorphy for the *Paululusus constanzae*-*Isodacrys ovipennis* clade; state 1 convergently present in *Minyomerus*, aff. *Isodrusus* sp. and the *Isodacrys kuchii*-*I. obrienorum* clade. (l: 2, ci: 0.50, ri: 0.90).

41. Elytra, base, shape: (0) Bisinuate; (1) straight; (2) emarginate (Fig. 5E). Non-additive. See character 50 in Lanteri & Del Río (2017) and character 21 in Del Río et al. (2018). DELTRAN optimization is preferred as it states a common origin of character state 2 for *Hadromeropsis*-*Isodacrys* clade. ACCTRAN optimization implies an ancestral state 1 for Tanymecini clade, which could be problematic because *Minyomerus* has several psammophilic traits (adaptations to sandy environments) (Jansen & Franz, 2015). Thus, character state 1 synapomorphy for *Minyomerus*; character state 2 synapomorphy for the *Hadromeropsis*-*Isodacrys* clade (l: 2, ci: 1, ri: 1).

42. Elytra, base, emargination, shape: (0) Strongly emarginate medially (Fig. 5B); (1) roundly emarginate (Fig. 5E); (2) angularly emarginate. Non-additive. State 0 present in *Hadromeropsis brevicoma*-*Scalaventer subtropicus*; state 1 convergently present in *Pandeleteius inflatus*, aff. *Isodrusus* sp., *Paululusus constanzae* and the *Isodillex*-*Isodacrys* clade; state 2 synapomorphy for the *Isodacrys buchanani*-*I. schwarzi* clade (l: 5, ci: 0.40, ri: 0.78).

43. Elytra, strial punctures, degree of development: (0) Conspicuous, deep (Fig. 5E); (1) inconspicuous. Character state 1 synapomorphy for *Minyomerus* (l: 1, ci: 1, ri: 1). In some species of *Minyomerus* the strial punctures are faint or not evident beneath the appressed scales (Jansen & Franz, 2015).

44. Elytra, striae, type: (0) Striate-punctate; (1) punctate (Fig. 5E). Coded as inapplicable when strial punctures are indistinct (see character 43, state 1). DELTRAN optimization is preferred, which preserves common origin of character state 1. Thus, state 1 synapomorphy for the *Pandeleteinus*-*Isodacrys* clade, with subsequent reversals in aff. *Pandeleteinus* sp., aff. *Isodrusus* sp. and *Paradacrys ensiformis* (l: 4, ci: 0.25, ri: 0.62).

45. Elytra, intervals, convexity: (0) Evenly flat; (1) alternate intervals convex. See character 63 in Franz (2012) and character 59 in Lanteri & Del Río (2017). Character state 1 convergently present in *Pandeleteius hilaris* and the *Scalaventer cyrillae*-*S. subtropicus* clade (l: 2, ci: 0.50, ri: 0.50).

46. Elytra, intervals, swelling at apical junction of intervals two and ten; five and six: (0) Absent; (1) present (Fig. 5F). Character state 1 synapomorphy for the *Isodacrys burkei*-*I. ovipennis* clade (l: 1, ci: 1, ri: 1).

47. Elytra, intervals three to five at base: (0) Not elevated; (1) elevated. ACCTRAN optimization is preferred, which preserves homology statement within *Isodacrys*. Thus, state 1 convergently present in *Pandeleteius rotundicollis*, *P. hilaris*, *Scalaventer* and in the *Isodacrys mexicanum*-*I. ovipennis* clade, the latter with reversal in *I. orizabae* (l: 5, ci: 0.20, ri: 0.42).

48. Elytra, intervals seven to nine at basal sixth, depression: (0) Absent; (1) present. Character state 1 convergently present in the *Isodacrys brevirostre*-*I. frontalis* clade and *I. antrum* (l: 2, ci: 0.50, ri: 0.50).

49. Elytra in lateral view, apical declivity, shape: (0) Oblique to straight (Fig. 6A); (1) concave (Fig. 3D). Coded as inapplicable in species with elytra evenly arcuate. See character 66 in Franz (2012) and character 56 in Lanteri & Del Río (2017). State 1 convergently present in *Paululusus hispaniole* and the *Isodacrys buchanani*-*I. schwarzi* clade (l: 2, ci: 0.50, ri: 0.50).

50. Elytra, apical margins, shape: (0) Conjointly rounded; (1) bisinuate (Fig. 6B). See Howden (1961), character 58 in Lanteri & Del Río (2017) and character 43 in Marvaldi et al. (2018). State 1 convergently present in *Paululusus* and *Isodacrys apicale* (l: 2, ci: 0.50, ri: 0.50).

*Legs* (Figs. 5–6)

Within Tanymecini (as in other entimines), the relative size of the legs varies in different ways, from prolegs distinctly larger than metalegs to prolegs shorter than metalegs. The relative size of mesolegs is not considered because the mesotibiae are always the smallest and the mesofemora are of the same size of the non-enlarged pair. In some species the prolegs can be larger in males than in females (Howden, 1959, 1961, 1970, 1982; Franz, 2012; Jansen & Franz, 2015, 2018). The width of the metafemora was taken from maximum width of the lateral outer face, as many Curculionoidea have the metafemora (and sometimes meso and profemora) with its inner face flattened and curved when seen in dorsal view (see metaleg in Fig. 6C).

Commonly in weevils, there are denticles, often called “teeth”, along the inner margin of the protibiae, which may vary in number and degree of development among individuals or species (Howden, 1959, 1961, 1963, 1970). Some clades within *Isodacrys* may have the protibial inner margin smooth, without teeth. Terminology of the metatibial apex (true corbel, false corbel and simple metatibial apex) follow Oberprieler (2010) and Marvaldi et al. (2014), incorporating the observations made by Thompson (1992) and therefore it is presented as in Cortes-Hernández & Morrone (2019, see figures 1–6). In *Isodacrys* species the metatibial apex is simple. Additionally, the metatibial apex in some Entiminae can bear two different combs of bristles: a distal comb across the apex, oriented transversally to the main axis of tibia, and an ascending dorsal comb on the outer margin of metatibiae, oriented longitudinally to the main axis of tibia (Buchanan, 1939; Anderson, 2002; Marvaldi et al., 2018). Nonetheless, the shape of the distal comb varies; in *Isodacrys* and other Neotropical Tanymecini the distal comb is obliquely arcuate, with two discernible regions: the innermost bears short bristles while the outermost bears long bristles (Fig. 6E). This is apparently related to the shape of the outer apical edge of the tibiae.

51. Legs, profemora, length relative to metafemora: (0) At least one fifth shorter; (1) subequal (Fig. 6C); (2) at least one fifth longer (Fig. 5B). Non-additive. See character 51 in Franz (2012). State 1 synapomorphy for the *Pandeleteius hilaris*-*Isodacrys* clade; state 2 convergently present in *Hadromeropsis brevicoma*-*Pandeleteius rotundicollis* and *Paululusus* (l: 3, ci: 0.66, ri: 0.83).

52. Legs, profemora, width relative to metafemora: (0) Subequal; (1) at least one fifth wider (Fig. 5B). See character 51 in Franz (2012), character 20 in Jansen & Franz (2015), character 66 in Lanteri & Del Río (2017) and character 46 in Marvaldi et al. (2018). State 0 convergently present in *Megalostylus albicans*-*Minyomerus*, *Isodrusus debilis* and the *Isodacrys crispum*-*I. obrienorum* clade; state 1 convergently present in the *Hadromeropsis brevicoma*-*Isodacrys guatemalenum* clade, the *I. mexicanum*-*I. ovipennis* clade and the *I. kuchii*-*I. fasciatum* clade (l: 5, ci: 0.20, ri: 0.71).

53. Legs, protibiae, length relative to metatibiae: (0) Subequal (Fig. 6A); (1) at least one fifth longer (Fig. 5B). State 0 convergently present in *Megalostylus albicans*-*Minyomerus*, *Pandeleteinus elytroplanatus*, *Isodrusus debilis* and the *Isodacrys crispum*-*I. obrienorum* clade; state 1 convergently present in the *Hadromeropsis brevicoma*-*Isodacrys guatemalenum* clade and the *I. mexicanum*-*I. ovipennis* clade (l: 5, ci: 0.20, ri: 0.78).

54. Legs, inner margin of protibiae, teeth: (0) Absent (Fig. 2); (1) present. See character 56 in Franz (2012), character 70 in Lanteri & Del Río (2017), character 31 in Del Río et al. (2018) and character 48 in Marvaldi et al. (2018). DELTRAN optimization is preferred based on the assumption of no immediate relationship with *Platyaspistes prasinus* (Leptopiini). Thus, state 1 convergently present in *P. prasinus*, the *Hadromeropsis brevicoma*-*Isodacrys ovipennis* clade and *I. antrum*, with subsequent reversals in *Pandeleteinus subcancer*, *Isodrusus debilis*, the *Isodacrys buchanani*-*I. schwarzi* clade and the *I. kuchii*-*I. obrienorum* clade (l: 7, ci: 0.14, ri: 0.57).

55. Legs, metatibial apex, vestiture of distal comb: (0) Bristles setiform, shorter or as long as surrounding setae (Fig. 6E); (1) bristles conical, thickened, shorter than surrounding setae. See character 22 in Jansen & Franz (2015). Character state 1 synapomorphy for *Minyomerus* (see figures 5 and 6 in Cortes-Hernández & Morrone, 2019; l: 1, ci: 1, ri: 1).

56. Legs, metatibial apex, corbel: (0) Present; (1) absent (Fig. 6D). See character 58 in Franz (2012), character 71 in Lanteri & Del Río (2017) and character 50 in Marvaldi et al. (2018). Character state 1 synapomorphy for the *Hadromeropsis*-*Isodacrys* clade (l: 1, ci: 1, ri: 1).

57. Legs, metatibial apex, false corbel: (0) Absent (Fig. 6D); (1) present. Coded as inapplicable when true corbel is present (see character 56, state 0). See character 58 in Franz (2012) and character 51 in Marvaldi et al. (2018). Character state 1 synapomorphy for *Scalaventer* (see figures 3 and 4 in Cortes-Hernández & Morrone, 2019; l: 1, ci: 1, ri: 1).

58. Legs, metatibial apex, distal comb, arrangement: (0) Straight to slightly arcuate, at most with bristles progressively longer posteriorly; (1) restricted to outermost region of tibiae; (2) obliquely arcuate, bristles in two discernible parts, the outermost bearing longer bristles (Fig. 6E). Non-additive. Character state 0 present in *Megalostylus albicans* and *Minyomerus*; character state 1 autapomorphy for *Platyaspistes prasinus*; character state 2 synapomorphy for the *Hadromeropsis brevicoma*-*Isodacrys* clade (l: 2, ci: 1, ri: 1).

59. Legs, tarsomeres 1, 2 and 3, ventral surface, vestiture: (0) With pads of setiform setae (Fig. 6E); (1) with stout, spiniform setae. See character 25 in Jansen & Franz (2015). Character state 1 synapomorphy for *Minyomerus* (l: 1, ci: 1, ri: 1).

60. Legs, tarsal claws, arrangement: (0) Free (Fig. 6E); (1) connate. See characters 61 and 53 in Franz (2012) and in Marvaldi et al. (2018), respectively. Character state 1 convergently present in *Platyaspistes prasinus* and the aff. *Isodrusus* sp.-*Isodrusus guajavus* clade (l: 2, ci: 0.50, ri: 0.66).

*Abdomen* (Fig. 6)

Howden (1969) observed that several genera of Neotropical Tanymecini have the anterior margin of ventrites III, IV and V modified, each with a transverse sulcus (Fig. 6F). The sulci of ventrites IV and V are each progressively wider. These sulci can be extended across the entire width of each ventrite or medially only (Anderson, 2002), enclosed by the lateral margins of the ventrites, where they are squamose (Howden, 1970). The posterior margin of the sulcus can be evenly rounded relative to the posterior surface of the ventrite or carinate. These sulci were highlighted as potentially valuable characters on a generic and a specific level in Tanymecini (Howden, 1969).

61. Abdomen, anterior margin of ventrites III, IV and V, sulcus: (0) Absent, ventrites flat; (1) present (Fig. 6F). Character state 1 synapomorphy for the *Pandeleteinus*-*Isodacrys* clade, with reversal in *Isodillex* (l: 2, ci: 0.50, ri: 0.88).

62. Abdomen, anterior margin of ventrites III, IV and V, degree of development: (0) Enclosed by lateral margins of ventrites (Fig. 6F); (1) along entire width. Coded as inapplicable when sulci are absent (see character 61, state 0). Character state 1 convergently present in the aff. *Isodrusus* sp.-*Isodrusus guajavus* clade and *Scalaventer* (l: 2, ci: 0.50, ri: 0.80).

63. Abdomen, anterior margin of ventrites III, IV and V, posterior margin of sulcus: (0) Present in all, sharply delimiting sulcus; (1) obliterate in ventrites IV and V, sulcus becoming flat posteriorly (Fig. 6F). Coded as inapplicable when sulci are absent (see character 61, state 0). State 0 convergently present in *Pandeleteinus subcancer*, the aff. *Isodrusus* sp.-*Isodrusus guajavus* clade, *Scalaventer* and *Isodacrys crispum*; state 1 present in the *Pandeleteinus*-*Isodacrys* clade (l: 4, ci: 0.25, ri: 0.57).

*Terminalia* (Figs. 3 and 7)

Characters of the female terminalia are explained and discussed in Howden (1995) and Lanteri & Del Río (2008). Particular character states for *Isodacrys* and other New World Tanymecini are herein highlighted. The ovipositor in *Isodacrys* is membranous and flexible, with slightly sclerotized regions, including the gonocoxites, baculi and proximal rods (Fig. 3F). The distal gonocoxites are usually moderately sclerotized laterally and the styli are reduced to a single thickened seta (Figs. 3F and 7B). Although Howden (1995) stated that *Pandeleteius hilaris* has a single ventral baculus, there are two baculi very close to each other, diverging posteriorly just before the distal gonocoxites. This condition is also present in *Isodacrys* species (Fig. 7B) as in other Neotropical tanymecines.

Although Howden pointed out the relevance of the aedeagus to separate some genera of Neotropical Tanymecini (Howden, 1961, 1970) she did not establish discrete characters or clear differences among aedeagus types. Following her observations, we consider the following aedeagal characters into account. The penis of some entimines has a sclerotized structure in the proximal portion of the internal sac within the median lobe, referred as genital sclerite (Bruhn, 1947; Oberprieler, 2014; Jansen & Franz, 2015, 2018). In *Isodacrys* the genital sclerite within the median lobe is absent, but a proximal flagellum can be present (Fig. 3E). In the pedal type of aedeagus, the sternite VIII is divided into two hemisternites (Oberprieler, Anderson & Marvaldi, 2014). These hemisternites are crescentic or teardrop shaped in the terminals included in this study, with their outer corners acute or truncate. Howden (1961) stated that the median lobe in *Isodacrys* is generally short and stout, but males remain unknown in eight species. The length/width ratio of the median lobe was considered in preliminary analyses, depicting a high level of incongruence (homoplasy). For that reason, it was excluded from subsequent analyses. Length relative to other genital structures resulted in more informative homology hypotheses.

64. Female terminalia, sternite VIII, lamina, shape: (0) Subrhomboidal; (1) subcordiform; (2) triangular (Fig. 7A). Non-additive. See character 78 in Lanteri & Del Río (2017). DELTRAN optimization is preferred, which establishes common origin of character state 1 in *Minyomerus* and not in *Platyaspistes prasinus* (Leptopiini), where genital characters were not observed. Thus, state 1 synapomorphy for *Minyomerus*; state 2 for the *Hadromeropsis brevicoma*-*Isodacrys* clade (l: 2, ci: 1, ri: 1).

65. Female terminalia, sternite VIII, apex of lamina, shape: (0) Acute (Fig. 7A); blunt (1). Coded as inapplicable when the lamina of sternite VIII is not triangular (see character 64, state 2). See character 79 in Lanteri & Del Río (2017). State 1 convergently present in *Hadromeropsis brevicoma*, the *Paululusus*-*Scalaventer* clade, *Isodillex*, *Isodacrys guatemalenum*, *I. okuiltontli*, *I. mexicanum* and the *I. geminatum*-*I. obrienorum* clade (l: 7, ci: 0.14, ri: 0.50).

66. Female terminalia, ovipositor, baculi, arrangement: (0) Distinctly separated by distance greater than width of one baculus; (1) almost contiguous, separated by distance similar to width of one baculus (Fig. 7B). Character state 0 present in *Megalostylus albicans*-*Minyomerus*; state 1 present in *Pandeleteius*-*Isodacrys* (l: 1, ci: 1, ri: 1).

67. Female terminalia, ovipositor, distal gonocoxites, degree of sclerotization: (0) Not sclerotized; (1) slightly to moderately sclerotized laterally (Fig. 7B); (2) strongly sclerotized. Non-additive. See character 84 in Lanteri & Del Río (2017). Character state 1 synapomorphy for the *Pandeleteius*-*Isodacrys* clade; character state 2 present in *Minyomerus*-*Hadromeropsis brevicoma* (l: 2, ci: 1, ri: 1).

68. Female terminalia, ovipositor, styli : (0) Present, strongly sclerotized; (1) vestigial, represented by single thickened seta (Fig. 7B). Character state 1 synapomorphy for the *Pandeleteius*-*Isodacrys* clade (l: 1, ci: 1, ri: 1).

69. Male terminalia, genital sclerite: (0) Present; (1) absent (Fig 7C). See character 37 in Jansen & Franz (2015) and character 99 in Lanteri & Del Río (2017). Character state 0 convergently present in *Megalostylus albicans*-*Pandeleteius rotundicollis*, *Paradacrys ensiformis*, *Scalaventer jamaicensis* and *S. cyrillae*; character state 1 present in the *Pandeleteius hilaris*-*Isodacrys* clade (l: 4, ci: 0.25, ri: 0.40).

70. Male terminalia, sternite VIII, outer corner, shape: (0) Truncate; (1) not truncate (Fig. 7D). ACCTRAN optimization preferred because it posits a common origin of character state 1. Thus, character state 0 convergently present in *Megalostylus albicans*, *Hadromeropsis brevicoma*, *Pandeleteius hilaris* and *Isodacrys apicale*-*I. ovipennis* clade; character state 1 synapomorphy for the *Pandeleteius*-*Isodacrys* clade (l: 3, ci: 0.33, ri: 0.66).

71. Male terminalia, median lobe, length relative to spiculum gastrale: (0) About as long as spiculum gastrale; (1) shorter. DELTRAN optimization is preferred based on the absence of males in *Isodacrys ellipticum*, therefore character state 1 is considered as independent in *I. guatemalenum*. Thus, state 1 convergently present in aff. *Pandeleteinus* sp., *Paradacrys ensiformis*, *Isodacrys guatemalenum*, the *I. orizabae*-*I. ovipennis* clade and *I. kuchii* (l: 6, ci: 0.16, ri: 0.28).

72. Male terminalia, tegmen, manubrium, length relative to median lobe: (0) Shorter; (1) as long as median lobe; (2) longer (Fig. 7C). Non-additive. State 0 convergently present in *Megalostylus albicans*-*Pandeleteius hilaris* and the aff. *Pandeleteinus* sp.-*Isodillex plumosum* clade; state 1 synapomorphy for *Isodacrys* (although with an evolutionary transition to state 2 in the *I. kuchii*-*I. obrienorum* clade); state 2 convergently present in *Pandeleteinus* and the *Isodacrys kuchii*-*I. obrienorum* clade (l: 5, ci: 0.40, ri: 0.66).

**Systematic revision of the genus *Isodacrys*** Sharp, 1911

(Figs. 1, 2, 3, 4B, 4D, 4E, 5A, 5C–5F, 6–25)

*Isodacrys* Sharp, 1911: 175–177; Champion, 1911: 341. Gender masculine.

Type species. *Isodacrys guatemalenum* Sharp, 1911; subsequently designated by Pierce, 1913: 401.

**Diagnosis.** ♂ 1.7–3 mm long, 0.7–1.1 wide, ♀ 1.9–3.7 mm long, 0.9–1.6 mm wide. Dorsal scales variously overlapping non-linearly or contiguous, polygonal (chars.1:0,1 and 2:1). Dorsolateral margins of rostrum subparallel (char. 3:1), dorsal surface flat, nasal plate with anterior margin medially indented (chars. 4:0 and 5:1), posterior margin carinate (char. 6:1); epistome in continuous plane with remainder of rostrum (chars. 7:1 and 8:2), or slightly depressed, covered by smaller, shinier scales of different shape than remainder of rostrum, posterior margin of epistome indistinct (char. 9:0); scrobes glabrous (char. 14:1), angled, from obtusely-angled to right-angled (char. 15:2,3), reaching ventral surface of rostrum (char. 16:1); head deflexed, directed ventrally (char. 19:1); mandibles covered with setae, without scales (char. 22:1); prementum incompletely concealing maxillae (char. 23:1); antennal scape capitate, abruptly thickened at apical region (char. 26:2), funicular antennomere I longer than funicular antennomere II (char. 27:2); pronotum in dorsal view subcylindrical (char. 28:2), from as wide as long to longer than wide (char. 29:1,2), with constrictions near anterior and posterior margins (char. 30:1), anterolateral margin of prothorax with postocular vibrissae or not (char. 32:0,1), when present usually vestigial (very short and thin) or with less than five well developed postocular vibrissae (char. 34:1,2), intercoxal process of prosternum continuous, separating procoxae (char. 35:1); humeri absent (char. 39:1), basal margin of elytra roundly or angularly emarginate (chars. 41:2 and 42:2); profemora as long as metafemora (char. 51:1), wider or not (char. 52:0,1), protibiae longer than metatibiae or not (char. 53:0,1), apex of metatibiae simple (chars. 56:1 and 57:0), distal comb of metatibial apex obliquely arquate (char. 58:2), tarsal claws free (char. 60:0); anterior margins of ventrites III, IV and V sulcate, sulci enclosed by lateral margins of ventrites (chars. 61:1 and 62:0); manubrium usually as long as or longer than median lobe, rarely shorter (char. 72:0,1,2).

The genus *Isodacrys* can be confused with other wingless tanymecines (*Minyomerus*, *Isodillex*, *Paululusus* in part). *Isodacrys* is easily distinguished from *Minyomerus* by the metatibial apex simple, covered by setae, whereas *Minyomerus* possesses a true corbel, covered by conical thickened bristles. *Isodacrys* can be separated from *Paululusus* by the scrobe reaching the ventral surface of the rostrum and the profemora as long as the metafemora, whereas in *Paululusus* the scrobe ends before the ventral surface of the rostrum and the profemora are longer than the metafemora. Finally, *Isodacrys* is easily separated from *Isodillex* by the sulcate anterior margins of ventrites III, IV and V, whereas in *Isodillex* the anterior margins of ventrites III, IV and V are flat, not sulcate.

**Figure 8:**
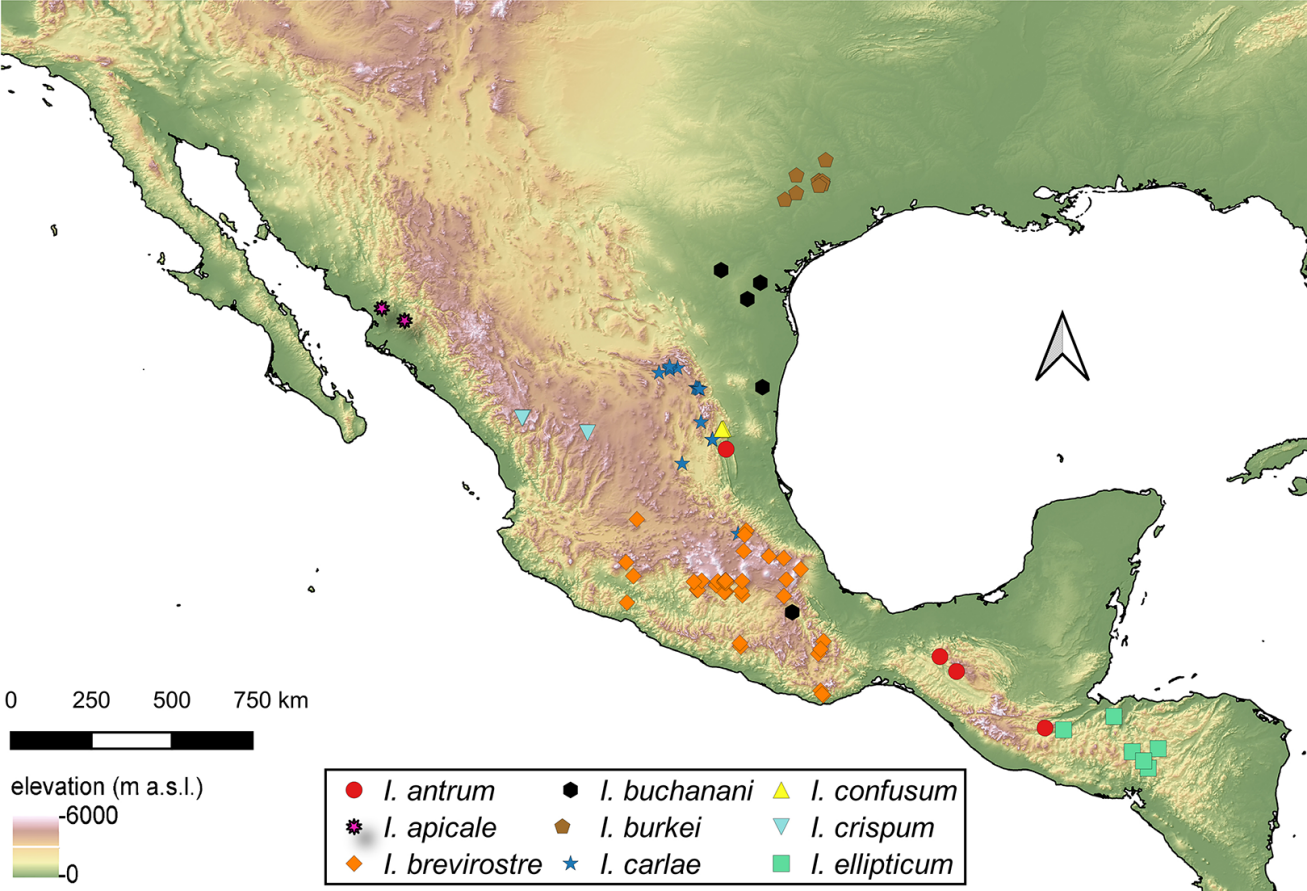
Occurrence map of nine *Isodacrys* species, ordered alphabetically.

**Figure 9:**
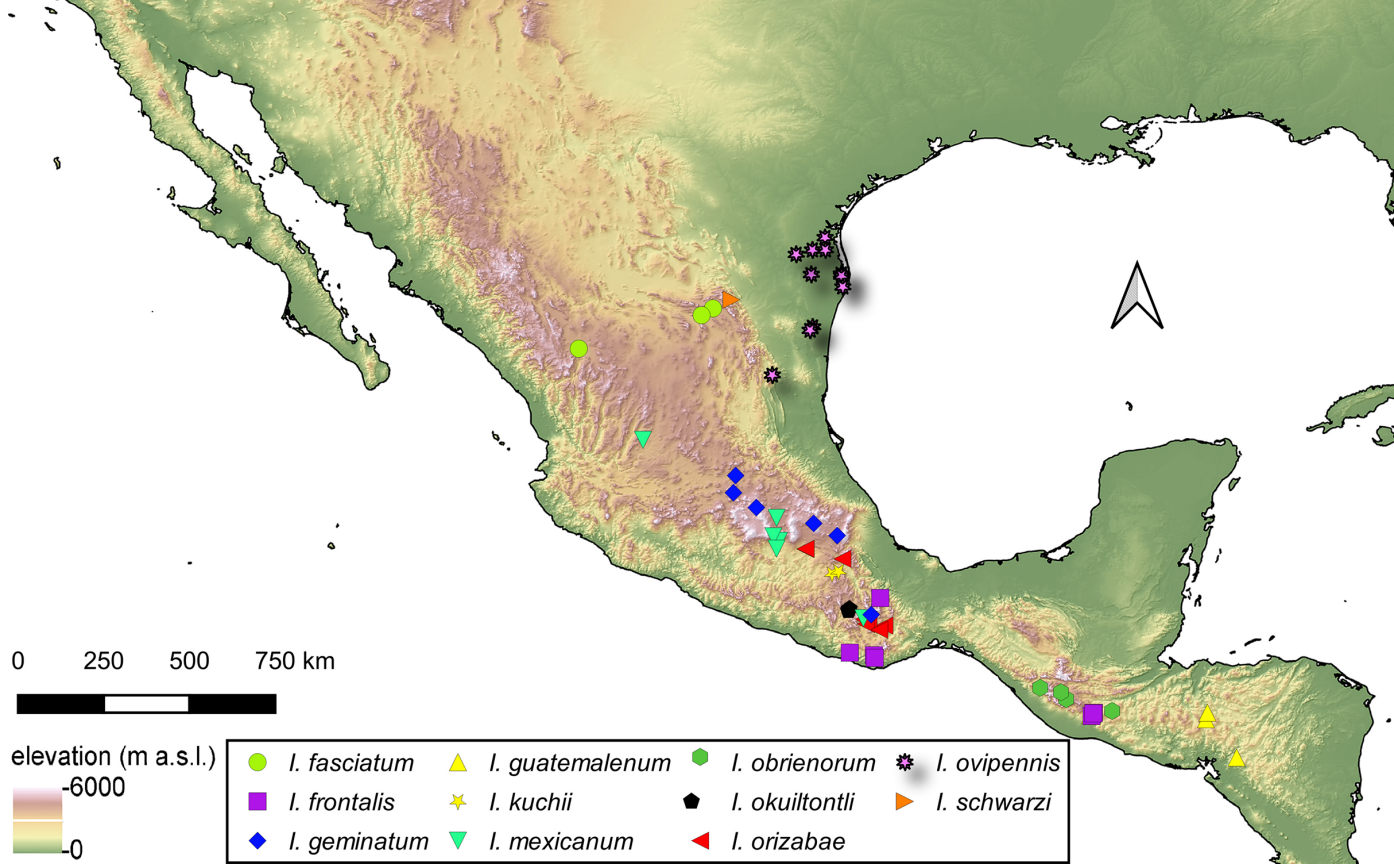
Occurrence map of the remaining eleven *Isodacrys* species, ordered alphabetically.

**Remarks.** *Isodacrys* species are distributed from south of the United States of America to Honduras (Figs. 8–9), mainly across Mexican and Central American mountain ranges of ~1500–3100 m above sea level; some species also occur in lowlands. Adults have been found on nine plant families (Asteraceae, Betulaceae, Cucurbitaceae, Fabaceae, Fagaceae, Malvaceae, Pinaceae, Rhamnaceae and Solanaceae), leaf litter, unidentified mushrooms and under rocks.

***Isodacrys antrum* Cortés-Hernández, new species**

(Figs. 8, 10–11)

**Figure 10:**
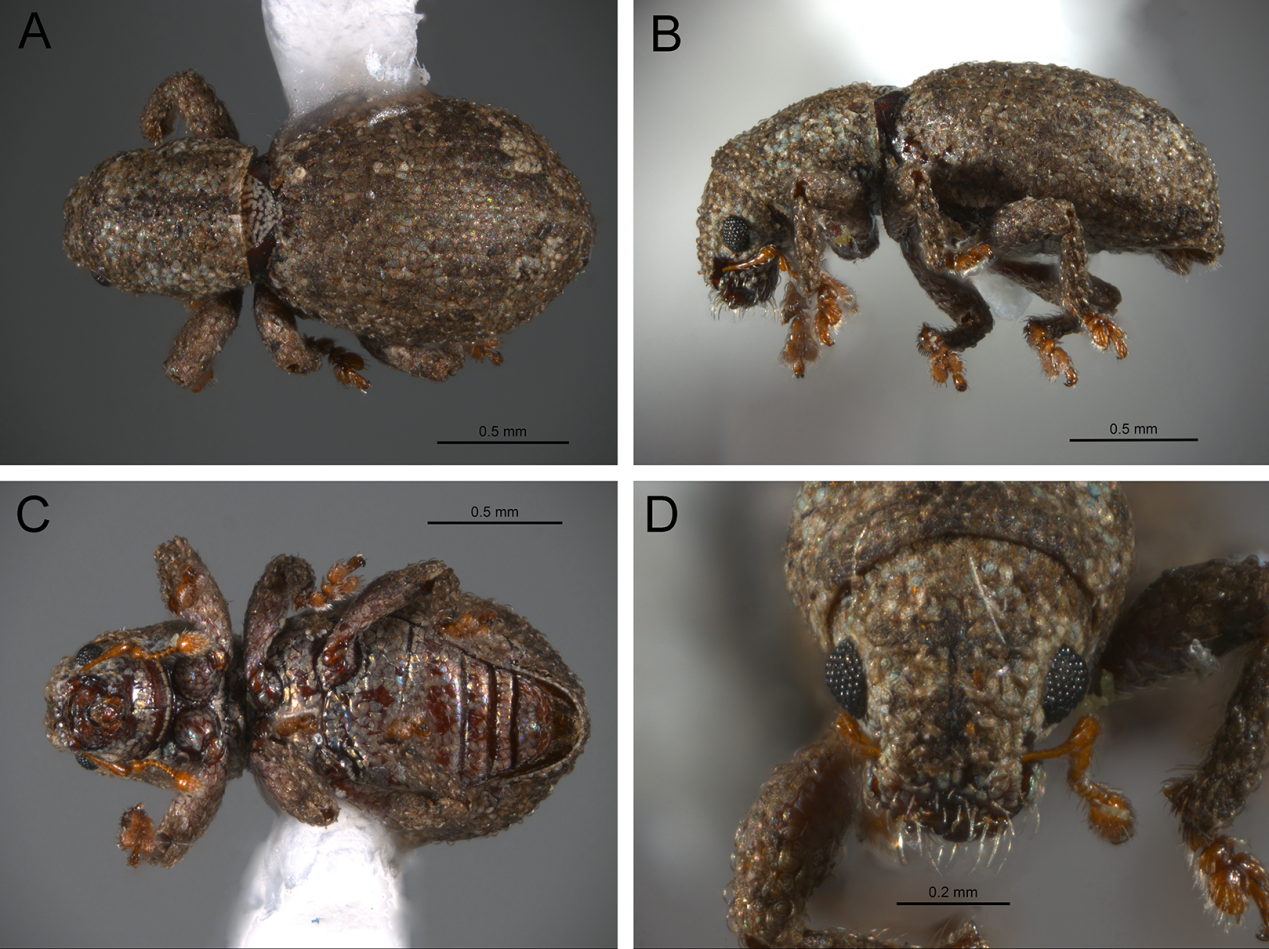
*Isodacrys antrum* new species. (A) Dorsal habitus; (B) lateral habitus; (C) ventral habitus; (D) head in anterior view.

**Figure 11:**
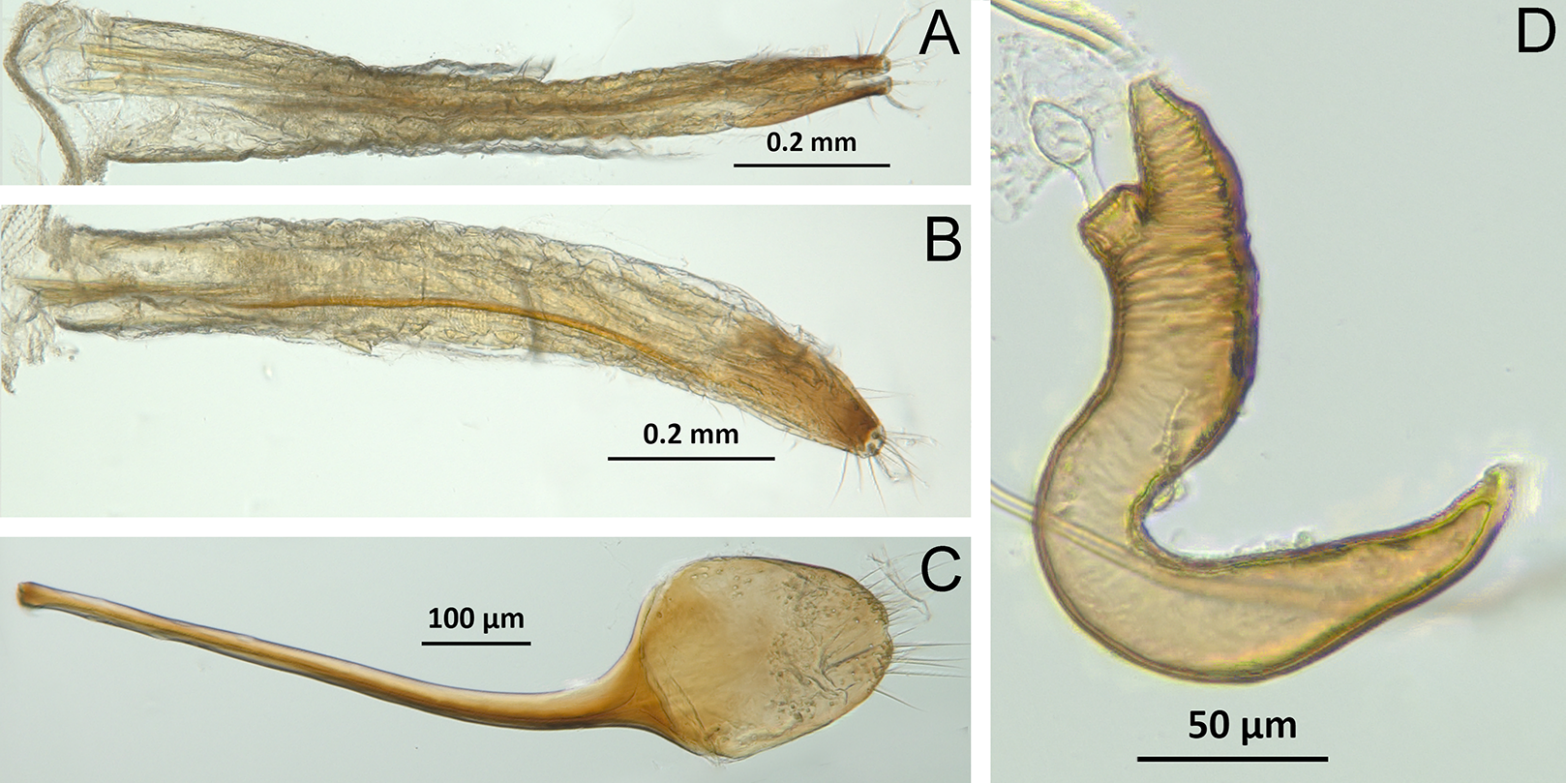
*Isodacrys antrum* new species, female genitalia. (A) Ovipositor in dorsal view; (B) ovipositor in lateral view; (C) sternite VIII in dorsal view; (D) spermatheca.

urn:lsid:zoobank.org:act:FBE7F34A-9C93-4BDC-9423-3D3A915B926A

**Diagnosis.** Setae lanceolate, completely arched, mostly inconspicuous on temple and disc of pronotum; scrobe with dorsal margin right-angled, posterior portion of scrobe separated from eye by distance shorter than width of scrobe; head in lateral view with dorsal outline continuously curved towards rostrum (Fig. 10B); anterolateral margin of prothorax without postocular vibrissae (Fig. 10B); elytra with surface of intervals seven to nine strongly concave near base (Fig. 10B); protibiae slightly bent inward at apical fourth, inner margin with three small teeth; lamina of female sternite VIII oval (Fig. 11C).

*Isodacrys antrum* can be confused with *I. obrienorum*, *I. geminatum*, *I. brevirostre* and *I. frontalis. Isodacrys antrum* can be readily separated from *I. obrienorum* and *I. geminatum* by the concavity near the base of the elytral intervals seven to nine and the protibiae with three small teeth along the inner margin. Neither *Isodacrys obrienorum* nor *I. geminatum* present any concavity near the base of the elytra and the inner margins of the protibiae are smooth, without teeth. In *I. brevirostre* and *I. frontalis* the concavity near base of the elytral intervals seven to nine is shallower and therefore not as conspicuous as in *I. antrum*. Additionally, in *Isodacrys brevirostre* and *I. frontalis* the inner margins of the protibiae are smooth, without teeth.

**Description – Habitus.** ♀ 1.9–2.1 mm long, 0.8–1.0 mm wide. Scales polygonal, granulate, mainly overlapping irregularly, from dark brown to cinereous in coloration. Head and rostrum with irregular pattern of brown and cinereous scales; scales surrounding eyes lighter, cinereous. Disc of pronotum with brown scales, with narrow median longitudinal vitta of dark brown scales, conspicuous or not; sides of prothorax with irregular, broad, longitudinal vittae of cinereous scales. Base of elytral intervals three to seven with cinereous scales, disc of elytra with irregular pattern of brown and cinereous scales. Setae lanceolate, completely arched, mostly inconspicuous on temple and disc of pronotum. **Rostrum.** Short, dorsolateral margins of rostrum subparallel, with inconspicuous longitudinal sulci mesad of dorsolateral margins at bent of scrobes (Fig. 10D); dorsal surface flat; median sulcus indistinct. Nasal plate with surface shiny, rugulose; anterior margin medially indented; posterior margin V-shaped, carinate. Epistome in continuous plane with remainder of rostrum, covered by two or three rows of shiny, small scales (Fig. 10D); anterior margin bearing six epistomal setae on each side; posterior margin fuzzy, distinguishable by presence of greater, not modified scales. Scrobe deep, bent, reaching ventral surface of rostrum; dorsal margin right-angled; anterior portion of scrobe about 2/3 length of posterior portion; posterior portion of scrobe separated from eye by distance shorter than width of scrobe (Fig. 10B). Antenna setose, without scales; scape capitate (Fig. 10D); funicular antennomere I clavate, wider and longer than remaining antennomeres; funicular antennomere II clavate, longer than remaining funicular antennomeres; funicular antennomeres III to VII moniliform, distal antennomeres becoming broader. **Head.** In lateral view with dorsal outline continuously curved to rostrum, frons not prominent(Fig. 10B); eyes moderate in size, suboval, prominent, lateral, separated from outline of frons by 1/2–2/3 diameter of eye. **Prothorax.** Pronotum in dorsal view subcylindrical, as wide as long, lateral outlines sinuate; in lateral view dorsal outline almost straight, anterior and posterior constrictions inconspicuous. Anterolateral margin of prothorax without postocular vibrissae or scales projected anteriorly (Fig. 10B). **Elytra.** 1.9–2.1 times longer than pronotum, at base as wide as base of prothorax. Basal margin of elytra roundly emarginate. Elytra in dorsal view subelliptical, widest at midlength (Fig. 10B), five intervals visible at base. Elytra in lateral view with dorsal outline evenly curved towards summit of apical declivity; apical declivity oblique. Surface of intervals seven to nine strongly concave near base (Fig. 10B). **Legs.** Procoxae narrowly separated (Fig. 10C), intercoxal process 1/7–1/6 width of procoxa; profemora fusiform, subequal in length and width to metafemora; protibiae slightly bent inward at apical fourth, as long as metatibiae, with three small teeth along inner margin. Dorsal surface of tarsi setose, first tarsomere with few appressed scales or not (probably lost by abrasion). Tarsal claws free. **Abdomen.** Ventrites III, IV and V with anterior margin narrowly sulcate, progressively wider and deeper (Fig. 10C); sulci enclosed by lateral margins of ventrites; posterior margin of sulci obliterated, ventrites becoming flat posteriorly. **Genitalia.** ♀: Lamina of sternite VIII oval (Fig. 11C); spiculum ventrale 2.0–2.2 times length of lamina. Ovipositor with distal gonocoxites slightly sclerotized laterally, styli represented by single thickened seta; two ventral baculi close to each other, separated by distance similar to width of one baculus; vagina with two pairs of lightly sclerotized proximal rods. Spermatheca u-shaped; corpus slightly wide; collum produced, conical; ramus broadly cylindrical, short; cornu strongly curved near corpus, then gently sinuate, elongate, reaching apex of ramus, apically gradually narrowed (Fig. 11D).

**Type material.** Holotype ♀: [MEXICO: Tamaulipas/El Cielo, 3 km SE Alta Cima/23.03440–99.186967 ± 20 m/860 m, 24.VIII.2009/L.D. Sáenz LSD-370/cloud forest, nest under rock] [WORLD/WEEVIL/DATABASE/WWD0133374] [*Isodrusus*/or/*Isodacrys*/det. R.S. Anderson, 20] (CMNC). Paratypes (5 ♀♀): [GUAT.: BAJA VERAPAZ/8.6 km.W. Chilascó, 1,560 m./24.V.1991, R. Anderson/oak|pine|liquidambar/forest litter, 91–18] (2, CMNC). [MEXICO: Chiapas/Mpio: Tapalapa/12 Km N Coapilla/12-III-94, R. Jones] [*Isodacrys*/Det. R.S. Anderson, 1995] (1, CMNC); [MEX.: CHIAPAS, 3 km./W. San Cristobal, 2,000 m./18.IX.1991, R. Anderson/oak|pine forest, 91–108] (1, MZFC); [MEXICO: Tamaulipas/same data as holotype] [WORLD/WEEVIL/DATABASE/WWD0133381] (1, CMNC).

**Etymology.** From the Latin noun *antrum*, meaning cavity, referring to the concavities on intervals seven to nine, stronger and more conspicuous than elytral depressions in other *Isodacrys* species.

**Remarks.** Adults have been collected in an ant nest under a rock and in leaf litter, in cloud and oak-pine forests, between 860 and 2,000 m above sea level. Males remain unknown.

**Comments.** The shape of the lamina of female sternite VIII is uniquely shaped among *Isodacrys* species.

***Isodacrys apicale* Howden, 1961**

(Figs. 3B, 4E, 5C, 6B, 8)

*Isodacrys apicale* Howden, 1961: 91–92. Holotype: ♂, examined; labeled as [151|35/Choix, SIN./13-VIII-35] [HOLOTYPE/*Isodacrys*/*apicale*/A.T.Howden/No.7385] [CNC/379712]. O’Brien & Wibmer, 1982: 46 (checklist); Morrone, 1999: 145 (checklist); Cortes-Hernández & Morrone, 2019: 50.

**Diagnosis.** Setae of elytra spatulate, incompletely arched, erect on declivity; scrobe with anterior portion as long as posterior portion, posterior portion separated from eye by distance 1.2 times width of scrobe (Fig. 4E); eyes large, subcircular; pronotum as wide as long, anterolateral margin of prothorax with more than six postocular vibrissae, some well-developed (Fig. 4E); elytra in dorsal view obovate, widest before midlength, with seven intervals visible at base, apical margins of elytra bisinuate (Fig. 6B); protibiae with teeth along inner margin, prolegs not larger than metalegs; median lobe stout, with apex in dorsal view attenuated into acute point.

*Isodacrys apicale* can be readily separated from *I. mexicanum*, *I. orizabae*, *I. burkei* and *I. ovipennis* by the prothorax with well-developed postocular vibrissae, the prolegs not larger than the metalegs and apical margins of the elytra bisinuate. In the other mentioned species, the postocular vibrissae are reduced in number and usually reduced in size too (or absent as in *I. burkei*), the prolegs are larger than the metalegs and the apical margins of the elytra are conjointly rounded, not bisinuate.

**Additional material examined.** MEXICO: Sinaloa, same data as holotype (1 paratype, CMNC); Sonora, 1 mi. W Álamos, 16.VII.1964, H.R. Burke, J. Apperson (1, TAMUIC).

**Remarks.** The apex of ventrite V in the male is emarginate, whereas in the females, the apex of ventrite V is rounded (see also Howden, 1961).

**Comments.** *Isodacrys apicale* was described from a series of three specimens from Choix, Sinaloa, Mexico. The holotype, deposited in CNC, was examined. One paratype is deposited in CMNC and another paratype is deposited in Dirección General de Defensa Agrícola (SAGARPA, Mexico; Howden, 1961). The examined specimen from Sonora agrees with both the types examined and the original species description.

Photographs of the holotype were examined to complement the description of the male genitalia in diagnosis. Female genitalia were not observed.

***Isodacrys brevirostre* Howden, 1961**

(Figs. 1, 3A, 4D, 5A, 8)

*Isodacrys brevirostre* Howden, 1961: 79–80. Holotype: ♀, examined; labeled as [Cuernavaca 6 Mi.,/N., 7500′ Morelos/15-VIII-1954/J. G. Chillcott] [HOLOTYPE/*Isodacrys*/*brevirostre*/A.T.Howden/No. 7383] [CNC/379713]. O’Brien & Wibmer, 1982: 46 (checklist); Morrone, 1999: 145 (checklist); Cortes-Hernández & Morrone, 2019: 50.

**Diagnosis.** Setae of elytra spatulate, semierect, becoming erect towards apex of elytra; rostrum short, with longitudinal sulci mesad of dorsolateral margins at bent of scrobes (Fig. 4D); scrobe with anterior portion as long as posterior portion, posterior portion separated from eye by distance equal to width of scrobe; anterolateral margin of prothorax without postocular vibrissae; elytra in dorsal view subelliptical, widest at midlength, with five intervals visible at base, intervals seven to nine slightly depressed at base; prolegs not larger than metalegs, inner margin of protibiae without teeth; spermatheca fishhook-shaped, corpus wide, collum produced, subcylindrical, bent apically towards ramus, ramus inconspicuous.

*Isodacrys brevirostre* can be confused with other species with non-denticulate protibiae: *Isodacrys frontalis*, *I. geminatum* and *I. obrienorum*. *Isodacrys brevirostre* can be easily separated from *I. frontalis* by its spatulate and semierect elytral setae, becoming erect towards the apex of the elytra, the frons not tumescent and posterior portion of the scrobe separated from eye by distance equal to width of the scrobe. In *I. frontalis*, elytral setae are lanceolate, from completely arched to incompletely arched, the frons strongly prominent and posterior portion of the scrobe separated from eye by at least twice width of the scrobe. From *Isodacrys geminatum* and *I. obrienorum, I. brevirostre* can be distinguished also by the elytral setae, which are lanceolate in *I. geminatum* and *I. obrienorum*, and the right-angled scrobe (obtusely angled in *I. brevirostre*), with posterior portion of the scrobe separated from eye by a distance shorter than the width of the scrobe. Finally, it can be also confused with *I. antrum*, which possesses teeth along the inner margin of the protibiae and the head in lateral view with dorsal outline continuously curved towards the rostrum. In *I. brevirostre* inner margin of the protibiae is smooth, without teeth, and the dorsal outline of the head is at least slightly deflexed at the frons towards the rostrum.

**Additional material examined.** MEXICO: Estado de México, 6 km al SE de Atlautla, 27.IX.2017, Redeo en vegetación, B. Pino-Encino, 2450, 19°00′46" N, 98°45′26″ W, K. Cortés, L. Delgado (1, IEXA); Estado de México, La Mirasol, 7 km SW Santiago de Tianguistengo, 2.XI.1973, 2,800 m, C.W. O’Brien (36, ASUCOB; 3, CASENT); Estado de México, Ocoyoacac, 28.X.1973, on misc. flowers, C.W. O’Brien (4, CASENT; 89, ASUCOB); Estado de México, Bejucos, Mex. Temascaltepec, 3.VII.1933, H.E. Hinton, R.L. Usinger (3 paratypes, CASENT); Estado de México, Real de Arriba, VII.1932, H. Hinton (1 paratype, TAMUIC); Estado de México, Hwy 190, 11 mi. W Río Frío, 5.VI.1983, 9800′, C. & L. O’Brien, G. Marshall (3, ASUCOB); Estado de México, Hwy15, 28 mi. W. Toluca, 6.VIII.1982, 9100′, L. O’Brien, G. Wibmer (38, ASUCOB); Guanajuato, 3.6 mi. NE Guanajuato, 5.VII.1985, Woolley, Zolnerowich (1, TAMUIC); Guerrero, 6 km NE Ayotoxtla (Tlatlauquitepec Rd), 24.X.1973, A.N. García A. (2, ASUCOB); Guerrero, 9 km SE Tlatlauquitepec, 24.X.1974, A.N. García A. (1, ASUCOB); Hidalgo, Hwy105 16 mi. N Metzquititlán, 30.VII.1982, 7200′, L. O’Brien, G. Wibmer (23, ASUCOB); Hidalgo, Hwy105 Mineral Real del Monte, 14.VI.1983, 9400′, C. & L. O’Brien, G. Marshall (1, ASUCOB); Hidalgo, Hwy105, 3 mi. S Zacualtipan, 3.VIII.1982, 6959′, C. & L. O’Brien, G. Wibmer (2, ASUCOB); Mexico City, Cañada Contreras, 5.X.1982, s/composit, 2,550 m, K. Luna (10, MZFC); Mexico City, Road from Mexico City P.N. Cumbres de Ajusco, 4.IX.1982, 2, 750 m, C. & L. O’Brien, G. Wibmer (17, ASUCOB); Mexico City, Delegación Tlalpan, Tlalpuente, 4.XI.1993, L. Torres-Miller (1, CMNC); Mexico City, El Pedregal, 4.IX.1982, 2,340 m, C. & L. O’Brien, G. Wibmer (1, ASUCOB); Mexico City, Unión Perif. Ajusco, 4.IX.1982, 2,340 m, K. Luna (1, MZFC); Mexico City, 7 mi. N Valle de Bravo, 7.VIII.1982, 8300′, C. & L. O’Brien, G. Wibmer (1, ASUCOB); Michoacán, 2 mi. S Carapan, 6.VII.1986, H. & A. Howden (1, CMNC); Michoacán, 2 mi. S Carapan, 6.VII.1985, Woolley, Zolnerowich (1, TAMUIC); Michoacán, 28.5 mi. S Nueva Italia, 9.VII.1985, Woolley, Zolnerowich (1, TAMUIC); Michoacán, 17 mi. W Pátzcuaro, 4.VIII.1982, 7700′, C. & L. O’Brien, G. Wibmer (6, ASUCOB); Morelos, 8 km N Cuernavaca, Hwy 95, 5.IX.1982, C. & L. O’Brien, G. Wibmer (5, ASUCOB); Morelos, Santa María Ahuacatitlán, 6.XI.1980, F. Aguirre (1, CNIN); Morelos, Tetela del Volcán, 23.VIII.1984, V. Butze (1, CNIN); Oaxaca, Hwy 175, 27 km NE Oaxaca, 29.VIII.1982, 8500′, C. & L. O’Brien, G. Wibmer (1, ASUCOB); Oaxaca, Hwy 175, 66 km NE Oaxaca, 29.VIII.1982, 8500′, C. & L. O’Brien, G. Wibmer (1, ASUCOB); Oaxaca, Carr. Oaxaca-Tuxtepex, bosque de pino-encino, en hojarasca, 2,296 m, 17°20′57.8″ N, 96°30′57.5″ W, 9.VI.2018, M. Barrios (6, MZFC); Oaxaca, 2 mi. north San José Pacífico, 16.VII.1974, Clark, Murray, Ashe, Schaffner (4, CMNC); Oaxaca, 2 miles north San José Pacífico, 17.VII.1974, Clark, Murray, Ashe, Schaffner (2, TAMUIC); Oaxaca, 8 km S Suchixtepec, Río Molino, 19.VI.1979, 2,200 m, H. & A. Howden (30, CMNC; 2, ASUCOB); Oaxaca 8 km S Suchixtepec, Río Molino, 6.VIII.1986, H. & A. Howden (1, CMNC); Oaxaca, 10 km S Suchixtepec, 24.VII.1992, Roadside beating, 2,000 m, R.S. Anderson (24, CMNC); Oaxaca, 1 mi. S Suchixtepec Hwy 175, 2.VI.1983, 9200′, C. & L. O’Brien, G. Marshall (2, ASUCOB); Puebla, 67 mi. E Puebla, 26.VI.1971, 7800′, C.W. & L. O’Brien, Marshall (1, CASENT); Puebla, 7 mi. N Zacapoaxtla, 12.VI.1983, 5000′, C. & L. O’Brien, G. Marshall (1, ASUCOB); Puebla, 6 mi. NE Zacatepec, 27.VI.1975, sifting leaf litter, D.S. Chanddler (1, ASUCOB); Puebla, 6 mi. NE Zacatepec, 27.VI.1975, L.E. Watrous (1, ASUCOB); Puebla, Zacatlán km 2 desv. a Piedras Encimadas, 6.VII.2006, 2,449 m, 20°01′07″ N, 98°04′43″ W, L. Cervantes, D. Brzoska (1, CNIN); Veracruz, Hwy 140, 17 mi. NW Jalapa, 20.VIII.1982, 8000′, C. & L. O’Brien, G. Wibmer (1, ASUCOB).

**Remarks.** Adults of *I. brevirostre* have been found on *Condalia* sp. (Rhamnaceae; Howden, 1961), on Asteraceae, beating miscellaneous vegetation and in leaf litter, between ~2,000 and 2,800 m above sea level. Some were collected on pine-oak forests. Males remain unknown.

**Comments.** *Isodacrys brevirostre* was described based on 23 female specimens from several localities in central Mexico: Cuernavaca, Morelos; Toluca and Temascaltepec (misspelled as Temescaltepec), Estado de México; and Jacala, Hidalgo. The holotype, deposited in CNC, was examined. The 22 paratypes were originally placed in CASENT, CNC, Howdens’ collection (now housed at the Canadian Museum of Nature), and USNM. Three paratypes from CASENT and one from TAMUIC were also examined. The TAMUIC paratype is probably a subsequent donation from Howden collection.

Among the specimens observed, there are at least three character states that vary intraspecifically: length of the cornu of spermatheca, arrangement of dorsal scales (recorded as polymorphic in character 1) and scales color (from light brown to dark brown; see also Howden, 1961). Regardless of this intraspecific variation, we were not able to establish a clear-cut division among these morphotypes and treat them as different terminals in the phylogenetic analyses.

***Isodacrys buchanani* Howden, 1961**

(Figs. 2, 6F, 8)

*Isodacrys buchanani* Howden, 1961: 82–83. Holotype: ♂, not examined. O’Brien & Wibmer, 1982: 46 (checklist); Howden, 1993a: 2 (checklist); Morrone, 1999: 145 (checklist); Cortes-Hernández & Morrone, 2019: 50.

**Diagnosis.** Setae lanceolate, small, completely arched; scrobe with anterior portion at least one fifth shorter than posterior portion, posterior portion separated from eye by distance equal to width of scrobe; antennal scape covered with scales at apex dorsally; anterolateral margin of prothorax with one or two postocular vibrissae; basal margin of elytra angularly emarginate, sutural interval at summit of declivity strongly tumid, apical declivity strongly concave; prolegs not larger than metalegs, inner margin of protibiae without teeth; median lobe slender, as long as spiculum gastrale, nearly two times longer than temones, apex in dorsal view acute, manubrium as long as median lobe; spermatheca comma-shaped, corpus slightly subglobose, cornu and ramus inconspicuous.

*Isodacrys buchanani* can be readily separated from *I. schwarzi* by the prothorax with one or two reduced postocular vibrissae, which in the latter the postocular vibrissae are well developed, larger and more numerous.

**Material examined.** MEXICO: Puebla, 6 mi. SE Tehuacán, 7.VII.1973, Mastro, Schaffner (2, TAMUIC; 1, CMNC); Tamaulipas, 4 mi. S San Fernando, Hwy 101, 5.V.1983, C. & L. O’Brien, G. Marshall (1, ASUCOB). UNITED STATES OF AMERICA: Texas, Duval Co., 1 mi. W San Diego, 10.IV.1973, W.E. Clark (1, TAMUIC); Texas, 15 mi. SW Jct FR 3073 y Hwy 16, 10.IV.1987, B.F. & J.L. Carr (1, CMNC; 2, CNC); Texas, Jim Hogg Co., 5 mi. W Hebbronville, 3.XI.1990, T. Carlow (3, TAMUIC); Texas, 37 mi. N Laredo, Webb Co., 15.X.1970, C.W. O’Brien (53, ASUCOB; 6, CMNC).

**Remarks.** The holotype and the paratype were collected from cut flowers (Howden, 1961).

**Comments.** *Isodacrys buchanani* was described based on two specimens from Mexico, intercepted at Laredo, Texas, United States. As data in Howden (1961) indicate, since cut flowers are a relatively perishable commodity, it suggests that type locality is somewhere near Laredo in Tamaulipas or Nuevo León, Mexico. The holotype and the paratype are deposited in USNM and were not examined. Identity of material examined was corroborated from specimens identified by Anne Howden and by agreement the with original species description. Material examined confirm the occurrence of *I. buchanani* in Tamaulipas and also in Texas. Its southernmost occurrence is in Tehuacán, Puebla, México.

***Isodacrys burkei* Howden, 1961**

(Fig. 8)

*Isodacrys burkei* Howden, 1961: 88–90. Holotype: ♂, not examined. O’Brien & Wibmer, 1982: 46 (checklist); Howden, 1993a: 2 (checklist); Anderson, 2002: 780.

**Diagnosis.** Setae lanceolate, small, completely arched, mostly inconspicuous on disc of pronotum; epistome with longitudinal carina joined with posterior margin of nasal plate (e.g., Fig. 4C); scrobe with anterior portion at least one fifth shorter than posterior portion, posterior portion separated from eye by distance nearly twice width of scrobe; antennal scape covered with scales at apex dorsally; eyes large, slightly convex, not prominent; anterolateral margin of prothorax without postocular vibrissae, with conspicuous tooth extending anteriorly to eyes; elytra in dorsal view ovate, widest after midlength, intervals two and ten, and intervals five and six tumescent at their apical junctions, intervals three to five slightly elevated at base; profemora wider than metafemora, protibiae longer than metatibiae, inner margin of protibiae with teeth; median lobe shorter than spiculum gastrale, slightly longer than temones, apex in dorsal view acute, manubrium about as long as median lobe; spermatheca comma-shaped, corpus subglobose, collum produced, subconical, curved towards ramus, ramus broadly subcylindrical, short.

*Isodacrys burkei* can be readily separated from *I. ovipennis*, *I. orizabae* and *I. mexicanum* by anterolateral margin of the prothorax without postocular vibrissae, instead with a conspicuous tooth extending anteriorly towards the eyes. In *I. ovipennis*, *I. orizabae* and *I. mexicanum* the prothorax bears few postocular vibrissae and the anterolateral tooth of the prothorax is absent.

**Material examined.** UNITED STATES OF AMERICA: Texas, Brazos Co., 8.V.1956, H. R. Burke (1 paratype, TAMUIC); Texas, Brazos Co., 12.V.1960, H. R. Burke (1 paratype, TAMUIC); Texas, Brazos Co. Bastrop St. Pk., 24–27.V.1963, M. Kaulbars (2, CMNC); Texas, Brazos Co. Bastrop St. Pk., 5.VI.1989, Sweeping composites, R.S. Anderson (6, CMNC); Texas, Brazos Co., College Station, Lick Creek Park, 12.VI.1993, R. Jones (2, UAQE); Texas, College Station, Brazos Co., 15.IV.1963, H.R. Burke (20, CMNC); Texas, College Station, 3.V.1931, swept from weeds, H.J. Reinhard (1 paratype, TAMUIC); Texas, Brazos Co., on Hwy30 7.6 miles east jct. Hwy. 6, 7.V.1981, Sweeping *Coreopsis sp*., S. J. Merritt (1, TAMUIC); Texas, Brazos Co., Lick Ck. Pk., ca.3 mi. S College Station, 18.X.1987, R.S. Anderson (4, CASENT; 50, CMNC); Texas, Brazos Co. Lick Creek Park ca. 5 mi. S College Station, 16.IV.1989, Sweeping flowers, R.S. Anderson (2, CMNC); Texas, Lee Co., 29.V.1959, S.D. & R.H. Burke (1 paratype, TAMUIC); Texas, 0.5 mile west of Minter Springs, Brazos Co., 20.IV.1970, V.V. Board (1, TAMUIC); Texas, Leon Co. 9 km N Flynn, 28.V.1995, *Quercus incanus*, H. & A. Howden (5, CMNC); Texas, Milano, Milam Co., 28.IV.1939, #7315, On tomato plants, very numerous, eating stems particularly, causing plants to fall over (1 paratype, TAMUIC).

**Remarks.** Adults have been collected on *Quercus* sp. (Fagaceae), *Coreopsis* sp. (Asteraceae) and have been reported feeding on tomato plants (Solanaceae) and various herbaceous plants of different families (Fabaceae and Cucurbitaceae). They can be found in pest proportions eating the foliage (Burke, 1959; Howden, 1993a) or the stems.

**Comments.** *Isodacrys burkei* was described based on 91 specimens from five counties in Texas, United States of America: Brazos, Gonzales, Lee, Leon and Milam. The holotype, deposited in TAMUIC, was not examined. The paratypes are distributed in TAMUIC, BMNH, FMNH, CNC, CUIC, Dirección General de Defensa Agrícola (SAGARPA, Mexico), KU, USNM, Howden collection (now housed at the Canadian Museum of Nature) and Kissinger’s collection. Species identity was corroborated by comparison with paratypes examined from TAMUIC and by agreement with the original species description.

The tumescence of elytral intervals two and ten, and intervals five and six at their apical junction varies in degree of development among individuals (Howden, 1961).

**Figure 12:**
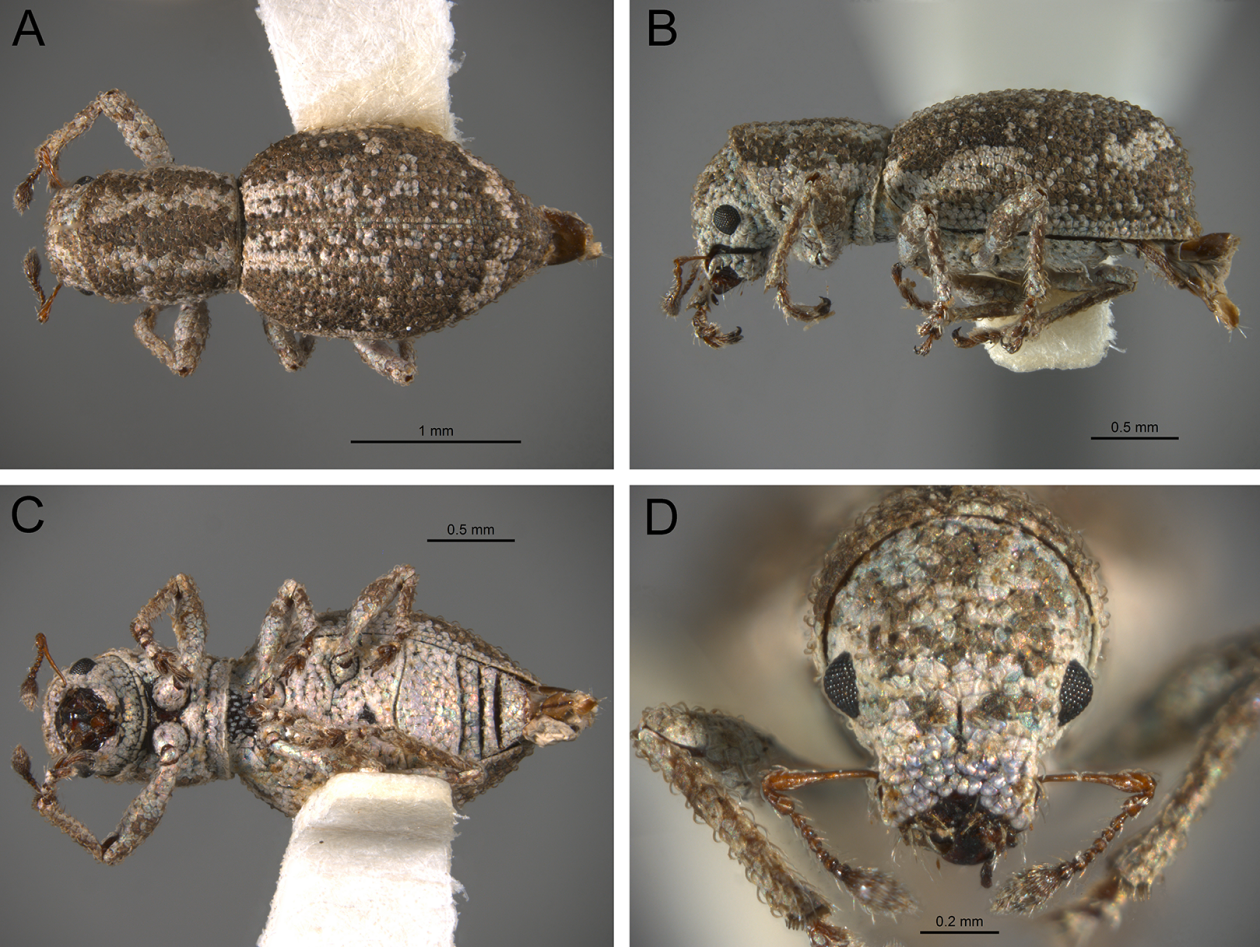
*Isodacrys carlae* new species. (A) Dorsal habitus; (B) lateral habitus; (C) ventral habitus; (D) head in anterior view.

**Figure 13:**
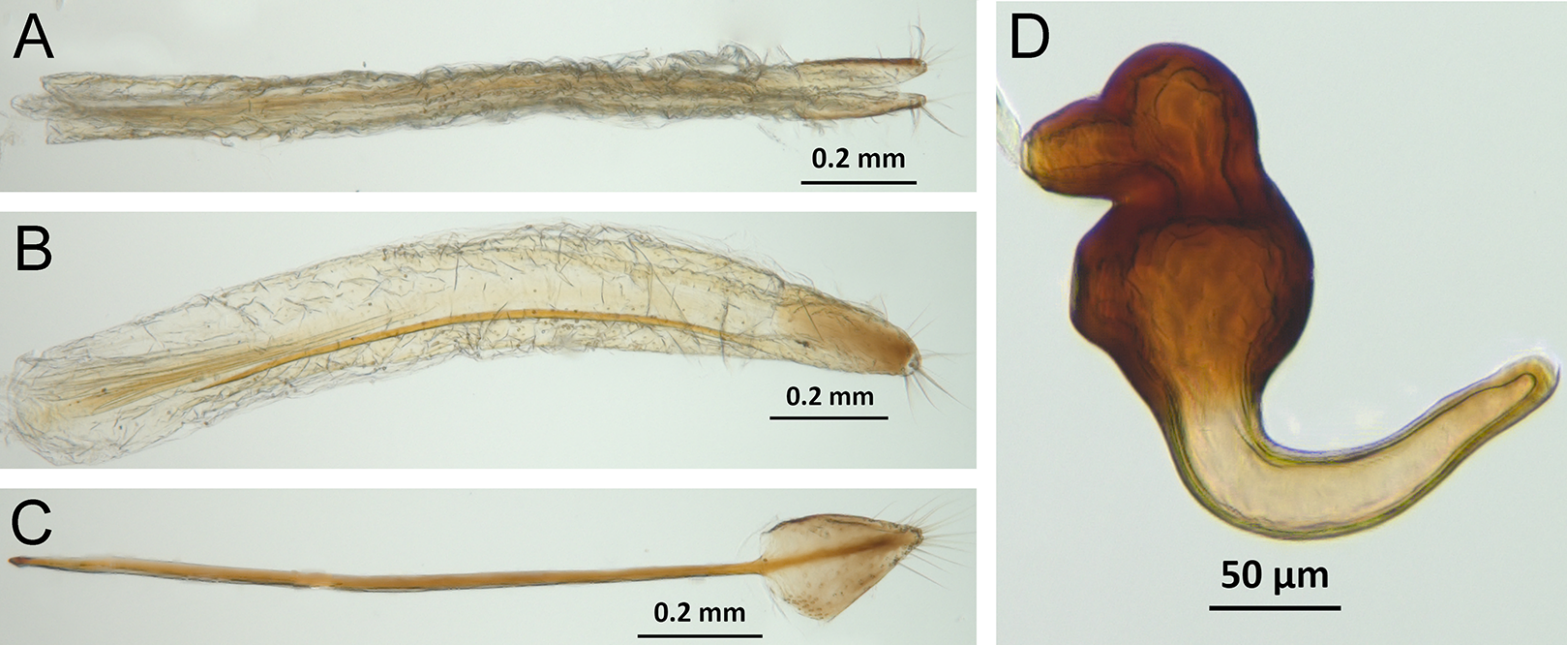
*Isodacrys carlae* new species, female genitalia. (A) Ovipositor in dorsal view; (B) ovipositor in lateral view; (C) sternite VIII in dorsal view; (D) spermatheca.

***Isodacrys carlae* Cortés-Hernández, new species**

(Figs. 4B, 8, 12–13)

urn:lsid:zoobank.org:act:146A1489-52AE-477D-A304-86136B5880BA

**Diagnosis.** Setae lanceolate, completely arched, mostly inconspicuous on temple; rostrum with median sulcus deep, without foveae at its endings (Fig. 12D); scrobe narrow, anterior portion of scrobe about 1/2 length of posterior portion, posterior portion separated from eye by distance 1.5–1.6 times width of scrobe (Fig. 12B); antennal scape without scales (Fig. 12D) or with one or two scales near apex; anterolateral margin of prothorax without postocular vibrissae (Fig. 12B), pronotum with two narrow median longitudinal vittae of white scales (Fig. 12A); profemora 1.2–1.3 times wider than metafemora, inner margin of protibiae without teeth, dorsal surface of first and second or all tarsomeres with appressed scales; and spermatheca peanut-shaped (Fig. 13D).

*Isodacrys carlae* can be readily separated from *I. kuchii* by its narrow scrobe, anterior portion of the scrobe about 1/2 length of posterior portion, and posterior portion separated from eye by distance 1.5–1.6 times width of the scrobe. In *Isodacrys kuchii* the scrobe is broader, anterior portion of the scrobe about 2/3 length of posterior portion and posterior portion of the scrobe separated from eye by distance equal to width of the scrobe. *Isodacrys carlae* can be also confused with *I. confusum* and *I. fasciatum* which differ from *I. carlae* by the scale pattern on the pronotum (white scales medially instead of brown).

**Description - Habitus.** ♀ 2.4–3.0 mm long, 1.0–1.3 mm wide. Scales polygonal, granulate, mainly overlapping irregularly, from brown to white in coloration. Head from vertex to frons mainly covered by brown scales; rostrum and genae covered with white scales. Disc of pronotum covered with brown scales, with two narrow median longitudinal vittae of white scales, continuing along base of elytral intervals two and three (Fig. 12A); sides of prothorax with white scales, with irregular longitudinal brown vittae between anterior and posterior constrictions of prothorax. Basal tenth of elytral intervals two and three, and intervals seven to ten with white scales, epipleura irregularly covered with brown and white scales, interval 10 almost completely covered with white scales, disc of elytra mostly with brown scales, at apical fifth with white fasciae from interval five towards interval one at summit of apical declivity. Setae lanceolate, completely arched, mostly inconspicuous on temple. **Rostrum.** Dorsolateral margins of rostrum subparallel (Fig. 4B); dorsal surface flat, becoming slightly depressed near median sulcus; median sulcus deep, short, extending from posterior margin of epistome to near anterior margin of eyes, without foveae (Fig. 12D). Nasal plate with surface shiny; anterior margin medially indented; posterior margin U/V-shaped, carinate. Epistome in continuous plane with remainder of rostrum, covered by three rows of shiny, smaller scales (Fig. 12D); anterior margin bearing four epistomal setae on each side; posterior margin limiting with median sulcus. Scrobe narrow, deep, bent, reaching ventral surface of rostrum; dorsal margin obtusely angled; anterior portion of scrobe about 1/2 length of posterior portion; posterior portion of scrobe separated from eye by distance 1.5–1.6 times width of scrobe (Fig. 12B). Antenna setose, without scales (Fig. 12D) or with one or two scales near apex of scape; scape capitate; funicular antennomere I clavate, wider and longer than remaining funicular antennomeres; funicular antennomere II clavate; funicular antennomeres III to VII moniliform, distal antennomeres becoming broader. **Head.** Head in lateral view with dorsal outline deflexed at frons towards rostrum; eyes moderate in size, subcircular, prominent, lateral, separated from outline of frons by 4/5 diameter of eye. **Prothorax.** Pronotum in dorsal view subcylindrical, 1.1–1.2 times longer than wide, lateral outlines slightly sinuate (Fig. 12A); in lateral view dorsal outline slightly sinuate (Fig. 12B), anterior and posterior constrictions inconspicuous. Anterolateral margin of prothorax without postocular vibrissae, with row of scales slightly projected anteriorly (Fig. 12B). **Elytra.** 1.8–2.0 times longer than pronotum, at base as wide as base of prothorax. Basal margin of elytra roundly emarginate. Elytra in dorsal view obovate, widest before midlength (Fig. 12A), five intervals visible at base. Elytra in lateral view with dorsal outline evenly curved towards summit of apical declivity; apical declivity with upper two thirds straight, lower third slightly oblique (Fig. 12B). Elytral intervals with no elevations or depressions. **Legs.** Procoxae narrowly separated (Fig. 12C), intercoxal process 1/5–1/4 width of procoxa; profemora fusiform, subequal in length to metafemora, 1.2–1.3 times wider than metafemora; protibiae straight, as long as metatibiae, inner margin without teeth. Dorsal surface of tarsi setose, first and second or all tarsomeres with appressed scales. Tarsal claws free. **Abdomen.** Ventrite III with anterior margin narrowly sulcate, sulcus shallow; sulcus of ventrites IV and V progressively wider and deeper (Fig. 12C); sulci enclosed by lateral margins of ventrites; posterior margin of sulci obliterated, ventrites becoming flat posteriorly. **Genitalia.** ♀: Lamina of sternite VIII triangular, apex acute (Fig. 13C); spiculum ventrale 4.5–4.7 times length of lamina. Ovipositor with distal gonocoxites slightly sclerotized laterally, styli represented by single thickened seta; two ventral baculi close to each other, separated by distance similar to width of one baculus; vagina with two pairs of lightly sclerotized proximal rods (Fig. 13B). Spermatheca peanut-shaped (Fig. 13D); corpus subglobose, narrowed to collum; collum produced, subglobose, funnel-shaped, directed to opposite direction of cornu; ramus broadly tumid, inconspicuous; cornu strongly bent near corpus, then straight, slightly sinuate, apically gradually narrowed.

**Type material.** Holotype ♀: [7000′ nr. Jame,/33 mi. S.E. Saltillo,/Coah. Mex. VII.18′63/A. Howden *Acacia*] [*Isodacrys*/*n.sp*.] (CMNC). Paratypes (45 ♀♀): [7000′ nr. Jame,/33 mi. S.E. Saltillo,/Coah. Mex. VII.18′63/A. Howden *Acacia*] (1, CMNC); [7500′, nr. Jame,/33 mi.S.E.Saltillo,/Coah. Mex. VII.18′63/A. T. Howden] (6, CMNC; 3, MZFC); [7500′ nr. Jame,/33 mi. S.E. Saltillo,/Coah. Mex. VII.25′63/A. T. Howden] (7, CMNC; 1, CNIN; 1 IEXA); [20 mi. S.E. Saltillo,/6000′, Rt. 015, Coah./Mex., VI.20-21, 1971/H. F. Howden]; [MEXICO: Coahuila/12.4 mi S Saltillo/4-VII-1985, J. Woolley/G. Zolnerowich 85/023] [TAMU-ENTO/X0725914] (1, TAMUIC); [MEXICO: Coahuila/12.4 mi S Saltillo/4-VII-1985, J. Woolley/G. Zolnerowich 85/023] [TAMU-ENTO/X0725888] (1, TAMUIC); [MEXICO, Coah., Hwy./57, 15 mi. SE. Saltillo,/7000′ 12Sept.1982 C.&/L.O’Brien & G. Wibmer] (1, ASUCOB; 1, MZFC); [MEX., Coah., 10 mi./E. Saltillo 7000′/VIII-14-1971 C&L/O’Brien & Marshall] (1, ASUCOB]; [MEXICO, Hidalgo,/Municipio Metztitlán/1 km W. San Cristobal,/20.635°N, 98.846°W,/15-VIII-2017, S.M. Clark] (1, ASUCOB); [MEXICO: Nuevo Leon/road to Galeana, 1/km. N jct. rte. 58/17 July 1988/R. Turnbow] (1, CMNC); [MEXICO: Nuevo Leon/9 mi. west Iturbide/July 3, 1974/Clark, Murray,/Ashe, Schaffner] [TAMU-ENTO/X0725413] (1, TAMUIC); [MEXICO: Nuevo Leon/9 mi. west Iturbide/July 3, 1974/Clark, Murray,/Ashe, Schaffner] [TAMU-ENTO/X0724919] (1, TAMUIC); [MEXICO: Nuevo Leon/9 mi. west Iturbide/July 3, 1974/Clark, Murray,/Ashe, Schaffner] [TAMU-ENTO/X0725718] (1, IEXA); [MEXICO: Nuevo Leon/9 mi. west Iturbide/July 3, 1974/Clark, Murray,/Ashe, Schaffner] [TAMU-ENTO/X0726871] (1, TAMUIC); [MEXICO: Nuevo Leon/9 mi. west Iturbide/July 3, 1974/Clark, Murray,/Ashe, Schaffner] [TAMU-ENTO/X0730294] (1, TAMUIC); [MEXICO: Nuevo Leon/9 mi. west Iturbide/July 3, 1974/Clark, Murray,/Ashe, Schaffner] [TAMU-ENTO/X0726000] (1, CNIN); [MEX. N.L. 40 mi./NW Jcn. Hwy 57&60/VI-22-1971 C.W./O’Brien & Marshall] (1, ASUCOB; 1, MZFC); [MEXICO, N.L., Hwy./58, 32 mi. W. Linares,/6000′ 11Sept.1982 C.&/L. O’Brien & G. Wibmer] (3, ASUCOB); [MEXICO, N.L., 11 km./NE. S. Antonio Peña/Nevada, 2,000 m. Aug16/1977A.N.Garcia A.] [beating pine and/miscellaneous] (1, ASUCOB); [MEX., N.L., 29 mi./SW. Linares 5700′/VIII-15-1971 C&L/O’Brien & Marshall] (1, ASUCOB); [MEXICO: San Luis Potosi/4 km. W Guadalcazar/18 July 1988/R. Turnbow] [*Isodacrys*/*geminatum* Howd./det. R.S. Anderson 90] (1, CMNC); [MEXICO: Tamaulipas/19.6 mi. sw. Jaumave/18-VII-1973/Gaumer and Clark] [TAMU-ENTO/X0725801] [*Isodacrys*/*sp*./DET./A. Howden] (1, TAMUIC); [MEXICO: Tamaulipas/19.6 mi. sw. Jaumave/18-VII-1973/Gaumer and Clark] [TAMU-ENTO/X0727602] (1, TAMUIC); [MEXICO: Tamaulipas/19.6 mi. sw. Jaumave/18-VII-1973/Gaumer and Clark] [TAMU-ENTO/X0725553] (1, TAMUIC); [MEXICO, Tam., Hwy./101, 28 mi. NE. Tula/4500′ 22July1982 CW&/L. O’Brien & G. Wibmer] (1, ASUCOB).

**Etymology.** This species is named after the first author’s beloved mother, Carla Angélica Hernández-Olvera, for all her support and endless kindness.

**Remarks.** Adults have been collected on *Acacia* (Fabaceae) and beating miscellaneous vegetation between ~1,370 and 2,130 m above sea level. Males remain unknown.

**Comments.** This species had been previously recognized as an undescribed *Isodacrys* species by Anne Howden (as labels on the herein designated holotype indicate) and subsequently (perhaps following Howden) by Charles W. O’Brien in 1999 according to revised material at ASUCOB.

**Figure 14:**
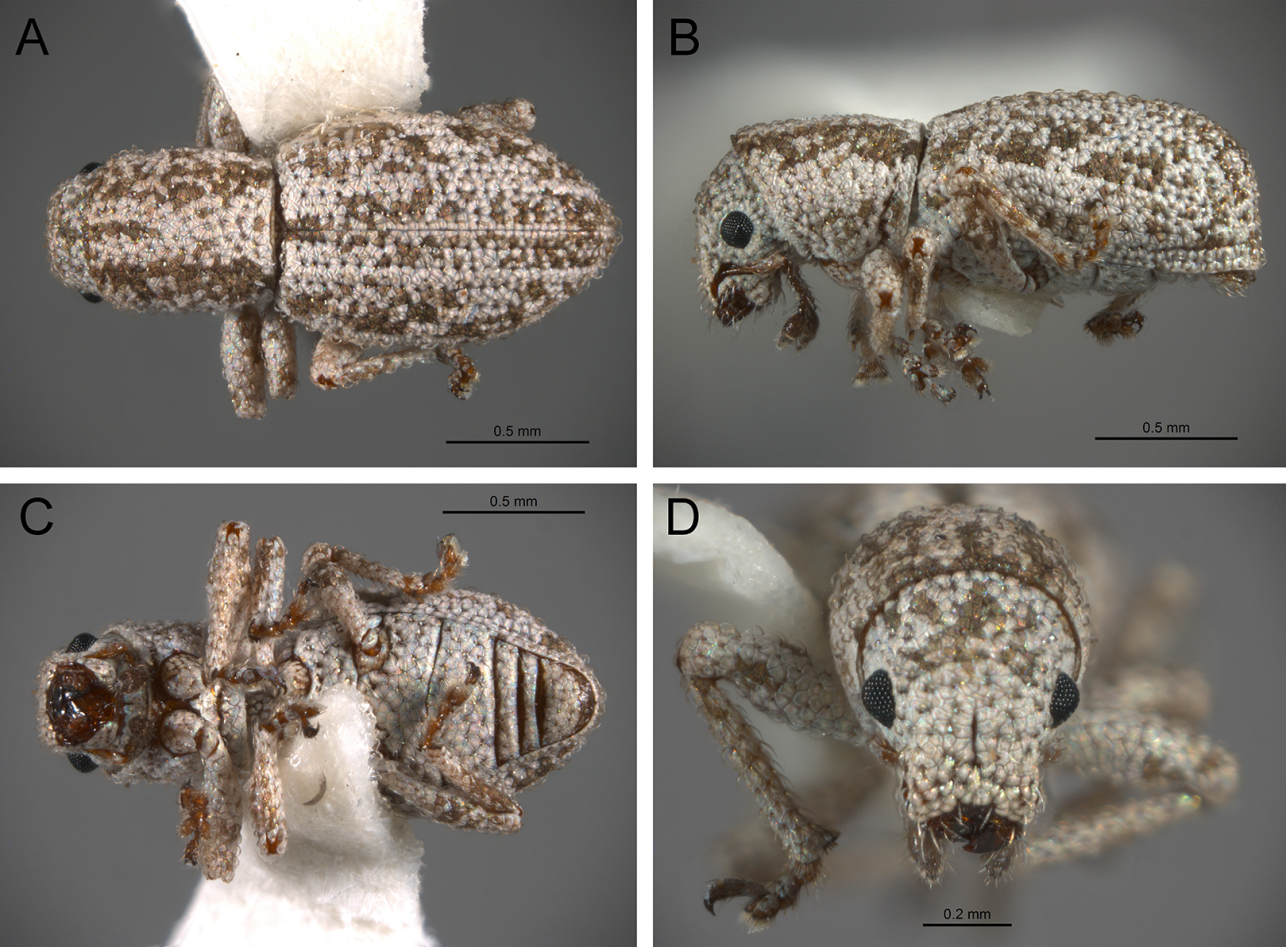
*Isodacrys confusum* new species. (A) Dorsal habitus; (B) lateral habitus; (C) ventral habitus; (D) head in anterior view.

**Figure 15:**
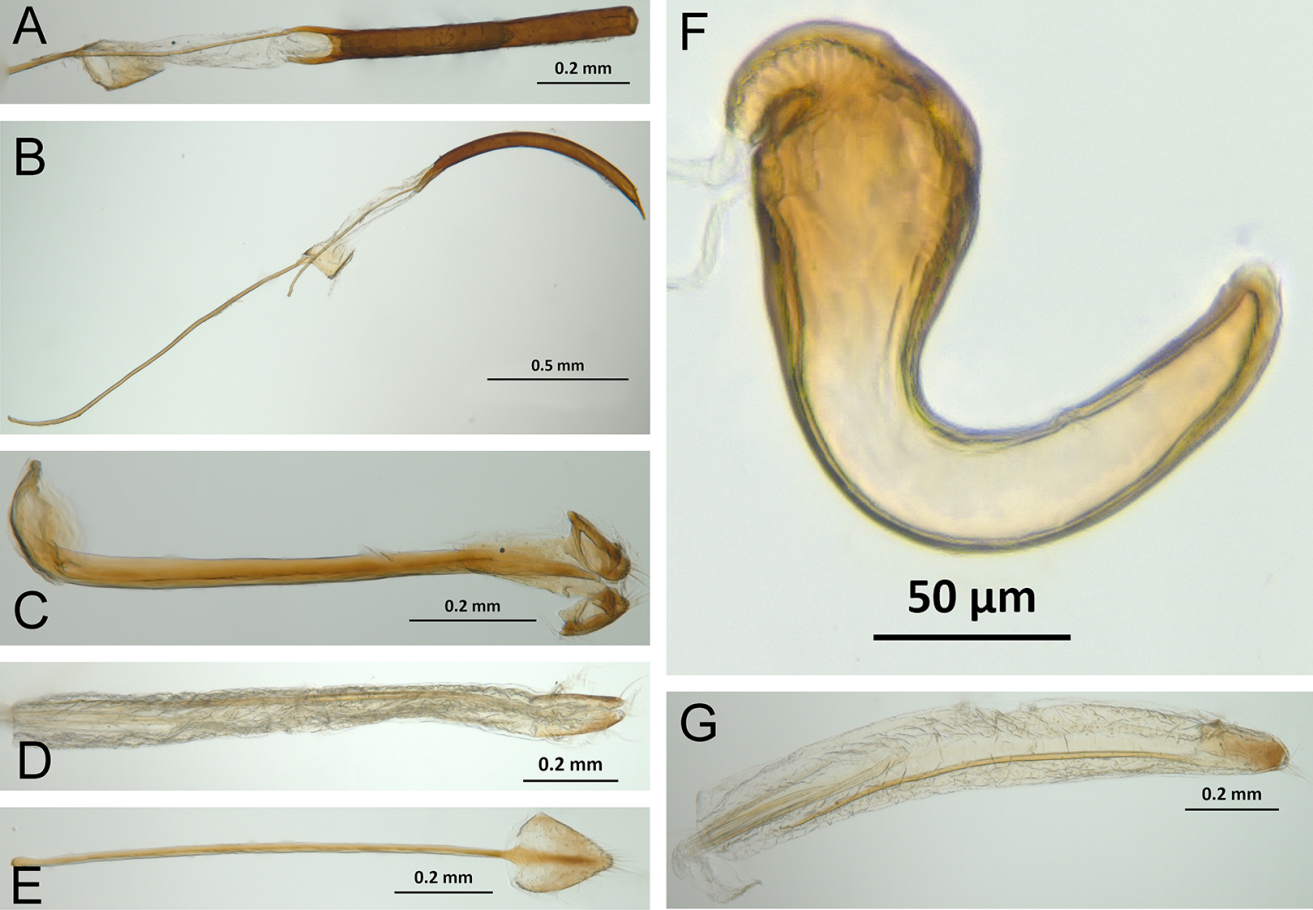
*Isodacrys confusum* new species, genitalia. (A) Median lobe in dorsal view; (B) median lobe and tegmen in lateral view; (C) male hemisternites VIII and sternite IX in dorsal view; (D) ovipositor in dorsal view; (E) female sternite VIII in dorsal view; (F) spermatheca; (G) ovipositor in lateral view.

***Isodacrys confusum* Cortés-Hernández, new species**

(Figs. 7A, 8, 14–15)

urn:lsid:zoobank.org:act:3EDF5293-38FD-4217-8730-164074806BAA

**Diagnosis.** Setae lanceolate, completely arched; rostrum with median sulcus short, without foveae at its endings; scrobe deep, anterior portion of scrobe about 2/3 length of posterior portion, posterior portion of scrobe separated from eye by distance 1.4–1.6 times width of scrobe; antennal scape covered with scales at apex dorsally (Fig. 14B); anterolateral margin of prothorax without postocular vibrissae (Fig. 14B), pronotum covered with white scales medially, with two broad, lateral longitudinal vittae of brown scales (Fig. 14A); profemora 1.2–1.3 times wider than metafemora, inner margin of protibiae without teeth, all tarsomeres with appressed scales (Fig. 14D); male terminalia with manubrium longer than median lobe (Fig. 15B); spermatheca comma-shaped, collum produced, bent basally to ramus, contiguous with corpus (Fig. 15F).

*Isodacrys confusum* can be easily confused with *I. carlae*, *I. kuchii* or *I. fasciatum*, but it can be externally separated from *I. carlae* and *I. kuchii* by its scale pattern on the prothorax, which is covered with white scales medially (covered with brown scales medially instead of white in *I. carlae* and *I. kuchii*) and by the scape covered with scales dorsally (always lacking in *I. kuchii* and generally lacking in *I. carlae*). Finally, *I. confusum* can be separated from *Isodacrys fasciatum* by the scape covered with scales and by the spermatheca with collum directed towards ramus, contiguous to corpus. The scape is not covered with scales and the ramus is not contiguous to the corpus in *I. fasciatum*.

**Description - Habitus.** ♂ 1.7–2.5 mm long, 0.7–1.1 mm wide; ♀ 2.4–2.6 mm long, 1.1–1.2 mm wide. Scales polygonal, granulate, mainly overlapping irregularly, from brown to white in coloration. Head and rostrum mainly covered by white scales, with irregular patches of brown scales on vertex and frons. Disc of pronotum covered with white scales medially, with two broad, lateral longitudinal vittae of brown scales (Fig. 14A); sides of prothorax with white scales, with irregular longitudinal brown vittae between anterior and posterior constrictions of prothorax. Disc of elytra irregularly with white and brown scales, intervals six to four at second fourth with brown fasciae, apical third with brown fascia from interval six towards interval one at summit of apical declivity; epipleura mostly with white scales. Setae lanceolate, completely arched. **Rostrum.** Dorsolateral margins of rostrum subparallel; dorsal surface flat, becoming slightly depressed near median sulcus; median sulcus short, extending from posterior margin of epistome to near anterior margin of eyes, without foveae (Fig. 14D). Nasal plate with surface shiny; anterior margin medially indented; posterior margin V-shaped, carinate. Epistome in continuous plane with remainder of rostrum, covered by three rows of shiny, smaller scales (Fig. 14D); anterior margin bearing four or five epistomal setae on each side; posterior margin limiting with median sulcus. Scrobe deep, bent, reaching ventral surface of rostrum; dorsal margin obtusely angled; anterior portion of scrobe about 2/3 length of posterior portion; posterior portion of scrobe separated from eye by distance 1.4–1.6 times width of scrobe. Antenna setose, apex of scape covered with scales (Fig. 14B); scape capitate; funicular antennomere I clavate, wider and longer than remaining funicular antennomeres; funicular antennomere II clavate, longer than remaining funicular antennomeres; funicular antennomeres III–VII moniliform, distal antennomeres becoming broader. **Head.** Head in lateral view with dorsal outline deflexed at frons towards rostrum; eyes moderate in size, subcircular, prominent, lateral, separated from dorsal outline of frons by distance similar to diameter of eye. **Prothorax.** Pronotum in dorsal view subcylindrical, nearly as wide as long, lateral outlines slightly sinuate; in lateral view dorsal outline slightly sinuate, anterior and posterior constrictions inconspicuous. Anterolateral margin of prothorax without postocular vibrissae (Fig. 14B), with row of scales slightly projected anteriorly. **Elytra.** 1.8–2.0 times longer than pronotum, at base as wide as base of prothorax. Basal margin of elytra roundly emarginate. Elytra in dorsal view obovate, widest before midlength (Fig. 14A), five intervals visible at base. Elytra in lateral view with dorsal outline gently evenly curved towards summit of apical declivity; apical declivity with upper two thirds straight, lower third slightly oblique. Elytral intervals with no elevations or depressions. **Legs.** Procoxae narrowly separated (Fig. 14C), intercoxal process 1/6–1/4 width of procoxa; profemora fusiform, subequal in length to metafemora, 1.2–1.3 times wider than metafemora; protibiae straight, as long as metatibiae, inner margin without teeth. Dorsal surface of tarsi setose, all tarsomeres with appressed scales (Fig. 14D). Tarsal claws free. **Abdomen.** Ventrite III with anterior margin narrowly sulcate, sulcus shallow; sulcus of ventrites IV and V progressively wider and deeper (Fig. 14C); sulci enclosed by lateral margins of ventrites; posterior margin of sulci obliterated, ventrites becoming flat posteriorly. **Genitalia.** ♂: hemisternites VIII teardrop-shaped, outer corner not truncate (Fig. 15C); basal plate of sternite IX lightly sclerotized, distally bifurcate, tips of bifurcations sclerotized; spiculum gastrale as long as median lobe, distally continuing through basal plate; ring of tegmen without parameres; manubrium 1.5 times longer than median lobe (Fig. 15B); temones 4/5 length of median lobe; median lobe 12 times longer than wide in dorsal view, evenly curved in lateral view (Fig. 15B), apical opening elliptical, apex sharply acute. ♀: Lamina of sternite VIII triangular, apex acute (Fig. 15E); spiculum ventrale 4.3–4.6 times length of lamina. Ovipositor with distal gonocoxites slightly sclerotized laterally, styli represented by single thickened seta; two ventral baculi close to each other, separated by distance similar to width of one baculus; vagina with two pairs of lightly sclerotized proximal rods (Fig. 15G). Spermatheca comma-shaped; corpus slightly subglobose, narrowing towards cornu; collum produced, subcylindrical, bent basally to ramus, contiguous with corpus; ramus from indistinct to slightly tumid; cornu strongly curved near corpus, then evenly curved, elongate, reaching apex of ramus, apically gradually narrowed (Fig. 15F). **Sexual dimorphism.** Ventrites I and II slightly concave mesally in males, convex in females; apex of ventrite V slightly truncate in males, rounded in females. Males are usually distinctively smaller and thinner than females.

**Type material.** Holotype ♂: [MEXICO: Tamaulipas/16.3 mi. ne. Jaumave/18-VII.1973/Gaumer and Clark] [taken on/Mimosa/monancistra] [TAMU-ENTO/X0729035] (TAMUIC); paratypes (8 ♂♂, 28 ♀♀): [MEXICO, Tam., Hwy 101/18 mi. SW. Cd. Victoria,/4300′ 22July1982 C.W.&/L.O’Brien & G. Wibmer] (1 ♂, 1 ♀, ASUCOB; 1 ♂, MZFC); [MEXICO, Tam., Hwy 101/24 mi. SW. Cd. Victoria,/2150′ 23July1982 C.W.&/L. O’Brien & G. Wibmer] (1 ♂, 2 ♀, ASUCOB); [MEXICO, Tam., Hwy 101/22 mi. SW. Cd. Victoria,/3000′ 22July1982 C.W.&/L. O’Brien & G. Wibmer] (1 ♂, 1 ♀, ASUCOB); [MEXICO: Tamaulipas/same data as holotype] [taken on/same data as holotype] [TAMU-ENTO/X0726618] (1 ♀, TAMUIC); [MEXICO: Tamaulipas/same data as holotype] [taken on/same data as holotype] [TAMU-ENTO/X0727115] (1 ♀, TAMUIC); [MEXICO: Tamaulipas/same data as holotype] [taken on/same data as holotype] [TAMU-ENTO/X0725169] (1 ♀, TAMUIC); [MEXICO: Tamaulipas/same data as holotype] [taken on/same data as holotype] [TAMU-ENTO/X0724605] (1 ♀, TAMUIC); [MEXICO: Tamaulipas/same data as holotype] [taken on/same data as holotype] [TAMU-ENTO/X0725410] (1 ♀, TAMUIC); [MEXICO: Tamaulipas/same data as holotype] [taken on/same data as holotype] [TAMU-ENTO/X0726220] (1 ♀, TAMUIC); [MEXICO: Tamaulipas/same data as holotype] [taken on/same data as holotype] [TAMU-ENTO/X0726502] (1 ♀, TAMUIC); [MEXICO: Tamaulipas/same data as holotype] [taken on/same data as holotype] [TAMU-ENTO/X0727010] (1 ♂, TAMUIC); [MEXICO: Tamaulipas/same data as holotype] [taken on/same data as holotype] [TAMU-ENTO/X0726280] (1 ♀, TAMUIC); [MEXICO: Tamaulipas/same data as holotype] [taken on/same data as holotype] [TAMU-ENTO/X0725437] (1 ♀, TAMUIC); [MEXICO: Tamaulipas/same data as holotype] [taken on/same data as holotype] [TAMU-ENTO/X0725446] (1 ♀, TAMUIC); [MEXICO: Tamaulipas/same data as holotype] [taken on/same data as holotype] [TAMU-ENTO/X0725407] (1 ♀, TAMUIC); [MEXICO: Tamaulipas/same data as holotype] [taken on/same data as holotype] [TAMU-ENTO/X0725141] (1 ♀, TAMUIC); [MEXICO: Tamaulipas/same data as holotype] [taken on/same data as holotype] [TAMU-ENTO/X0725168] (1 ♀, TAMUIC); [MEXICO: Tamaulipas/same data as holotype] [taken on/same data as holotype] [TAMU-ENTO/X0725412] (1 ♀, TAMUIC); [MEXICO: Tamaulipas/same data as holotype] [taken on/same data as holotype] [TAMU-ENTO/X0726973] (1 ♀, IEXA); [MEXICO: Tamaulipas/same data as holotype] [taken on/same data as holotype] [TAMU-ENTO/X0725112] (1 ♀, IEXA); [MEXICO: Tamaulipas/same data as holotype] [taken on/same data as holotype] [TAMU-ENTO/X0725393] (1 ♀, CNIN); [MEXICO: Tamaulipas/same data as holotype] [taken on/same data as holotype] [TAMU-ENTO/X0729284] (1 ♀, CNIN); [MEXICO: Tamaulipas/same data as holotype] [taken on/same data as holotype] [TAMU-ENTO/X0728579] (1 ♂, TAMUIC); [MEXICO: Tamaulipas/same data as holotype] [taken on/same data as holotype] [*Isodacrys*/*sp*./DET./A. Howden] [TAMU-ENTO/X0728911] (1 ♀, MZFC); [MEXICO: Tamaulipas/same data as holotype] [taken on/same data as holotype] [TAMU-ENTO/X0728902] [♂] (1 ♂, MZFC); [MEXICO: Tamaulipas/same data as holotype] [TAMU-ENTO/X0726515]; [MEXICO: Tamaulipas/same data as holotype] [taken on/same data as holotype] [*Isodacrys*/*geminatum*/Howden/det. R.S. Anderson 1981] (1 ♀, CMNC); [MEXICO: Tamaulipas/same data as holotype] [taken on/same data as holotype] (1 ♂, 3 ♀♀, CMNC).

**Etymology.** This species is named with the Latin word *confusum* meaning confused, because of its external resemblance with several species of *Isodacrys*.

**Remarks.** Most of the type series was collected on *Mimosa monancistra* Benth. (Fabaceae). Adults have been collected between ~650 and 1,220 m above sea level.

***Isodacrys crispum* Howden, 1961**

(Figs. 1 and 8)

*Isodacrys crispum* Howden, 1961: 84–85. Holotype: ♀, not examined. O’Brien & Wibmer, 1982: 46 (checklist); Morrone, 1999: 145 (checklist); Cortes-Hernández & Morrone, 2019: 50.

**Diagnosis.** Scales with carinate margins; setae lanceolate, completely arched; rostrum short; scrobe right-angled, anterior portion shorter than posterior portion, posterior portion separated from eye by distance equal to width of scrobe; anterolateral margin of prothorax with one to three vestigial postocular vibrissae; sulci of ventrites III, IV and V with posterior margin sharp, conspicuously delimiting sulci; prolegs not larger than metalegs, inner margin of protibiae with teeth; spermatheca comma-shaped, corpus subglobose, cornu inconspicuous, ramus slightly tumid.

*Isodacrys crispum* can be readily separated from similar *Isodacrys* species by its scales with carinate margins, short rostrum, the prothorax with only one to three vestigial postocular vibrissae, sulci of ventrites III, IV and V with posterior margin sharp, conspicuously delimiting sulci and the protibiae with teeth along inner margin. Although *Isodacrys crispum* can be superficially confused with *Isodrusus debilis*, it is easily separated by the absence of humeri and the tarsal claws free. *Isodrusus debilis* has well-developed humeri and tarsal claws connate.

**Material examined.** MEXICO: Durango, 25 mi. W Durango, 10.VI.1964, Oak, H. Howden (5, CMNC); Durango, 25 mi. W Durango, 29.VI.1964, H. Howden (30, CMNC; 2 ASUCOB); Durango, 25 mi. W Durango, 3.VII.1964, H. & A. Howden (3, CNC); Durango, 28 mi. W Durango, 9.VI.1967, *Quercus clivicola F. consanguinea*, C.H. Maen (1, CMNC); Durango, 25 mi. W Durango, 18.VIII.1974, on *Quercus*, 7800′, C.W. & L. O’Brien, Marshall (2, UAQE); Durango, 25 mi. W Durango, 18.VIII.1974, on pine/on *Quercus*, 7800′, C.W. & L. O’Brien, Marshall (34, ASUCOB); Durango, 26.5 mi. SW Durango, 21.VII.1982, General sweeping, Fred. G. Andrews (9, CMNC); Zacatecas, 27 mi NW Fresnillo, Hwy 45, 17.VIII.1974, on *Quercus*, 7800′, C.W. & L. O’Brien, Marshall (32, ASUCOB).

**Remarks.** Some adults have been collected on *Quercus* sp. (Fagaceae) and *Pinus* sp. (Pinaceae), at ~2,370 m above sea level. *Isodacrys crispum* has a strong superficial resemblance to *Isodrusus debilis* (Howden, 1961). Males remain unknown.

**Comments.** *Isodacrys crispum* was described from only one specimen from Palos Colorados, Durango, Mexico. The holotype, deposited in AMNH, was not examined. Identity of material examined was corroborated from specimens identified by Anne Howden and by agreement with the original species description.

***Isodacrys ellipticum* Howden, 1961**

(Fig. 8)

*Isodacrys ellipticum* Howden, 1961: 86–87. Holotype: ♀, not examined. O’Brien & Wibmer, 1982: 46 (checklist); Morrone, 1999: 145 (checklist).

**Diagnosis.** Setae of elytra spatulate, long, from semierect to erect; epistome with longitudinal carina joined with posterior margin of nasal plate (e.g., Fig. 4C); scrobe with anterior portion as long as posterior portion; anterolateral margin of prothorax without postocular vibrissae; elytra in dorsal view subelliptical, widest at midlength, with seven intervals visible at base; profemora wider than metafemora, protibiae longer than metatibiae, inner margin of protibiae with teeth; and spermatheca u-shaped, corpus subcylindrical, slightly swollen, collum inconspicuous, ramus very short, broadly cylindrical.

*Isodacrys ellipticum* can be separated from *I. guatemalenum* by the spatulate shape of its elytral setae, from semierect to erect and spermatheca with collum inconspicuous, ramus very short. In *I. guatemalenum* the elytral setae are parallel-sided, usually longer and erect, and in the spermatheca both the collum and the ramus are inconspicuous.

**Material examined.** GUATEMALA: Zacapa, San Lorenzo Quarry Road, 3–7 km N Sta. Cruz, CA9, 17.VII.2008, Dry forest, C. & L. O’Brien, F. Skillman (1, ASUCOB). HONDURAS: Comayagua, Comayagua, 2.VI.1978, Gary V. Manley (1, CMNC); Cortés, 5 km N Cofradía, 5.VIII.1977, C. & L. O’Brien, G. Marshall (41, ASUCOB); Francisco Morazán, Distrito Central, 5 mi. S Tegucigalpa, 9.VI.1974, night, 4000′, C. & L. O’Brien, G. Marshall (3, CASENT); Francisco Morazán, 17 mi. NE Talanga, 16.VI.1974, 2900′, C. & L. O’Brien, G. Marshall (129, ASUCOB; 3, CASENT); Francisco Morazán, 23.8 km SW Talanga, 3.VI.1993, R. Turnbow (1, CMNC); Francisco Morazán, Tamara valley, 5.X.1993, R. Turnbow (1, CMNC).

**Remarks.** The holotype was collected in flowers of *Lantana* sp. (Verbenaceae; see Howden, 1961). Adults have been collected between ~880 and 1,200 m above sea level. Males remain unknown.

**Comments.** *Isodacrys ellipticum* was described from one specimen from Honduras. The holotype, deposited in USNM, was not examined. Identity of material examined was corroborated from specimens identified by Anne Howden and by agreement with the original species description.

**Figure 16:**
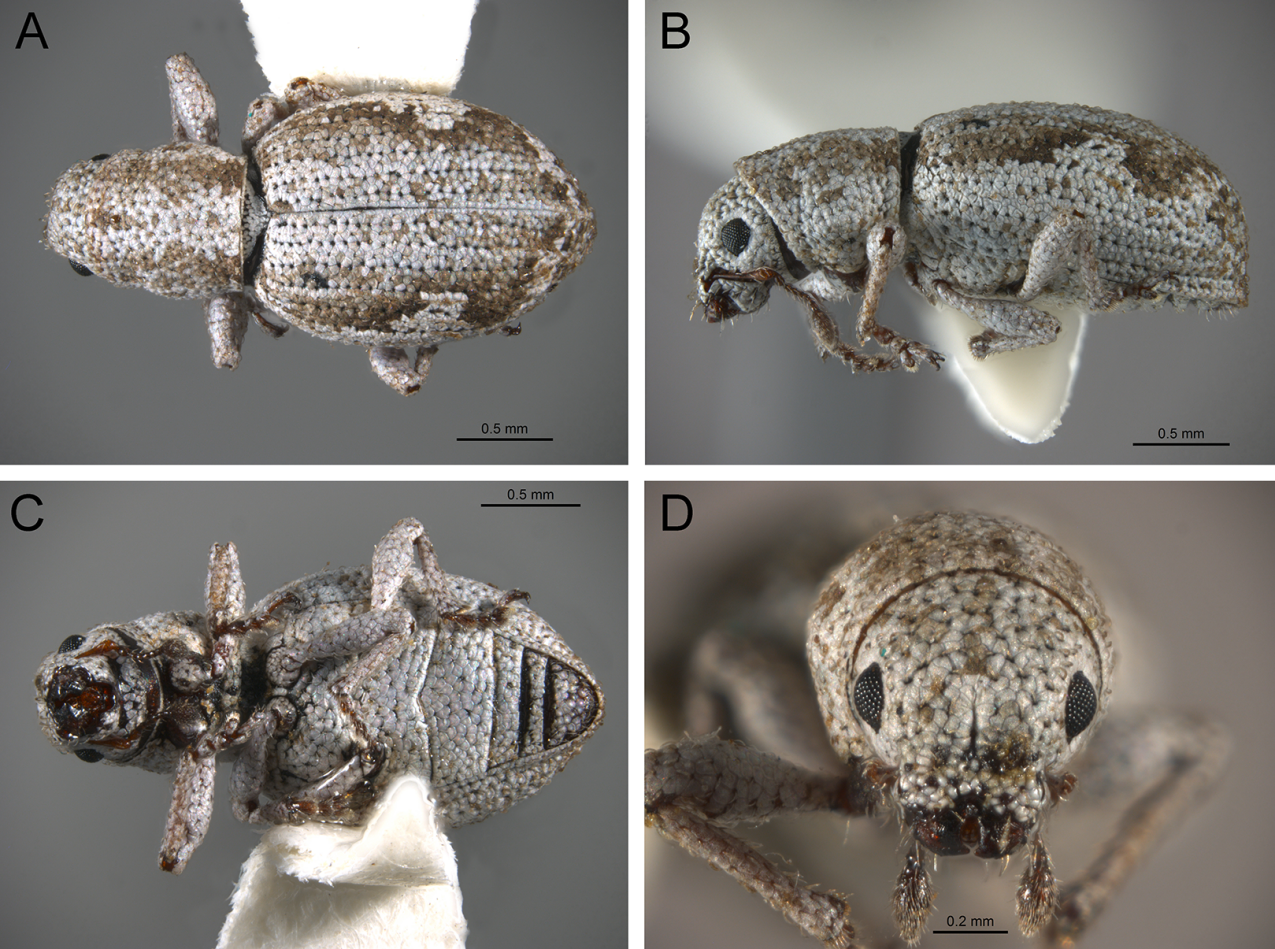
*Isodacrys fasciatum* new species. (A) Dorsal habitus; (B) lateral habitus; (C) ventral habitus; (D) head in anterior view.

**Figure 17:**
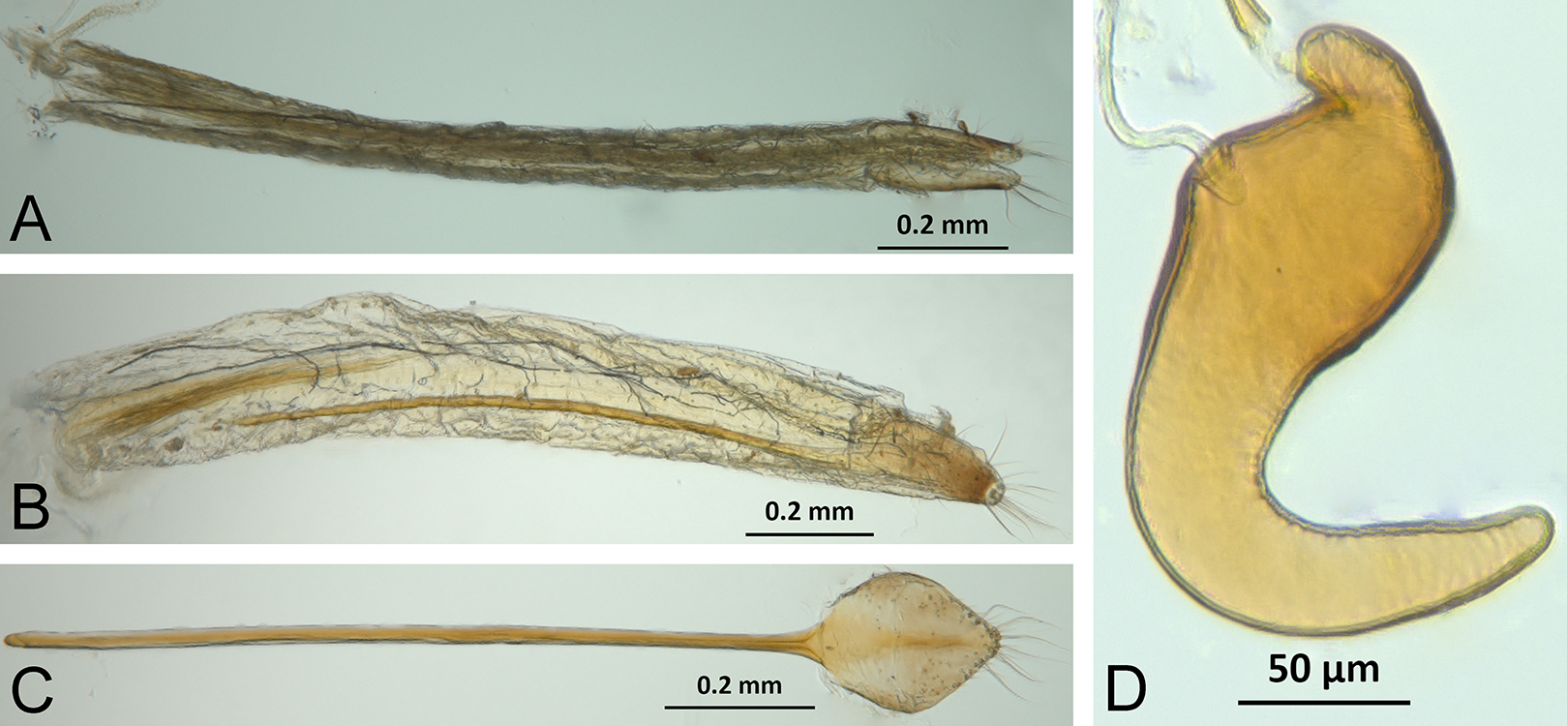
*Isodacrys fasciatum* new species, female genitalia. (A) Ovipositor in dorsal view; (B) ovipositor in lateral view; (C) sternite VIII in dorsal view; (D) spermatheca.

***Isodacrys fasciatum* Cortés-Hernández, new species**

(Figs. 2, 3C, 5E, 9, 16–17)

urn:lsid:zoobank.org:act:881D4538-9CB8-49E5-9641-CCF3973623E1

**Diagnosis.** Setae lanceolate, completely arched; rostrum with median sulcus short, without foveae at its endings (Fig. 16D); posterior portion of scrobe separated from eye by distance 1.5–2.1 times width of scrobe; antennal scape without scales (Fig. 16B); anterolateral margin of prothorax without postocular vibrissae (Fig. 16B), pronotum covered with white scales medially, with two broad, lateral longitudinal vittae of brown scales (Fig. 16A); profemora 1.2 times wider than metafemora, inner margin of protibiae without teeth, all tarsomeres with appressed scales; and spermatheca comma-shaped with collum not contiguous with corpus (Fig. 17D).

This species is very similar to *Isodacrys confusum*, nonetheless, in *I. fasciatum* the banded scale pattern along the elytra is more conspicuous. Furthermore, *I. fasciatum* does not present scales at the apex of the scape, and the collum of the spermatheca is not contiguous with the corpus like in *I. confusum*.

**Description - Habitus.** ♀ 2.1–2.9 mm long, 1.0–1.3 mm wide. Scales polygonal, granulate, mainly overlapping irregularly, from brown to white in coloration. Head and rostrum mainly covered by white scales, with irregular patches of brown scales from vertex to frons. Disc of pronotum covered with white scales medially, with two broad, lateral longitudinal vittae of brown scales (Fig. 16A); sides of prothorax with white scales, often with small, irregular patches of light brown scales. Disc of elytra mainly covered with white scales, interval six at basal third with brown scales, then forming a brown fascia with intervals five and four at second third, apical third with brown fascia from interval six to interval one at summit of apical declivity; epipleura mostly with white scales. Setae lanceolate, completely arched. **Rostrum.** Dorsolateral margins of rostrum subparallel; dorsal surface flat, becoming slightly depressed near median sulcus; median sulcus deep, short, from posterior margin of epistome to near anterior margin of eyes, without foveae (Fig. 16D). Nasal plate with surface shiny; anterior margin medially indented; posterior margin V-shaped, carinate. Epistome in continuous plane with remainder of rostrum, covered by three rows of shiny, smaller scales (Fig. 16D); anterior margin bearing four epistomal setae on each side; posterior margin limiting with median sulcus. Scrobe deep, bent, reaching ventral surface of rostrum; dorsal margin obtusely angled; anterior portion of scrobe about 1/2 length of posterior portion; posterior portion of scrobe separated from eye by distance 1.5–2.1 times width of scrobe. Antenna setose, without scales (Fig. 16B); scape capitate; funicular antennomere I clavate, wider and longer than remaining antennomeres; funicular antennomere II clavate, longer than remaining funicular antennomeres; funicular antennomeres III–VII moniliform, distal antennomeres becoming broader. **Head.** Head in lateral view with dorsal outline deflexed at frons towards rostrum; eyes moderate in size, subcircular, prominent, lateral, separated from outline of frons by about 4/5 diameter of eye. **Prothorax.** Pronotum in dorsal view subcylindrical, as wide as long, lateral outlines sinuate (Fig. 16A); in lateral view dorsal outline slightly sinuate, anterior and posterior constrictions inconspicuous. Anterolateral margin of prothorax without postocular vibrissae (Fig. 16B), with row of scales slightly projected anteriorly. **Elytra.** 2.0–2.2 times longer than pronotum, at base as wide as base of prothorax. Basal margin of elytra roundly emarginate. Elytra in dorsal view obovate, widest before midlength, five intervals visible at base (Fig. 5E). Elytra in lateral view with dorsal outline gently evenly curved towards summit of apical declivity; apical declivity with upper two thirds straight, lower third slightly oblique. Elytral intervals with no elevations or depressions. **Legs.** Procoxae narrowly separated, intercoxal process 1/6–1/4 width of procoxa; profemora fusiform, subequal in length to metafemora, 1.2 times wider than metafemora; protibiae straight, as long as metatibiae, inner margin without teeth. Dorsal surface of tarsi setose, all tarsomeres with appressed scales. Tarsal claws free. **Abdomen.** Ventrite III with anterior margin narrowly sulcate, sulcus shallow; sulcus of ventrites IV and V progressively wider and deeper (Fig. 16C); sulci enclosed by lateral margins of ventrites; posterior margin of sulci obliterated, ventrites becoming flat posteriorly. **Genitalia.** ♀: Lamina of sternite VIII triangular, apex acute (Fig. 17C); spiculum ventrale 4.0–4.2 times length of lamina. Ovipositor with distal gonocoxites slightly sclerotized laterally, styli represented by single thickened seta; two ventral baculi close to each other, separated by distance similar to width of one baculus; vagina with two pairs of lightly sclerotized proximal rods (Fig. 17B). Spermatheca comma-shaped; corpus slightly subglobose, narrowing towards cornu; collum produced, subcylindrical, bent basally to ramus, not contiguous with corpus; ramus slightly tumid; cornu strongly curved near corpus, then evenly curved, elongate, reaching apex of ramus, apically gradually narrowed (Fig. 17D).

**Type material.** Holotype ♀: [MEX., Coah., 10 mi./E. Saltillo 7000′/VIII-14-1971 C&L/O’Brien & Marshall] (ASUCOB). Paratypes (11 ♀♀): [MEX., Coah.,10 mi./same data as holotype] (2, ASUCOB); [MEXICO: Coahuila/12.4 mi S Saltillo/4-VII-1985, J. Woolley/G. Zolnerowich 83/023] [TAMU-ENTO/X0727876] (1, TAMUIC); [MEXICO, Dgo., 22 mi./NE. Durango, 6200′/Aug. 20, 1974 C.W. & L./O’Brien & Marshall] (4, ASUCOB; 2, MZFC; 1, CMNC); [MEX. N.L. 10 mi./S. Jcn. 57 & 60/VI-23-1971 L&CW/O’Brien & Marshall] (1, ASUCOB).

**Etymology.** Named with the Latin word *fascia*, referring to the white/brown banded scale pattern visible along the elytra.

**Remarks.** Adults have been collected between ~1,890 and 2,130 m above sea level. Males remain unknown.

**Figure 18:**
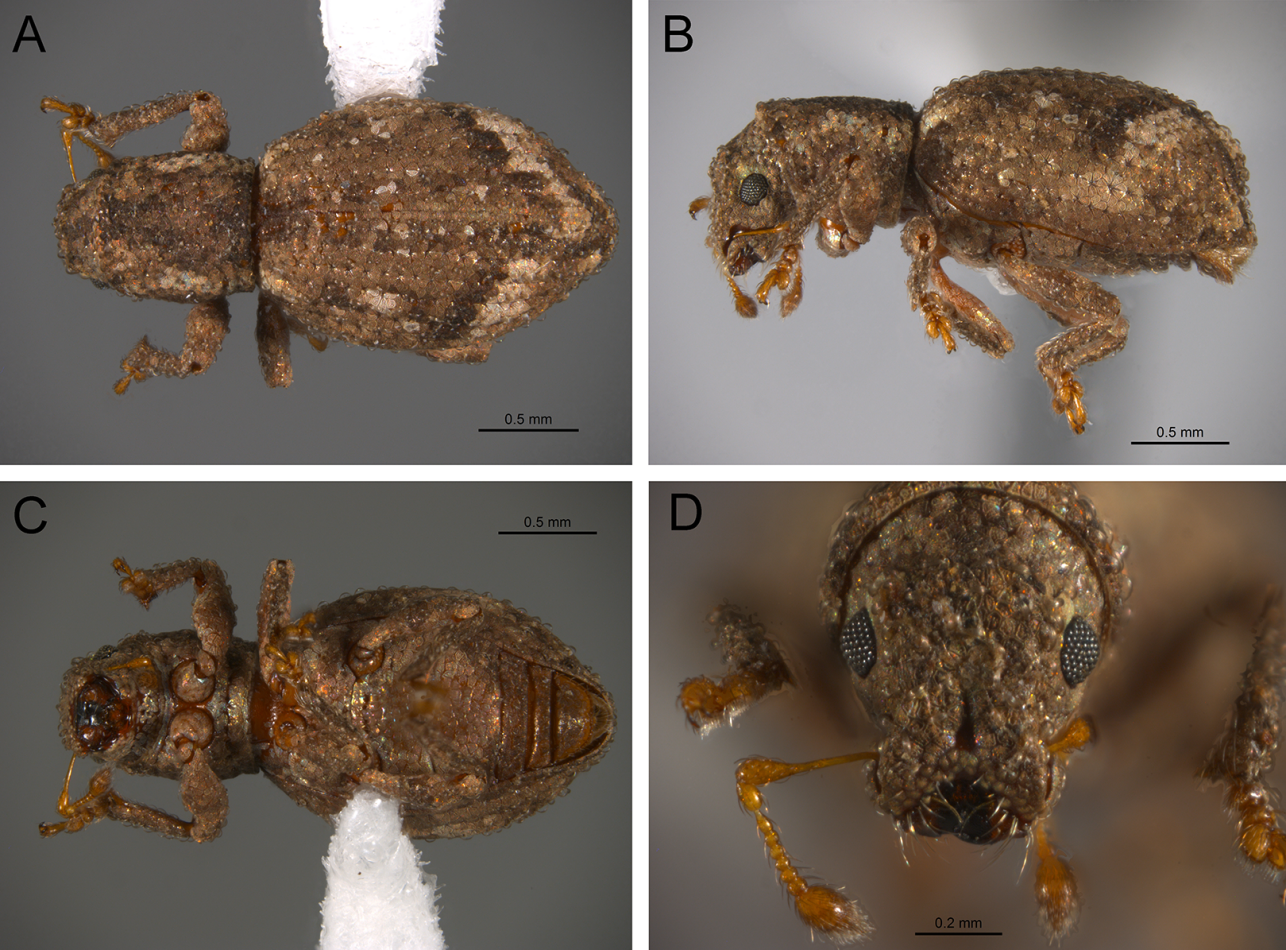
*Isodacrys frontalis* new species. (A) Dorsal habitus; (B) lateral habitus; (C) vental habitus; (D) head in anterior view.

**Figure 19:**
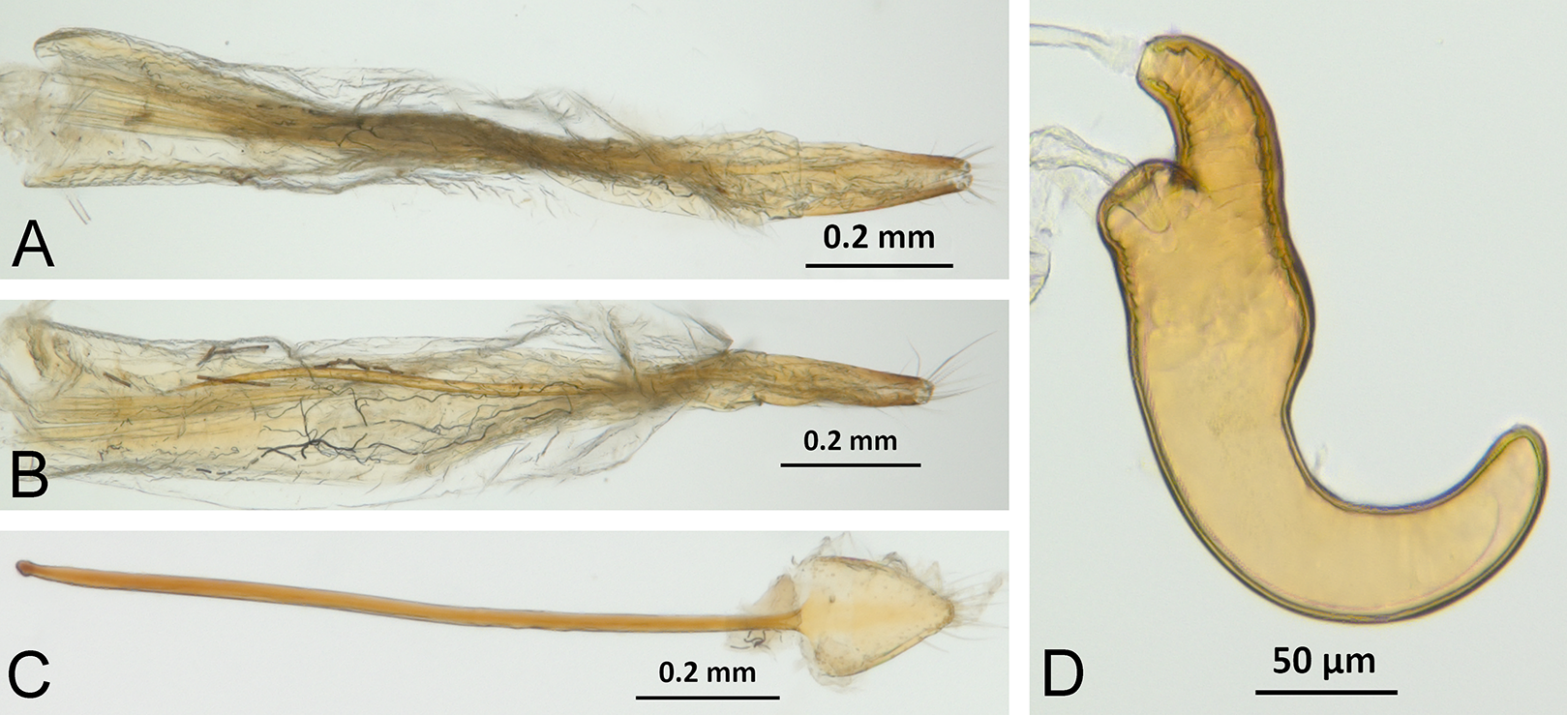
*Isodacrys frontalis* new species, female genitalia. (A) Ovipositor in dorsal view; (B) ovipositor in lateral view; (C) sternite VIII in dorsal view; (D) spermatheca.

***Isodacrys frontalis* Cortés-Hernández, new species**

(Figs. 2, 3F, 6D, 9, 18–19)

urn:lsid:zoobank.org:act:DB24A34B-7EF1-44AD-8DFD-5823FC083899

**Diagnosis.** Setae lanceolate, completely arched to incompletely arched; rostrum with inconspicuous longitudinal sulci mesad of dorsolateral margins, at bent of scrobes; posterior portion of scrobe separated from eye by distance at least twice width of scrobe (Fig. 18B); eyes separated from dorsal outline of frons by distance at least equal to diameter of eye; frons strongly prominent (Fig. 18B); anterolateral margin of prothorax without postocular vibrissae; elytra in dorsal view with five intervals visible at base, intervals seven to nine slightly depressed at base, interval five slightly elevated at base and at fasciae; inner margin of protibiae without teeth.

*Isodacrys frontalis* and *I. brevirostre* have similar scale patterns but the first never has the rostrum as short as *I. brevirostre*. *Isodacrys frontalis* also has the frons very prominent. The shape and disposition of setae are also different: *I. frontalis* has lanceolate, completely arched setae, while *I. brevirostre* presents spatulate setae, from semierect to erect on elytra.

**Description - Habitus.** ♀ 2.2–3.0 mm long, 1.0–1.3 mm wide. Scales polygonal, granulate, mainly overlapping irregularly, from light brown to brown in coloration. Head and rostrum covered with brown scales, with inconspicuous longitudinal vitta of darker scales from occiput to between eyes; scales surrounding eyes, fovea between eyes and sides of rostrum lighter, with metallic reflections. Disc of pronotum with brown scales, with lateral longitudinal vittae of pale scales continuing along base of elytral intervals five and six; sides of prothorax with brown scales. Elytral interval five at basal two fifths with irregular spot of pale scales, disc of elytra mostly with brown scales, at apical 4/5 with two fasciae from interval five towards interval one at summit of apical declivity, anteriormost fascia constituted by dark brown scales, posteriormost fascia by pale brown scales (Fig. 18A). Setae lanceolate, completely arched to incompletely arched, mostly inconspicuous on temple and disc of pronotum. **Rostrum.** Dorsolateral margins of rostrum subparallel, with inconspicuous longitudinal sulci mesad of dorsolateral margins at bent of scrobes; dorsal surface flat, becoming slightly depressed near median sulcus; median sulcus conspicuous or not, both endings marked by foveae (Fig. 18D). Nasal plate with surface shiny, rugulose; anterior margin medially indented; posterior margin V-shaped, carinate. Epistome in continuous plane with remainder of rostrum, slightly depressed, covered by two or three rows of shiny, small scales (Fig. 18D); anterior margin bearing six epistomal setae on each side; posterior margin limiting with anterior fovea of median sulcus (Fig. 18D). Scrobe deep, bent, reaching ventral surface of rostrum; dorsal margin obtusely angled; anterior portion of scrobe about as long as posterior portion; posterior portion of scrobe separated from eye by distance at least twice width of scrobe (Fig. 18B). Antenna setose, without scales; scape capitate; funicular antennomere I clavate, wider and longer than remaining funicular antennomeres; funicular antennomere II clavate, longer than remaining funicular antennomeres; funicular antennomeres III to VII moniliform, distal antennomeres becoming broader (Fig. 18D). **Head.** Head in lateral view with dorsal outline deflexed at frons towards rostrum, frons strongly prominent (Fig. 18B); eyes moderate in size, suboval, prominent, lateral, separated from outline of frons by distance at least equal to diameter of eye. **Prothorax.** Pronotum in dorsal view subcylindrical, nearly as wide as long, lateral outlines slightly sinuate; in lateral view dorsal outline almost straight, anterior and posterior constrictions inconspicuous. Anterolateral margin of prothorax without postocular vibrissae or scales projected anteriorly. **Elytra.** 2.2–2.4 times longer than pronotum, at base as wide as base of prothorax. Basal margin of elytra roundly emarginate. Elytra in dorsal view subelliptical, widest at midlength, five intervals visible at base. Elytra in lateral view with dorsal outline evenly curved towards summit of apical declivity; apical declivity with upper half straight, lower half oblique (Fig. 18B). Elytral interval five slightly elevated at base and at fasciae; intervals seven to nine slightly depressed at base. **Legs.** Procoxae narrowly separated (Fig. 18C), intercoxal process 1/8–1/5 width of procoxa; profemora fusiform, subequal in length and width to metafemora; protibiae straight, as long as metatibiae, inner margin without teeth. Dorsal surface of tarsi setose, first and second tarsomeres with appressed scales or not (probably lost by abrasion). Tarsal claws free, brown at base, becoming black apically. **Abdomen.** Ventrite III with anterior margin narrowly sulcate, sulcus shallow; sulcus of ventrites IV and V progressively wider and deeper (Fig. 18C); sulci enclosed by lateral margins of ventrites; posterior margin of sulci obliterated, ventrites becoming flat posteriorly. **Genitalia.** ♀: Lamina of sternite VIII triangular, apex acute (Fig. 19C); spiculum ventrale 5.5–5.6 times length of lamina. Ovipositor with distal gonocoxites slightly sclerotized laterally, styli represented by single thickened seta; two ventral baculi close to each other, separated by distance similar to width of one baculus; vagina with two pairs of lightly sclerotized proximal rods. Spermatheca fishhook-shaped; corpus wide; collum produced, conical, bent apically to ramus; ramus broadly cylindrical, short; cornu evenly curved, short, not reaching apex of ramus, apically gradually narrowed (Fig. 19D).

**Type material.** Holotype ♀: [MEX.: OAX.; 20 mi S/Juchatengo, 6000′/29.V.1971, S. Peck/Ber207, oak litter] (CMNC). Paratypes (23 ♀): [GUATEMALA: Sacatepéquez/5 km SE Antigua/14.53495–90.69367 ± 50 m/2,150 m, 10.VI.2009/LLAMA Wa-B-08-1-49] [WORLD/WEEVIL/DATABASE/WWD0041418] (1 CMNC); [GUATEMALA: Sacatepéquez/5 km SE Antigua/14.53495–90.69367 ± 50 m/2,150 m, 10.VI.2009/LLAMA Wa-B-08-1-49] [WORLD/WEEVIL/DATABASE/WWD0041419] (1, CMNC); [GUATEMALA: Sacatepéquez/5 km SE Antigua/14.53495–90.69367 ± 50 m/2,150 m, 10.VI.2009/LLAMA Wa-B-08-1-49] [WORLD/WEEVIL/DATABASE/WWD0041420] (1, CMNC); [GUATEMALA: Sacatepéquez/5 km SE Antigua/14.53510–90.69384 ± 50 m/2,150 m, 10.VI.2009/LLAMA Wa-B-08-1-44] [WORLD/WEEVIL/DATABASE/WWD0041311] [*Isodacrys*/GUA 1/det. R.S. Anderson, 20] (1, CMNC); [GUATEMALA: Sacatepéquez/5 km SE Antigua/14.53529–90.69404 ± 50 m/2,150 m, 10.VI.2009/LLAMA Wa-B-08-1-38] [WORLD/WEEVIL/DATABASE/WWD0041144] (1, CMNC); [GUATEMALA: Sacatepéquez/5 km SE Antigua/14.53504–90.69377 ± 50 m/2,150 m, 10.VI.2009/LLAMA Wa-B-08-1-46] [WORLD/WEEVIL/DATABASE/WWD0041358] (1, CMNC); [GUATEMALA: Sacatepéquez/5 km SE Antigua/14.53504–90.69377 ± 50 m/21,50 m, 10.VI.2009/LLAMA Wa-B-08-1-46] [WORLD/WEEVIL/DATABASE/WWD0041359] (1, CMNC); [GUATEMALA: Sacatepéquez/5 km SE Antigua/14.53504–90.69377 ± 50 m/2,150 m, 10.VI.2009/LLAMA Wa-B-08-1-46] [WORLD/WEEVIL/DATABASE/WWD0041360] (1, CMNC); [GUATEMALA: Sacatepéquez/5 km SE Antigua/14.53504–90.69377 ± 50 m/2,150 m, 10.VI.2009/LLAMA Wa-B-08-1-46] [WORLD/WEEVIL/DATABASE/WWD0041361] (1, CMNC); [GUATEMALA: Sacatepéquez/5 km SE Antigua/14.53504–90.69377 ± 50 m/2,150 m, 10.VI.2009/LLAMA Wa-B-08-1-46] [WORLD/WEEVIL/DATABASE/WWD0041363] (1, TAMUIC); [GUATEMALA: Sacatepéquez/5 km SE Antigua/14.53507–90.69380 ± 50 m/2,150 m, 10.VI.2009/LLAMA Wa-B-08-1-45] [WORLD/WEEVIL/DATABASE/WWD0041326] (1, ASUCOB); [GUATEMALA: SACATEPEQUEZ/Guatemala City, Cerro/Alux, 2,260 m., 11.VI.1991/R. Anderson, wet oak/forest, 91-60] (1, CMNC); [GUATEMALA, 9 mi.W./Cd. Guatemala 6900′/VI-7-1974C.W. &L./O’Brien & Marshall] (2, ASUCOB). [MEX.: OAX, same data as holotype] (2, CMNC; 1, MZFC; 1, ICZ); [MEXICO: Oaxaca 3.2 km./S. San Jose del Pacifico/2,440 m, 22.VII.1992, J.S./Ashe,oak-pine for. litt.] (1, CMNC); [MEXICO: Oaxaca, 5.1 km./S. Suchixtepec, 2,150 m/24.VII.1992,92-024, R.S./Anderson, oak|alder|pine/for., leaf litter Berlese] (1, CMNC; 1, MZFC; 1, ICZ); [MEXICO: Oaxaca, 23 km./S.W. Valle Nacional, KM76/1,300 m, 26.VII.1992,92-029/R.S. Anderson, cloud forest/leaf litter Berlese] (1, CMNC).

**Etymology.** Named with the Latin word *frontalis*, referring to its strongly prominent frons.

**Remarks.** Most adults were collected from leaf litter between ~1,830 and 2,440 m above sea level, mainly in various types of oak forests. Males remain unknown.

**Comments.** Specimens of *I*. frontalis were first recognized as an undescribed *Isodacrys* species by R.S. Anderson.

***Isodacrys geminatum* Howden, 1961**

(Figs. 2, 6A, 6C, 9)

*Isodacrys geminatum* Howden, 1961: 80–81. Holotype: ♀, examined; labeled as [Texcoco 7000′/Mexico, Mex./20-VIII-1958] [H.F. Howden/Collector] [HOLOTYPE/*Isodacrys*/*geminatum*/A.T. Howden/No.7384] [CNC/379714]. O’Brien & Wibmer, 1982: 46 (checklist); Morrone, 1999: 145 (checklist); Cortes-Hernández & Morrone, 2019: 50.

**Diagnosis.** Setae of elytra lanceolate, incompletely arched; rostrum with longitudinal sulci mesad of dorsolateral margins of rostrum at bent of scrobes; scrobe right-angled (Fig. 6A), anterior portion as long as posterior portion, posterior portion separated from eye by distance shorter than width of scrobe; anterolateral margin of prothorax without postocular vibrissae (Fig. 6A); elytra in dorsal view obovate, widest before midlength, with five intervals visible at base; prolegs not larger than metalegs (Figs. 6A and 6C), inner margin of protibiae without teeth; and spermatheca U-shaped, corpus subcylindrical, collum produced, conical, long, curved, ramus subcylindrical, very short.

*Isodacrys geminatum* can be separated from *I. antrum* by the elytral setae incompletely arched, the frons slightly deflexed to the rostrum and elytral intervals seven to nine not concave. In *Isodacrys antrum* the elytral setae completely arched, dorsal outline of the head continuously curved towards the rostrum, frons not deflexed and elytral intervals seven to nine concave near base. Additionally, *Isodacrys geminatum* can be easily confused with *I. kuchii*, *I. carlae*, *I. confusum* and *I. fasciatum* because of the strong superficial resemblance among these species with non-denticulate protibiae. *Isodacrys kuchii*, *I. carlae*, *I. confusum* and *I. fasciatum* can be separated from *I. geminatum* by the elytral setae completely arched, the scrobe obtusely angled, anterior portion of the scrobe shorter than posterior portion and posterior portion of the scrobe separated from eye by at least distance equal to width of the scrobe.

**Additional material examined.** MEXICO: Mexico City, Mpio. Xilotepec, Hwy57, 25.VII.2006, oak forest litter, 2,700 m, 20°04′.797′ N, 99°37.906′ W, R.S. Anderson (1, CMNC); Oaxaca, 22 km NE Oaxaca, 18.VI.1979, 2,600 m, H. & A. Howden (1, CMNC); Oaxaca, 3 mi. N Suchixtepec, 4.VI.1971, Ber209, oaklitter, 9500′, S. Peck (1, CMNC); Puebla, 6 mi. NE Zacatepec, 27.VI.1975, L.E. Watrous (9, ASUCOB); Querétaro, Mpio. Colón, Cerro Zamorano, 26.VII.2006, oyamel/oak forest litter, 3,100 m, 20°55.967′ N, 100°11.021′ W, R.S. Anderson (1, CMNC); Querétaro, Mpio. Huimilpan, 1 km NW La Beata, 2.XII.2003, sample #6, leaf litter, 2,500 m, 20° 28.42′ N, 100° 14.49′ W, R. Jones (1, UAQE); Tlaxcala, 6.8 km N Taxco, Hwy 119, 10.VII.1992, ex. misc. Mushrooms, 2,820 m, J.S. Ashe #36 (1, CMNC).

**Remarks.** Except for one adult collected from an unidentified mushroom, most of the examined specimens were collected from leaf litter. This species has been collected between ~2,100 and 3,100 m above sea level. Males remain unknown.

**Comments.** *Isodacrys geminatum* was described based on four specimens from Texcoco, Estado de México, Mexico. The holotype, deposited in CNC, was examined. The three paratypes are deposited in CNC. Identity of additional material examined was based on comparisons with the holotype and by agreement with the original species description.

***Isodacrys guatemalenum*** Sharp, 1911

(Figs. 1, 5D, 9)

*Isodacrys guatemalenus* Sharp, 1911: 175. Syntypes not examined. Howden, 1993a: 1; Alonso-Zarazaga & Lyal, 1999: 179.

*Isodacrys guatemalensis*; Pierce, 1913: 401 (designation as type species of *Isodacrys*).

*Isodacrys guatemalena*; Blackwelder, 1947: 799 (checklist).

*Isodacrys guatemalenum*; Howden, 1961: 83–84 (redescription); O’Brien & Wibmer, 1982: 46 (checklist); Morrone, 1999: 145 (checklist).

**Diagnosis.** Setae of elytra parallel-sided, very long, erect (Fig. 5D); epistome with longitudinal carina joined with posterior margin of nasal plate (e.g., Fig. 4C); scrobe with anterior portion as long as posterior portion; anterolateral margin of prothorax without postocular vibrissae; elytra in dorsal view subelliptical, widest at midlength (Fig. 5D), with seven intervals visible at base; profemora wider than metafemora, protibiae longer than metatibiae, inner margin of protibiae with teeth; median lobe stout, shorter than spiculum gastrale, slightly longer than temones, apex in dorsal view acute, manubrium as long as median lobe; and spermatheca u-shaped, corpus subcylindrical, collum and ramus inconspicuous.

*Isodacrys guatemalenum* is separated from *I. ellipticum* by its parallel-sided long elytral setae, conspicuously erect, and by the spermatheca with collum and ramus inconspicuous. In *I. ellipticum* the elytral setae are spatulate, from semierect to erect, and the spermatheca with collum inconspicuous, ramus short.

**Material examined.** GUATEMALA: Sacatepéquez, Antigua, 16.VIII.1947, 5000′, C. & P. Vaurie (1, CMNC); Sacatepéquez, Santa Lucía Milpas Altas, 24.VI.1993, 1,800 m, B.D. Gill (1, CMNC). HONDURAS: Choluteca, 1 km W San Marcos de Colón, 23.VII.1977, C. & L. O’Brien, G. Marshall (12, ASUCOB); Comayagua, Comayagua, 18.VII.1977, J.V. Mankins (2, CMNC); Comayagua, 22 km N Comayagua, 18.VII.1977, C. & L. O’Brien, G. Marshall (80, ASUCOB; 12, CASENT).

**Remarks.** Some specimens have been collected between ~1,500 and 1,800 m above sea level.

**Comments.** *Isodacrys guatemalenum* was described based on 15 specimens from Dueñas, Sacatepéquez, Guatemala. The syntypes are distributed in BMNH and USNM and were not examined. Identity of material examined was based on comparison with specimens identified by Anne Howden and by agreement with the original species description (Sharp, 1911) and redescription (Howden, 1961).

From the males examined, none of them possess postocular vibrissae. Nonetheless, Howden (1961) stated that postocular vibrissae can be poorly developed in males.

**Figure 20:**
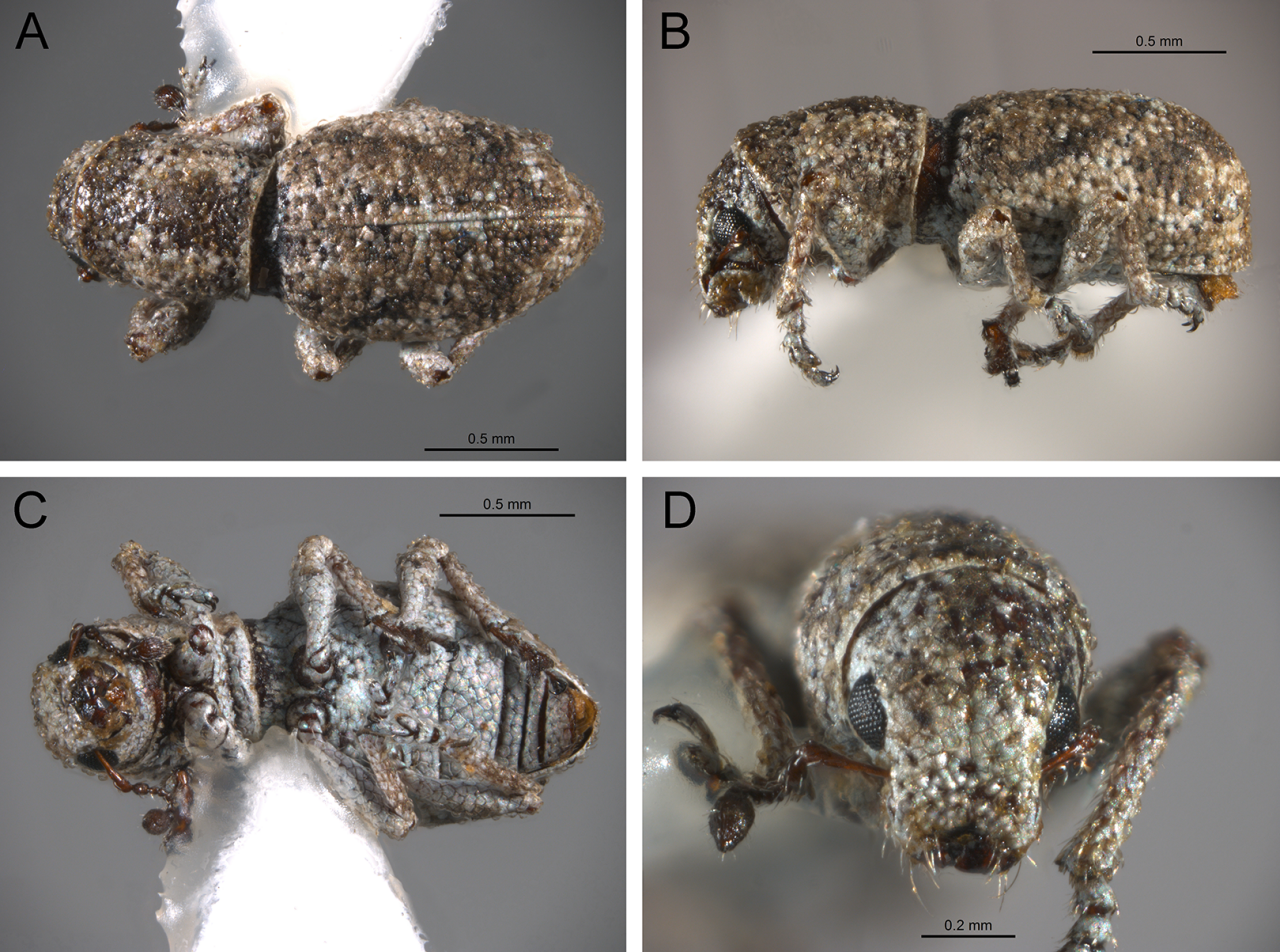
*Isodacrys kuchii* new species. (A) Dorsal habitus; (B) lateral habitus; (C) ventral habitus; (D) head in anterior view.

**Figure 21:**
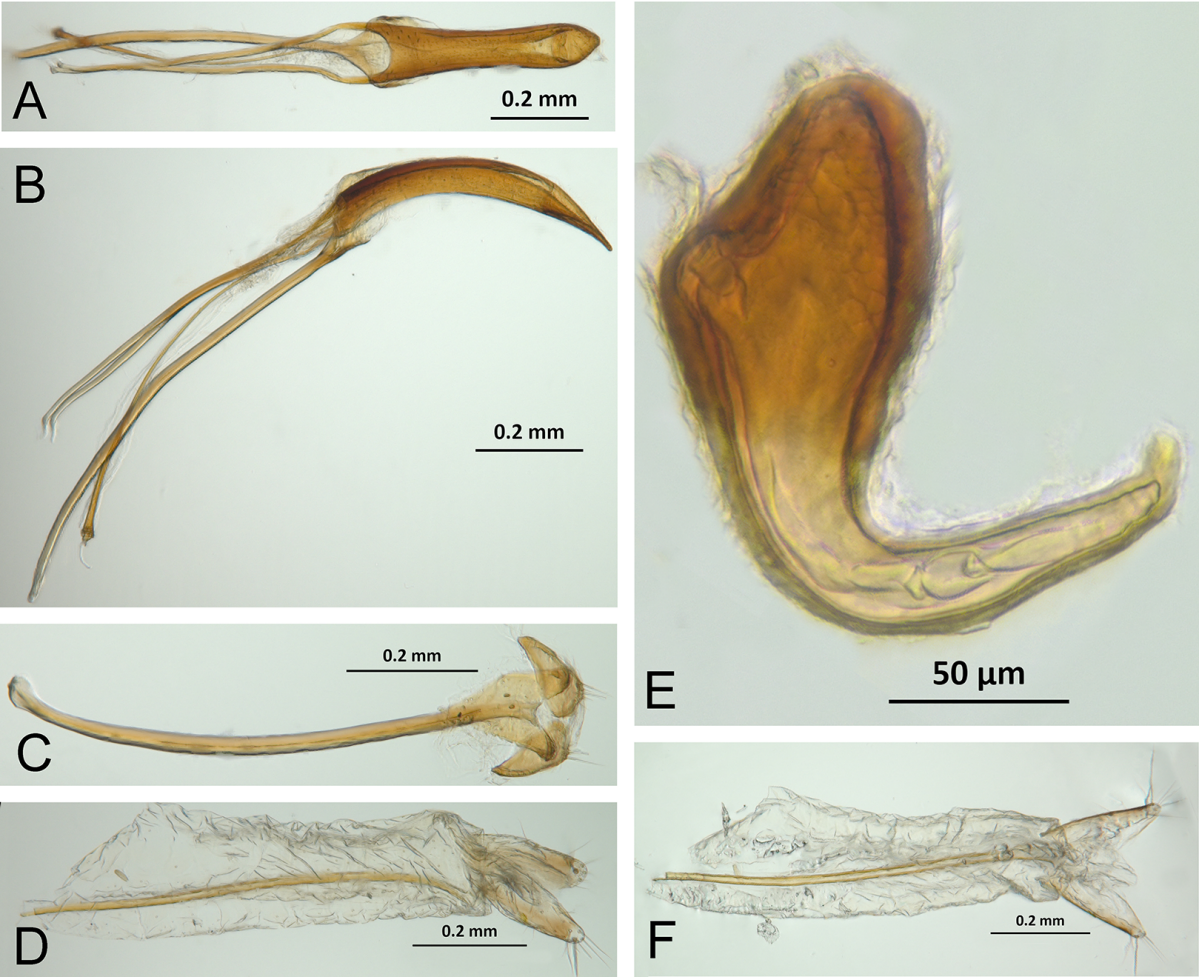
*Isodacrys kuchii* new species, genitalia. (A) Median lobe and tegmen in dorsal view; (B) median lobe and tegmen in lateral view; (C) male hemisternites VIII and sternite IX in dorsal view; (D) ovipositor in lateral view; (E) spermatheca; (F) ovipositor in dorsal view.

***Isodacrys kuchii* Cortés-Hernández, new species**

(Figs. 1, 7B–7D, 9, 20–21)

urn:lsid:zoobank.org:act:CB805B2D-80BA-4B36-9B6D-90740B222917

**Diagnosis.** Setae lanceolate, completely arched, more inconspicuous on head; posterior portion of scrobe separated from eye by distance equal to width of scrobe; antennal scape without scales (Fig. 20D); eyes large; anterolateral margin of prothorax without postocular vibrissae (Fig. 20B), pronotum covered with brown scales medially (Fig. 20A), with broad, lateral longitudinal vittae of white scales; profemora 1.2 times wider than metafemora, inner margin of protibiae without teeth, all tarsomeres with appressed scales (Fig. 20B); male terminalia with median lobe shorter than spiculum gastrale and manubrium (Fig. 21B); spermatheca comma-shaped, collum produced, conical, not directed to ramus (Fig. 21E).

*Isodacrys kuchii* can be confused with *I. carlae*, *I. confusum* and *I. fasciatum*. *Isodacrys kuchii* can be easily separated from those species by its inconspicuous median sulcus, with both endings marked by foveae, and the posterior portion of the scrobe separated from eye by distance equal to width of the scrobe. In *I. carlae*, *I. confusum* and *I. fasciatum* the median sulcus is conspicuous, usually deep, both endings without fovea, and posterior portion of the scrobe separated from eye by distance greater than width of the scrobe.

**Description - Habitus.** ♂ 2.1–2.2 mm long, 0.8–0.9 mm wide; ♀ 2.4 mm long, 1.1 mm wide. Scales polygonal, granulate, mainly overlapping irregularly, from brown to white in coloration. Head from vertex to frons covered by brown and white scales; rostrum and genae covered with white scales. Disc of pronotum covered with brown scales medially (Fig. 20A), with broad, lateral longitudinal vittae composed of white scales; sides of prothorax with white scales, with irregular longitudinal brown vittae between anterior and posterior constrictions of prothorax. Disc of elytra mostly with brown scales, intervals six to three at second third with irregular white fasciae; epipleura mostly with white scales, intervals one to four irregularly spotted with white scales at summit of apical declivity. Setae lanceolate, completely arched, more inconspicuous on head. **Rostrum.** Dorsolateral margins of rostrum subparallel; dorsal surface flat, becoming slightly depressed near median sulcus; median sulcus inconspicuous, both endings marked by foveae (Fig. 20D). Nasal plate with surface rugulose; anterior margin medially indented; posterior margin V-shaped, carinate. Epistome in continuous plane with remainder of rostrum, covered by three or four rows of shiny, smaller scales (Fig. 20D); anterior margin bearing three or four epistomal setae on each side; posterior margin limiting with anterior fovea of median sulcus. Scrobe deep, bent, reaching ventral surface of rostrum; dorsal margin obtusely angled; anterior portion of scrobe about 2/3 length of posterior portion; posterior portion of scrobe separated from eye by distance equal to width of scrobe. Antenna setose, without scales (Fig. 20D); scape capitate; funicular I antennomere clavate, wider and longer than remaining funicular antennomeres; funicular antennomere II clavate; funicular antennomeres III to VII moniliform, distal antennomeres becoming broader. **Head.** Head in lateral view with dorsal outline deflexed at frons towards rostrum; eyes large, subcircular, prominent, lateral, separated from outline of frons by distance about half diameter of eye. **Prothorax.** Pronotum in dorsal view subcylindrical, 1.1–1.2 times longer than wide, lateral outlines sinuate; in lateral view dorsal outline sinuate, anterior and posterior constrictions conspicuous. Anterolateral margin of prothorax without postocular vibrissae (Fig. 20B), with row of scales slightly projected anteriorly. **Elytra.** 1.6–1.8 times longer than pronotum, at base as wide as base of prothorax. Basal margin of elytra roundly emarginate. Elytra in dorsal view obovate, widest before midlength, five intervals visible at base. Elytra in lateral view with dorsal outline gently evenly curved towards summit of apical declivity; apical declivity with upper two thirds straight, lower third oblique. Elytral intervals with no elevations or depressions. **Legs.** Procoxae narrowly separated (Fig. 20C), intercoxal process 1/5–1/7 width of procoxa; profemora fusiform, subequal in length to metafemora, 1.2 times wider than metafemora; protibiae straight, as long as metatibiae, inner margin without teeth. Dorsal surface of tarsi setose, all tarsomeres with appressed scales. Tarsal claws free. **Abdomen.** Ventrite III with anterior margin narrowly sulcate, sulcus shallow; sulcus of ventrites IV and V progressively wider and deeper (Fig. 20C); sulci enclosed by lateral margins of ventrites; posterior margin of sulci obliterated, ventrites becoming flat posteriorly. **Genitalia.** ♂: hemisternites VIII teardrop-shaped, outer corner not truncate (Fig. 21C); basal plate of sternite IX lightly sclerotized, distally bifurcate, tips of bifurcations sclerotized (Fig. 21C); spiculum gastrale 1.3 times longer than median lobe, distally continuing through basal plate; ring of tegmen without parameres; manubrium 1.6 times longer than median lobe (Fig. 21B); temones as long as median lobe (Fig. 21B); median lobe 4.4 times longer than wide in dorsal view, basal half more curved in lateral view, apical opening elliptical, base of opening prolonged anteriorly, apex acute (Fig. 21A). ♀: Lamina of sternite VIII triangular, apex acute. Ovipositor with distal gonocoxites slightly sclerotized laterally, styli represented by single thickened seta; two ventral baculi close to each other, separated by distance similar to width of one baculus. Spermatheca comma-shaped; corpus wide, narrowing towards cornu; collum produced, conical, not directed to ramus; ramus slightly tumid; cornu strongly bent near corpus, then straight, slightly sinuate, apically gradually narrowed (Fig. 21E). **Sexual dimorphism.** Ventrites I and II slightly concave mesally in males, convex in females.

**Type material.** Holotype ♂: [MEXICO: Puebla/Mpio: Zapotitlán Salinas/1 km N San Juan Raya] [18° 19′ 11″, 97° 36′ 50″/19-vii-2011, A. Obregón/1, 755 m, Matorral] [Isodacrys/geminatum/Howden 1961/Det. Jesús Luna Cozar 2015] (CNIN). Paratypes (1 ♂, 1 ♀): [MEXICO: Puebla/same data as holotype] [18° 19′ 11″, 97° 36′ 50″/same data as holotype] [Isodacrys/same data as holotype] (1 ♂, CNIN); [MEXICO: Puebla/6 Mi. SW. Tehacan/July 8, 1973/Schaffner & Mastro] [TAMU-ENTO/X0725504] [*Isodacrys*/*?geminatum*/DET./A. Howden] (1 ♀, TAMUIC).

**Etymology.** This species is named with the word kuchīí meaning weevil in Popoloca, one of the native languages in the Tehuacán valley, Puebla, where the specimens were collected.

**Remarks.** The type series was collected in the Tehuacán valley, in areas predominantly covered with xerophytic vegetation. The holotype and one of the paratypes were collected at 1,755 m above sea level.

**Comments.** Part of the female genitalia was lost during dissection, that is why some characters of the sternite VIII were not observed, described or coded for the phylogenetic analysis.

***Isodacrys mexicanum*** Sharp, 1911

(Figs. 1, 6E, 9)

*Isodacrys mexicanus* Sharp, 1911: 176. Syntypes not examined.

*Isodacrys mexicana*; Blackwelder, 1947: 799 (checklist).

*Isodacrys mexicanum*; Howden, 1961: 92–93 (redescription); O’Brien & Wibmer, 1982: 46 (checklist); Morrone, 1999: 145 (checklist); Cortes-Hernández & Morrone, 2019: 50.

**Diagnosis.** Setae lanceolate, small, completely arched, mostly inconspicuous on disc of pronotum; scrobe with anterior portion as long as posterior portion, posterior portion separated from eye by distance nearly 1.2 times width of scrobe; eyes large, subcircular, convex; pronotum nearly 1.2 times longer than wide; prothorax strongly sinuate between anterior and posterior constrictions, anterolateral margin of prothorax with one to five vestigial postocular vibrissae; elytra in dorsal view obovate, widest before midlength, with seven intervals visible at base, intervals three to five slightly elevated at base; profemora wider than metafemora, protibiae longer than metatibiae, inner margin of protibiae with teeth; median lobe nearly as long as spiculum gastrale, apex in lateral view bent, apex in dorsal view broadly rounded; spermatheca comma-shaped, corpus slightly subglobose, collum slightly produced, subconical, directed to ramus, ramus broadly subcylindrical, short.

*Isodacrys mexicanum* can be separated from *I. apicale* by setae lanceolate, small, completely arched, the pronotum nearly 1.2 times longer than wide, the postocular vibrissae reduced in number and size and apex of the elytra conjointly rounded. In *Isodacrys apicale* setae of the elytra are spatulate, incompletely arched, erect on declivity, the pronotum as wide as long, more than six postocular vibrissae, some well-developed, and apex of the elytra bisinuate.

**Material examined.** MEXICO: Aguascalientes, 24 miles West of Aguascalientes, 5.VI.1956, B. Banta (1, CASENT); Mexico City, Milpa Alta, 26.VII.1980, H. Brailovsky (1, CNIN); Mexico City, Pedregal de San Ángel, Loc. 21, 22.VIII.1961 (2, CNIN); Mexico City, Zumpango, 18.VII.1981, Peter Jump (1, CMNC; 1, TAMUIC); Michoacán, km 154 car. Irapuato-Guadalajara, 26.VII.1984, M. García (1, CNIN); Morelos, Tepoztlán, 11.X.1968, R. Muñíz V. (1, CMNC; 1, ASUCOB); Morelos, YMCA camp. Tepoztlán, 21.VIII.1958, H.F. Howden (4, CMNC); Oaxaca, 1.1 mi. W El Tule, 87/048, 5400′, Woolley, Zolnerowich (2, TAMUIC).

**Remarks.** Adults have been collected beating trees at elevations of ~1,500 m above sea level (Howden, 1961).

**Comments.** *Isodacrys mexicanum* was described based on five specimens from Guanajuato, Mexico. The syntypes, distributed in BMNH and USNM, were not examined. Identity of material examined was corroborated by comparing with specimens identified by Anne Howden and by agreement with the original species description (Sharp, 1911) and redescription (Howden, 1961).

***Isodacrys obrienorum* Cortés-Hernández, new species**

(Figs. 9, 22–23)

**Figure 22:**
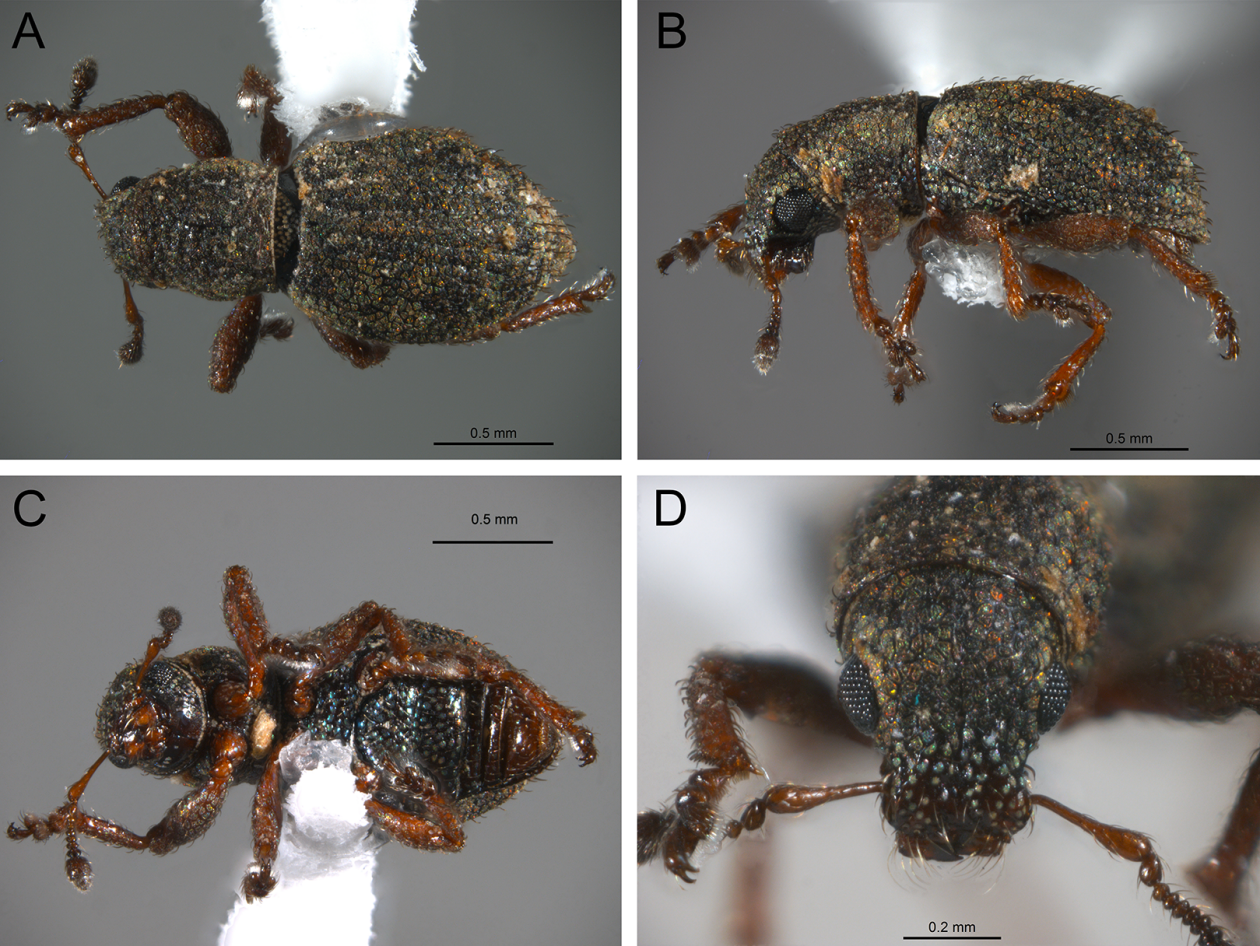
*Isodacrys obrienorum* new species. (A) Dorsal habitus; (B) lateral habitus; (C) ventral habitus; (D) head in anterior view.

urn:lsid:zoobank.org:act:9DB9D7AA-F4AB-4424-A810-3DD5BF6ABA56

**Figure 23:**
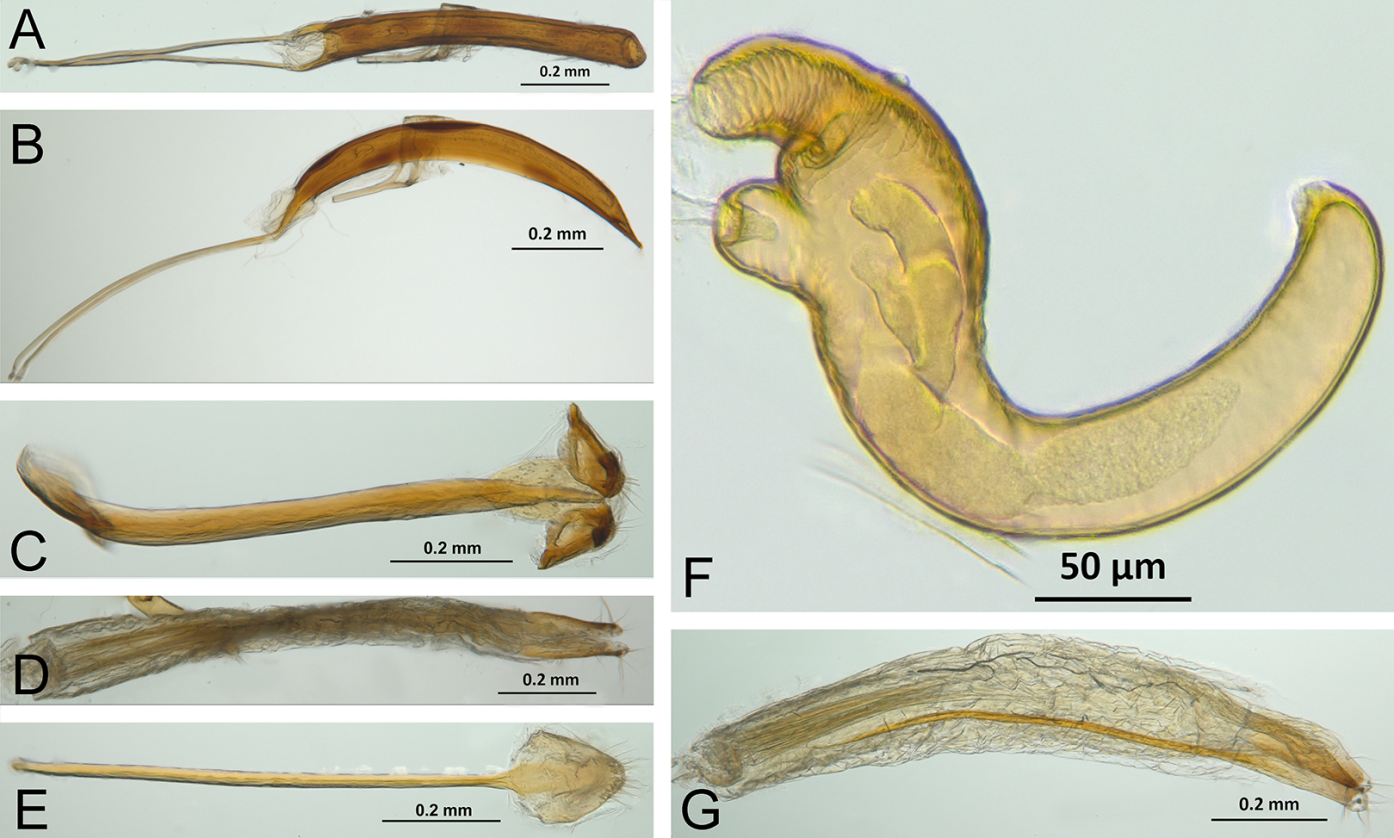
*Isodacrys obrienorum* new species, genitalia. (A) Median lobe in dorsal view; (B) median lobe in lateral view; (C) male hemisternites VIII and sternite IX in dorsal view; (D) ovipositor in dorsal view; (E) female sternite VIII in dorsal view; (F) spermatheca; (G) ovipositor in lateral view.

**Diagnosis.** Scales mostly contiguous and not overlapping (Fig. 22A); setae long, mostly erect (Fig. 22B); rostrum with short longitudinal sulci mesad of dorsolateral margins at bent of scrobes; epistome with scattered scales, integument exposed (Fig. 22D); scrobe with dorsal margin right-angled, posterior portion separated from eye by distance shorter than width of scrobe; eyes large (Fig. 22B), prominent; inner margin of protibiae without teeth; manubrium longer than median lobe; lamina of female sternite VIII with apex blunt (Fig. 23E).

From its closest relative, *I. antrum*, *I. obrienorum* can be recognized by the inner margin of the protibiae smooth, without teeth, scales not overlapping and setae semierect to erect. In *I. antrum* the protibiae possess teeth along their inner margin, scales overlapping irregularly, and setae completely arched.

**Description - Habitus.** ♂ 1.9–2.0 mm long, 0.8–0.9 mm wide; ♀ 2.0–2.2 mm long, 0.9–1.1 mm wide. Scales polygonal, with raised margins, granulate, mostly contiguous and not overlapping, from dark brown to brown in coloration (Fig. 22A). Head and rostrum covered with brown scales. Disc of pronotum with dark brown to brown scales, with lateral broad vittae of brown to pale brown scales continuing with base of elytral intervals six and seven; sides of prothorax with dark brown scales. Elytra with irregular scale pattern. Setae lanceolate, long, slightly wide apically, from incompletely arched to erect, mostly erect (Fig. 22B). **Rostrum.** Dorsolateral margins of rostrum subparallel, with short longitudinal sulci mesad of dorsolateral margins at bent of scrobes; dorsal surface flat; median sulcus deeply impressed (Fig. 22D), both endings marked by foveae. Nasal plate with surface shiny, rugulose; anterior margin medially indented; posterior margin V-shaped, carinate. Epistome in continuous plane with remainder of rostrum, covered by shiny, small and scattered scales, integument of epistome exposed (Fig. 22D); anterior margin bearing four epistomal setae on each side; posterior margin limiting with anterior fovea of median sulcus. Scrobe deep, bent, reaching ventral surface of rostrum; dorsal margin right-angled; anterior portion of scrobe 2/3 length of posterior portion; posterior portion of scrobe separated from eye by distance shorter than width of scrobe. Antenna setose, without scales; scape capitate; funicular antennomere I clavate, wider and longer than remaining funicular antennomeres; funicular antennomere II clavate; funicular antennomeres III to VII moniliform, distal antennomeres becoming broader (Fig. 22D). **Head.** In lateral view with dorsal outline slightly deflexed at frons towards rostrum, frons not prominent (Fig. 22B); eyes large, subcircular (Fig. 22B), prominent, lateral, separated from outline of frons by less than half diameter of eye. **Prothorax.** Pronotum in dorsal view subcylindrical, as wide as long, lateral outlines sinuate (Fig. 22A); in lateral view dorsal outline sinuate between anterior and posterior constrictions. Anterolateral margin of prothorax with one or two rudimentary, small postocular vibrissae or lacking. **Elytra.** 1.9–2.1 times longer than pronotum, at base as wide as base of prothorax. Basal margin of elytra roundly emarginate. Elytra in dorsal view obovate, widest before midlength (Fig. 22A); five intervals visible at base. Elytra in lateral view with dorsal outline evenly curved towards summit of apical declivity; apical declivity oblique, subtle (Fig. 22B). Elytral intervals with no elevations or depressions. **Legs.** Procoxae narrowly separated (Fig. 22C), intercoxal process 1/6–1/5 width of procoxa; profemora fusiform, subequal in length and width to metafemora; protibiae straight, as long as metatibiae, inner margin without teeth. Dorsal surface of tarsi setose, without scales. Tarsal claws free. **Abdomen.** Ventrite III with anterior margin narrowly sulcate; sulcus of ventrites IV and V progressively wider and deeper (Fig. 22C); sulci enclosed by lateral margins of ventrites; posterior margin of sulci obliterated, ventrites becoming flat posteriorly. **Genitalia.** ♂: Hemisternites VIII teardrop-shaped, outer corner not truncate (Fig. 23C); basal plate of sternite IX lightly sclerotized, distally bifurcate, tips of bifurcations sclerotized; spiculum gastrale as long as median lobe, distally continuing through basal plate; ring of tegmen without parameres; manubrium 1.5 times longer than median lobe; temones 4/5 length of median lobe; median lobe 8.9 times longer than wide in dorsal view, evenly curved in lateral view (Fig. 23B), apical opening elliptical, apex acute. ♀: lamina of sternite VIII triangular, apex blunt; spiculum ventrale 4.3 times length of lamina. Ovipositor with distal gonocoxites slightly sclerotized laterally, styli represented by single thickened seta; two ventral baculi close to each other, separated by distance similar to width of one baculus; vagina with two pairs of lightly sclerotized proximal rods (Fig. 23G). Spermatheca comma-shaped; corpus slightly subglobose, narrowing towards cornu; collum produced, conical, bent apically towards ramus; ramus broadly cylindrical, short; cornu strongly curved near corpus, then gently curved, elongate, reaching apex of ramus, apically gradually narrowed (Fig. 23F). **Sexual dimorphism.** Ventrites I and II slightly concave mesally in males, convex in females; apex of ventrite V truncate in males, rounded in females.

**Type material.** Holotype ♂: [GUATEMALA, 12 mi.SE./San Cristobal de To-/tonicapan, 9800′/VII-28.1974] [under stones] [C.W. & L.B. O’Brien/& G.B.Marshall] (ASUCOB). Paratypes (6 ♂♂, 6 ♀♀): [GUATELAMA: Jalapa/4 km E Mataquescuintla/14.52705–90.14671 ± 55 m/2,660 m, 2.VI.2009,/LLAMA #Go-B-07-1-04/cloud forest beating vegetation] [WORLD/WEEVIL/DATABASE/WWD0125181] (1 ♀, CMNC); [GUATELAMA: Jalapa/4 km E Mataquescuintla/14.52705–90.14671 ± 55 m/2,660 m, 2.VI.2009,/LLAMA #Go-B-07-1-04/cloud forest beating vegetation] [WORLD/WEEVIL/DATABASE/WWD0125188] (1 ♂, CMNC); [GUATELAMA: Jalapa/4 km E Mataquescuintla/14.52705–90.14671 ± 55 m/2,660 m, 2.VI.2009,/LLAMA #Go-B-07-1-04/cloud forest beating vegetation] [WORLD/WEEVIL/DATABASE/WWD0125177] (1 ♂, CMNC); [GUATEMALA, 12 mi.SE./San Cristobal deTo-/tonicapan, same data as holotype] (2 ♂♂, 3 ♀♀, ASUCOB; 1 ♂, 1 ♀, MZFC); [GUATEMALA, 10 mi.NW./SanCristobal de/Totonicapan,9300′] [VII-29-1974C.W.&L./O’Brien & Marshall] [under stones/and dead/grass clumps] (1 ♀, ASUCOB); [GUATEMALA: San Marcos/above Sibinal/3,009 m, 15.14366–92.06662/8 Jun 2015, R. Anderson/alder litter, 2015-127] (1 ♂, CMNC).

**Etymology.** Named after Charlie and Lois O’Brien, who collected most of the type series, in honor of their many years of world-wide collecting and their huge contributions to weevil knowledge.

**Remarks.** Adults have been collected under rocks, on dead grass clumps, on *Alnus* sp. (Betulaceae) litter, and beating vegetation, between 2,660 and 3,009 m above sea level. Some collected in cloud forests.

**Comments.** It was first recognized as an undescribed *Isodacrys* species by Charles W. O’Brien.

***Isodacrys okuiltontli* Cortés-Hernández, new species**

(Figs. 9, 24–25)

urn:lsid:zoobank.org:act:01FC3BDC-D6AC-48D1-BF6A-60CF65BB6626

**Figure 24:**
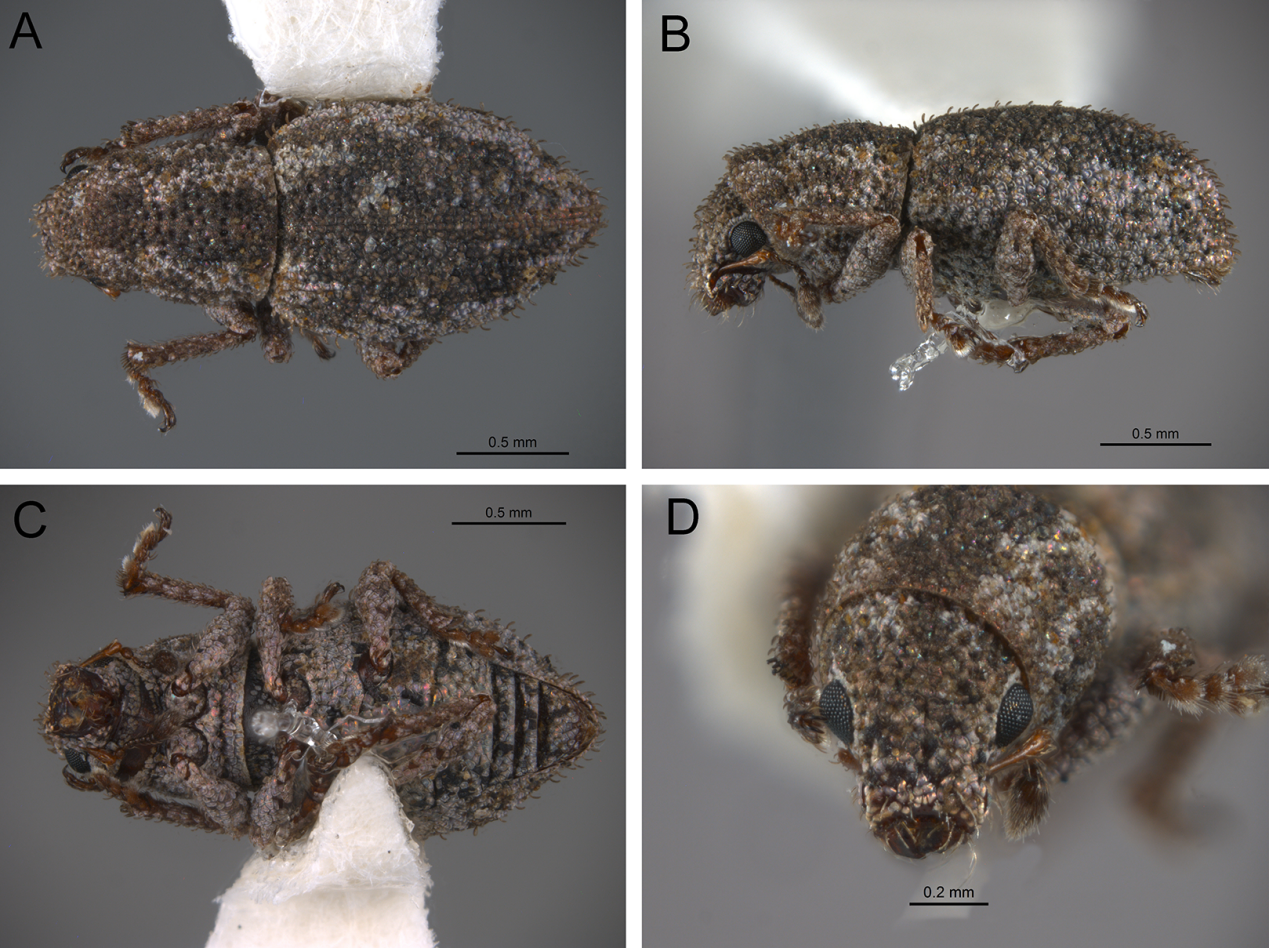
*Isodacrys okuiltontli* new species. (A) Dorsal habitus; (B) lateral habitus; (C) ventral habitus; (D) head in anterior view.

**Diagnosis.** Setae short, spatulate, erect (Fig. 24B); scrobe with anterior portion as long as posterior portion, posterior portion of scrobe separated from eye by distance shorter than width of scrobe; eyes large, subcircular; anterolateral margin of prothorax without postocular vibrissae (Fig. 24B); protibiae with 2 or 3 small teeth.

*Isodacrys okuiltontli* can be readily separated from *I. apicale* by its short and erect setae, the posterior portion of the scrobe separated from eye by distance shorter than width of the scrobe, postocular vibrissae absent and apical margin of the elytra conjointly rounded. In *I. apicale* setae are longer, incompletely arched or becoming erect towards the summit of apical declivity, posterior portion of the scrobe separated from eye by distance greater than width of the scrobe, the prothorax with well-developed postocular vibrissae and apical margins of the elytra bisinuate.

**Figure 25:**
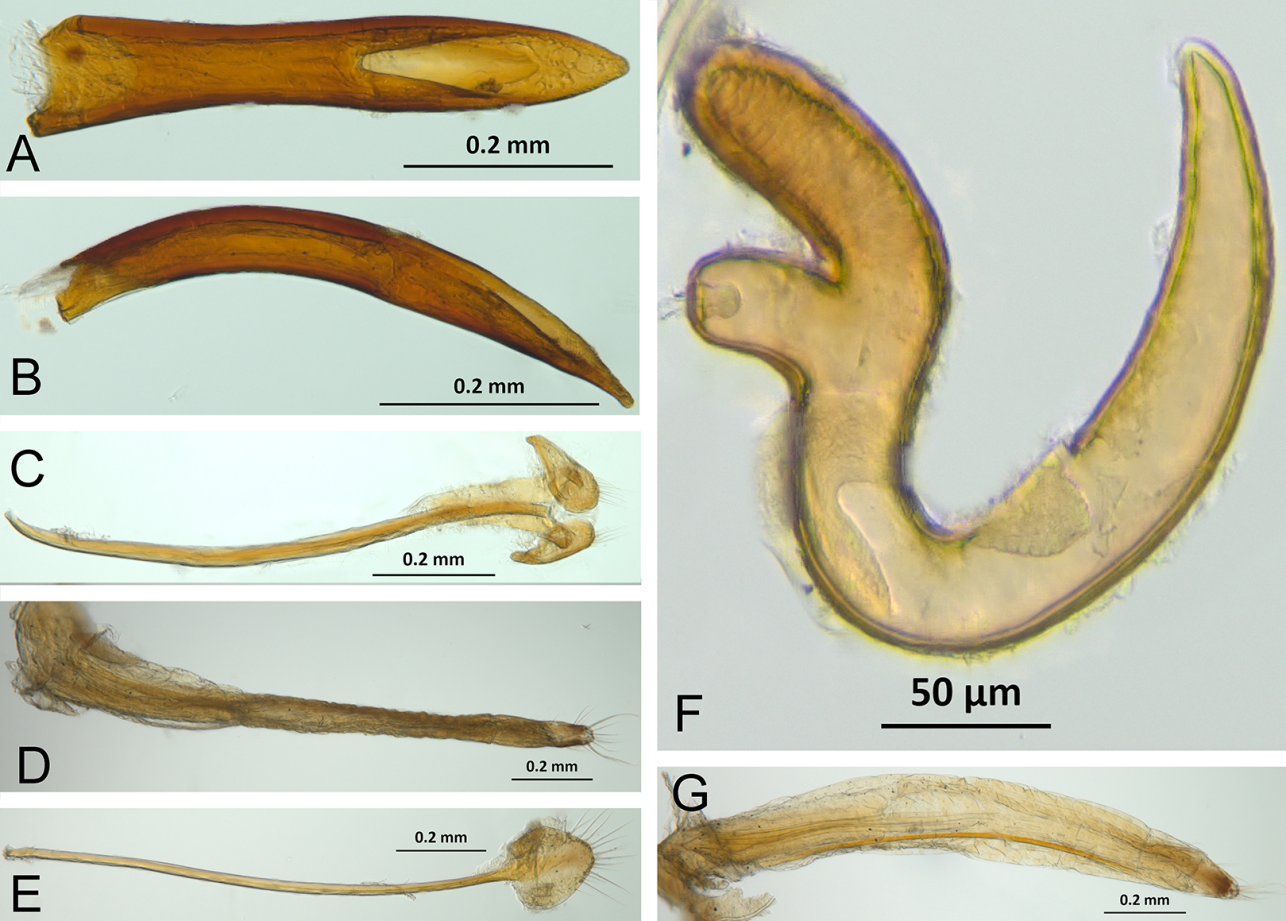
*Isodacrys okuiltontli* new species, genitalia. (A) Median lobe in dorsal view; (B) median lobe in lateral view; (C) male hemisternites VIII and sternite IX in dorsal view; (D) ovipositor in dorsal view; (E) female sternite VIII in dorsal view; (F) spermatheca; (G) ovipositor in lateral view.

**Description - Habitus.** ♂ 2.2 mm long, 0.9 mm wide; ♀ 2.4–2.6 mm long, 1.0 mm wide. Scales polygonal, granulate, mainly overlapping irregularly, from brown to gray in coloration. Head with three longitudinal brown vittae at vertex, converging at frons; genae and rostrum mainly covered with gray scales. Longitudinal brown vittae of head continuing along disc of pronotum, wider, the lateral ones irregular; sides of prothorax irregularly scattered with brown and white scales. Disc of elytra with apparent irregular scale color pattern, epipleura mostly with white scales. Setae short, spatulate, erect (Fig. 24B); mostly inconspicuous on temple. **Rostrum.** Dorsolateral margins of rostrum subparallel; dorsal surface flat; median sulcus conspicuous or not, anterior end marked by fovea (Fig. 24D). Nasal plate with surface shiny, rugulose; anterior margin medially indented; posterior margin V-shaped, carinate. Epistome in continuous plane with remainder of rostrum, covered by three rows of shiny, smaller scales (Fig. 24D); anterior margin bearing three epistomal setae on each side; posterior margin limiting with anterior fovea of median sulcus. Scrobe deep, bent, reaching ventral surface of rostrum; dorsal margin obtusely angled; anterior portion of scrobe as long as posterior portion; posterior portion of scrobe separated from eye by distance shorter than width of scrobe. Antenna setose, without scales; scape capitate; funicular antennomere I clavate, wider and longer than remaining funicular antennomeres; funicular antennomere II clavate, longer than remaining funicular antennomeres; funicular antennomeres III to VII moniliform, distal antennomeres becoming broader. **Head.** In lateral view with dorsal outline gently deflexed at frons towards rostrum, frons not prominent; eyes large, subcircular (Fig. 24B), prominent, lateral, separated from outline of frons by near 1/2 diameter of eye. **Prothorax.** Pronotum in dorsal view subcylindrical, 1.1–1.2 times longer than wide, lateral outlines sinuate; in lateral view dorsal outline sinuate, anterior and posterior constrictions conspicuous. Anterolateral margin of prothorax without postocular vibrissae or scales projected anteriorly (Fig. 24B). **Elytra.** 1.6–1.7 times longer than pronotum, at base as wide as base of prothorax. Basal margin of elytra roundly emarginate. Elytra in dorsal view obovate, widest before midlength (Fig. 24A), seven intervals visible at base. Elytra in lateral view with dorsal outline evenly curved towards summit of apical declivity; apical declivity oblique (Fig. 24B). Elytral intervals three and five inconspicuously elevated before midlength. **Legs.** Procoxae narrowly separated, intercoxal process 1/6–1/5 width of procoxa; profemora fusiform, subequal in length and width to metafemora; protibiae straight, as long as metatibiae, inner margin sinuate, with two or three small teeth. Dorsal surface of tarsi setose, first and second tarsomere with appressed scales. Tarsal claws free. **Abdomen.** Ventrites III, IV and V with anterior margin narrowly sulcate, progressively wider (Fig. 24C); sulci enclosed by lateral margins of ventrites; posterior margin of sulci obliterated, ventrites becoming flat posteriorly. **Genitalia.** ♂: Hemisternites VIII teardrop-shaped, outer corner not truncate (Fig. 25C); basal plate of sternite IX lightly sclerotized, distally bifurcate, tips of bifurcations sclerotized (Fig. 25C); spiculum gastrale ~1.5 times longer than median lobe, distally continuing through basal plate; median lobe ~7 times longer than wide in dorsal view, basal half more curved in lateral view, apical opening elliptical, base of opening prolonged anteriorly, apex acute (Fig. 25A). ♀: Lamina of sternite VIII triangular, apex blunt (Fig. 25E); spiculum ventrale 5.3 times length of lamina. Ovipositor with distal gonocoxites slightly sclerotized laterally, styli represented by single thickened seta; two ventral baculi close to each other, separated by distance similar to width of one baculus; vagina with two pairs of lightly sclerotized proximal rods (Fig. 25G). Spermatheca u-shaped; corpus cylindrical; collum produced, cylindrical, curved; ramus cylindrical, shorter than collum; cornu strongly curved near corpus, then evenly curved, elongate, reaching apex of collum, apically gradually narrowed (Fig. 25F).

**Type material.** Holotype ♂: [MEXICO, Oax., Hwy131/5 mi. N. Telixtlahuaca/6200′ 31Aug.1982 CW. &/L. O’Brien & G. Wibmer] (ASUCOB). Paratypes (5 ♀♀): [MEXICO, Oax, 19.5/km. NW. Huitzo, Hwy./190, 2,260 m, 9.VII./1992 C.L. Bellamy] (1 ♀, ASUCOB); [MEXICO, Oax., Hwy131/same data as holotype] (1 ♀, ASUCOB; 1 ♀, MZFC; 1 ♀, CMNC); [MEXICO, Oax., Hwy131/same data as holotype] [*Isodacrys*/*minutus*/Sharp/det. C. W. O’Brien, 1999] (1 ♀, ASUCOB).

**Etymology.** Following the Náhuatl roots of the municipalities where the specimens were collected, this species is named with the Náhuatl word for weevil, okuiltontli.

**Remarks.** Adults have been collected between ~1,890 and 2,260 m above sea level.

**Comments.** All specimens except one have the scales of the elytral disc abraded at different degrees. The tegmen and some parts of the median lobe were not described in detail because they were damaged during dissection. Apparently with no sexual dimorphism.

***Isodacrys orizabae*** Sharp, 1911

(Fig. 9)

*Isodacrys orizabae* Sharp, 1911: 176. Syntypes not examined. Blackwelder, 1947: 799 (checklist); Howden, 1961: 85–86 (redescription); O’Brien & Wibmer, 1982: 46 (checklist); Morrone, 1999: 145 (checklist); Cortes-Hernández & Morrone, 2019: 50.

**Diagnosis.** Setae of elytra spatulate, from incompletely arched to erect; scrobe with anterior portion as long as posterior portion, posterior portion separated from eye by distance nearly 1.2 times width of scrobe; eyes moderate in size, slightly convex, not prominent; anterolateral margin of prothorax with one or two vestigial postocular vibrissae, when present; elytra in dorsal view variable, obovate, widest before midlength in females, subelliptical, widest at midlength in males; profemora wider than metafemora, protibiae longer than metatibiae, inner margin of protibiae with teeth; last abdominal segment tumid in females; median lobe shorter than spiculum gastrale, apex in dorsal view acute, manubrium shorter than median lobe; spermatheca u-shaped, corpus subcylindrical, slightly wide, collum inconspicuous, ramus broadly subcylindrical, short.

*Isodacrys orizabae* can be easily confused with *I. ellipticum* and *I. guatemalenum*, but it can be easily separated by setae from incompletely arched to erect, the epistome without longitudinal carina, eyes slightly convex, not prominent and last abdominal segment tumid in females. In *I. ellipticum* and *I. guatemalenum* setae are longer, from semierect to erect, the epistome with longitudinal carina joined with posterior margin of nasal plate, eyes convex, prominent, and last abdominal segment flat in both sexes.

**Material examined.** MEXICO: Oaxaca, 1.8 km S El Tule, 16.VII.1987, dry *Acacia* scrub, 1,524 m, R.S. Anderson (1, CMNC); Oaxaca, 20 km E Mitla, 3.VIII.1986, 1,800 m, H. & A. Howden (2, CMNC); Oaxaca, Hwy 175, 10 km NE Oaxaca, 16.VI.1979, 1,800 m, H. & A. Howden (1, CMNC); Oaxaca, 5 mi. SW Oaxaca, 4.VIII.1983, *Acacia* thorn scrub, 4700′, R. . Anderson, W. Maddison (9, CMNC); Oaxaca, 13 mi. SE Tlalcolula, 2.VI.1974, 6100′, C. & L. O’Brien, G. Marshall (4, ASUCOB); Puebla, 4.4 mi. SW Acatepec, 26.VII.1974, Clark, Murray, Ashe, Schaffner (5, CMNC; 6, TAMUIC); Puebla, 6 km S Acatepec, 19.VIII.1982, Clark, Cave (1, ASUCOB); Veracruz, VIII.1942, Koebele (2, CMNC); Veracruz, Hwy 150, 31 km W Orizaba, 15.VIII.1975, beating Pine, 1,880 m, B. García G. (1, ASUCOB).

**Remarks.** Adults have been collected between ~1,400 and 1,880 m above sea level, some specimens were collected on *Acacia* sp. (Fabaceae). One specimen was collected beating *Pinus* sp. (Pinaceae).

**Comments.** *Isodacrys orizabae* was described based on nine specimens from Orizaba, Veracruz, Mexico. The syntypes are at BMNH and USNM and were not examined. Identity of material examined was corroborated by comparing with specimens identified by Anne Howden and by agreement with the original species description (Sharp, 1911) and redescription (Howden, 1961).

***Isodacrys ovipennis*** (Schaeffer, 1908)

(Figs. 5F and 9)

*Pandeletejus ovipennis* Schaeffer, 1908: 215. Lectotype (Howden, 1961): ♀, not examined.

*Pandeleteius ovipennis*; Pierce, 1909: 359.

*Isodacrys ovipennis*; Pierce, 1913: 401 (new combination); Burke, 1959: 39 (actually *Isodacrys burkei*); Howden, 1961: 87–88 (redescription and lectotype designation); O’Brien & Wibmer, 1982: 46 (checklist); Howden, 1993a: 2 (checklist); Morrone, 1999: 145 (checklist); Anderson, 2002: 780; Cortes-Hernández & Morrone, 2019: 50.

**Diagnosis.** Setae lanceolate, small, from completely to incompletely arched, mostly inconspicuous; epistome with longitudinal carina joined with posterior margin of nasal plate; scrobe with anterior portion as long as posterior portion, posterior portion separated from eye by distance nearly 1.2 times width of scrobe; antennal scape and funicular antennomere I covered with scales dorsally; eyes large, slightly convex, not prominent; anterolateral margin of prothorax with three to six postocular vibrissae, mostly vestigial; elytra in dorsal view ovate, widest after midlength, intervals two and ten, and intervals five and six tumescent at their apical junctions (Fig. 5F), intervals three to five slightly elevated at base; profemora wider than metafemora, protibiae longer than metatibiae, inner margin of protibiae with teeth; median lobe shorter than spiculum gastrale, longer than temones, apex in dorsal view acute, manubrium as long as median lobe; spermatheca u-shaped; corpus subcylindrical, collum inconspicuous, ramus broadly subcylindrical, short.

*Isodacrys ovipennis* can be easily separated from *I. burkei* by anterolateral margin of the prothorax with three to six postocular vibrissae. *Isodacrys burkei* does not presents postocular vibrissae, instead a tooth extending anteriorly towards the eyes.

**Material examined.** MEXICO: Tamaulipas, Hwy101 11 mi. SW Cd. Victoria, 22.VII.1982, 4000′, C. & L. O’Brien, G. Wibmer (37, ASUCOB); Tamaulipas, 2 mi. NE San Fernando, Hwy 101, 31.VII.1970, C.W. O’Brien (1, ASUCOB); Tamaulipas, 4 mi. S San Fernando, Hwy 101, 5.V.1983, C. & L. O’Brien, G. Marshall (2, ASUCOB). UNITED STATES OF AMERICA: Texas, Cameron Co. Laguna Atascosa Nat. Wild. Ref. rd. to Laguna Madre, 20.X.1989, sweeping *Palafoxia texana var. ambigua*, R.S. Anderson, E. Riley (13, CMNC); Texas, Cameron Co. Laguna Atascosa Wdlf. Ref, 20.X.1989, E.G. Riley (5, ASUCOB); Texas, Encino, 13.III.1966, *Modiola caroliniana*, M.F. Schuster (1, CMNC); Texas, Kenedy Co. Los Olmos Creek at #77, 2 mi. S Riviera, 20.X.1989, sweeping *Palafoxia texana var. ambigua*, R.S. Anderson, E. Riley (13, CMNC; 1, CASENT); Texas, Kenedy Co. 10 mi. N Norias, 10.X.1989, D.J. Heffern (1, CMNC); Texas, Edinburg, 3.VI.1962, W.L. Sterling (1, TAMUIC); Texas, Kingsville, Kleberg Co., 25.VII.1951, H.R. Burke (1, TAMUIC); Texas, 10 miles NW La Gloria, 6.XI.1989, W.F. Chamberlain (1, TAMUIC); Texas, Kleberg Co., Jct. Los Olmos Creek & Hwy 77, 19.X.1990, T. Carlow, E. Riley (2, TAMUIC); Texas, Palmitto Hill, 10 mi. W Boca Chica, 13.X.1985, R. Turnbow (1, CMNC).

**Remarks.** Adults have been collected on *Palafoxia texana var. ambigua* (Asteraceae) and *Modiola caroliniana* (Malvaceae). A big series from Tamaulipas was collected by the O’Briens at ~1,200 m above sea level.

**Comments.** *Isodacrys ovipennis* was described based on 11 specimens (Howden, 1961) from Brownsville, Texas, United States of America. The lectotype is in USNM and was not examined. Paralectotypes are also at USNM. Although Howden (1961) stated that the type series was deposited in USNM, there is an additional syntype from databased material in Brigham Young University Arthropod Museum (BYUC; Gries, Gilbert & Franz, 2014, available at https://scan-bugs.org/portal/collections/individual/index.php?occid=37487711).

***Isodacrys schwarzi* Champion, 1911**

(Figs. 3D and 9)

*Isodacrys schwarzi* Champion, 1911: 341. Syntypes not examined. Blackwelder, 1947: 799 (checklist); Howden, 1961: 81–82 (redescription); O’Brien & Wibmer, 1982: 46 (checklist); Morrone, 1999: 145 (checklist); Cortes-Hernández & Morrone, 2019: 50.

**Diagnosis.** Antennal scape covered with scales at apex dorsally (Fig. 3D); anterolateral margin of prothorax with tuft of postocular vibrissae; basal margin of elytra angularly emarginate, with sutural interval at summit of declivity strongly tumid, apical declivity strongly concave (Fig. 3D); and inner margin of protibiae without teeth.

*Isodacrys schwarzi* can be easily separated by its well-developed postocular vibrissae, which in *I. buchanani* are reduced to one or two small postocular vibrissae.

**Comments.** *Isodacrys schwarzi* was described based on two specimens from Monterrey, Nuevo León, Mexico. One female syntype is deposited at USNM and the other probably at BMNH. None of them were examined. No additional material was examined. Information regarding the deposition and sex of the remaining syntype is unknown.

Diagnosis is based on habitus photographs and on redescription by Howden (1961). See Champion (1911) and Howden (1961) to see the full description.

***Isodillex* Cortés-Hernández, new genus**

(Figs. 1, 26–27)

**Figure 26:**
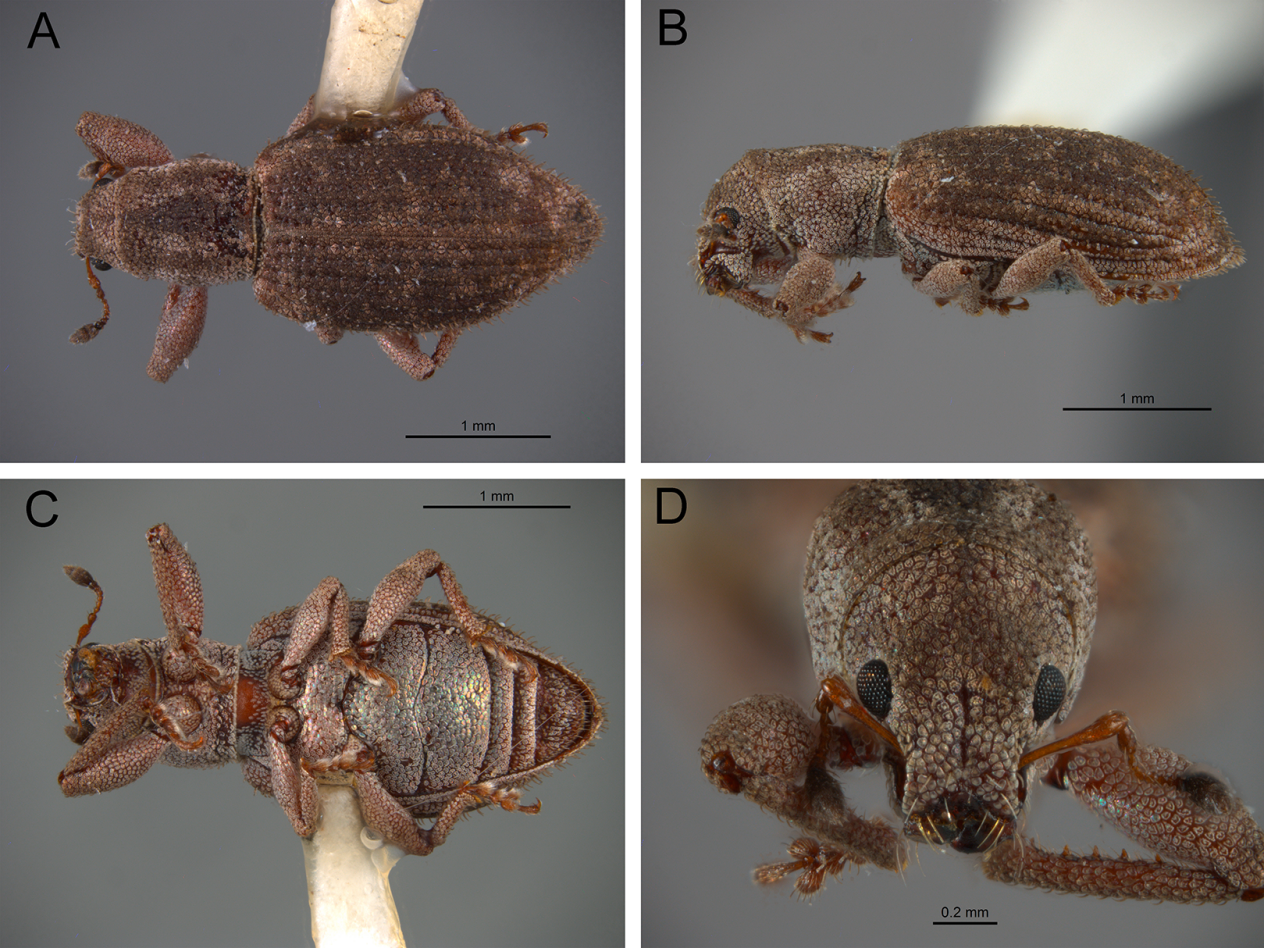
*Isodillex plumosum* new genus and new species. (A) Dorsal habitus; (B) lateral habitus; (C) ventral habitus; (D) head in anterior view.

**Figure 27:**
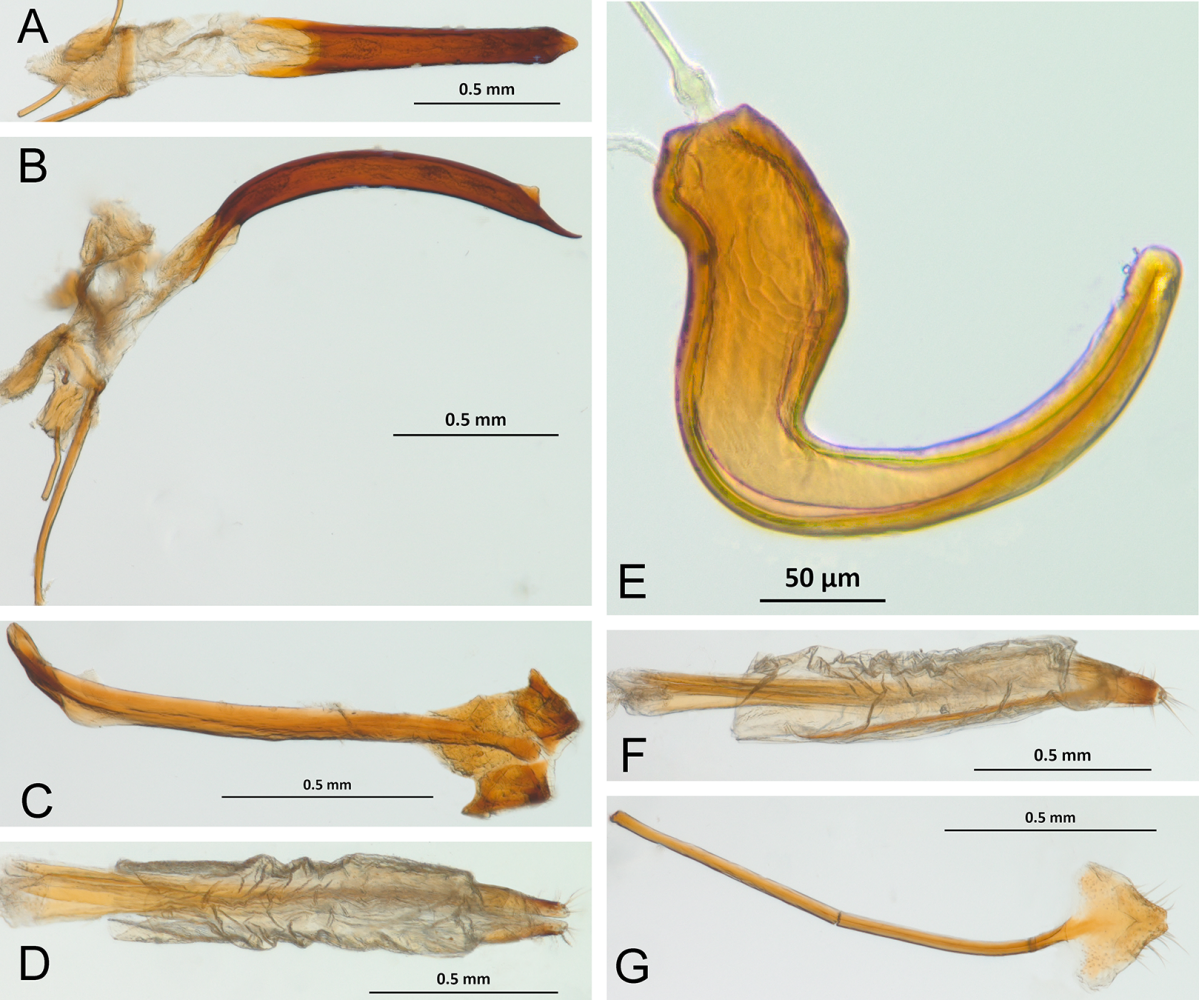
*Isodillex plumosum* new genus and new species, genitalia. (A) Median lobe in dorsal view; (B) median lobe in lateral view; (C) male hemisternites VIII and sternite IX in dorsal view; (D) ovipositor in dorsal view; (E) spermatheca; (F) ovipositor in lateral view; (G) female sternite VIII in dorsal view.

Type species: *Isodillex plumosum*

urn:lsid:zoobank.org:act:2AD213BD-E6DE-4146-A578-426D26C2002E

**Diagnosis.** Dorsal scales contiguous, not overlapping, polygonal; dorsolateral margins of rostrum subparallel, nasal plate with anterior margin medially indented, posterior margin of nasal plate carinate, epistome in continuous plane with remainder of rostrum, scales of different shape and shinier than remainder of rostrum, posterior margin of epistome indistinct; scrobe obtusely angled, reaching ventral surface of rostrum; anterior portion of scrobe about as long as posterior portion; posterior portion of scrobe separated from eye by distance shorter than width of scrobe; head deflexed, directed ventrally; mandibles covered with setae, without scales, prementum incompletely concealing maxillae; antennal scape capitate, abruptly thickened at apical region, funicular antennomere I longer than funicular antennomere II; pronotum in dorsal view subcylindrical, nearly as wide as long; anterolateral margin of prothorax without postocular vibrissae; intercoxal process of prosternum continuous, separating procoxae; humeri absent, elytra in dorsal view with seven intervals visible at base, basal margin of elytra roundly emarginate; prolegs larger than meso- and metalegs, tarsal claws free; anterior margin of ventrites III, IV and V flat, not sulcate; apex of lamina of female sternite VIII blunt; manubrium shorter than median lobe.

*Isodillex* can be separated from *Isodacrys* by combination of the following characters: dorsal scales contiguous, not overlapping, anterolateral margin of the prothorax without postocular vibrissae, prolegs larger than metalegs, anterior margin of ventrites III, IV and V flat, not sulcate and male genitalia with manubrium shorter than median lobe. In *Isodacrys* the dorsal scales are overlapping non-linearly (except *I. obrienorum*), anterolateral margin of the prothorax with postocular vibrissae or not, the prolegs from slightly larger than the metalegs to equal in size, anterior margin of ventrites III, IV and V sulcate and male genitalia with manubrium as long as median lobe or longer (except *I. orizabae*).

**Description - Habitus.** Males 2.8–3.0 mm long, 1.0–1.2 mm wide; females 2.4–3.2 mm long, 1.0–1.4 mm wide. Scales polygonal, contiguous, not overlapping. **Rostrum.** Dorsolateral margins of rostrum sub-parallel; dorsal surface flat. Epistome in continuous plane with remainder of rostrum. Nasal plate with anterior margin mesally indented; posterior margin carinate. Scrobe deep, bent, reaching ventral surface of rostrum; anterior portion of scrobe about as long as posterior portion; posterior portion of scrobe separated from eye by distance shorter than width of scrobe. Funicular antennomere I clavate, large, wider and longer than remaining funicular antennomeres. **Head.** In lateral view with dorsal outline smoothly continuing with rostrum. **Prothorax.** Pronotum in dorsal view subcylindrical, nearly as wide as long, lateral outlines sinuate; base of pronotum slightly emarginate medially. Anterolateral margin of prothorax without postocular vibrissae. **Elytra.** 2.2–2.6 times longer than pronotum. Basal margin of elytra emarginate. Humeri absent. Elytra in dorsal view subelliptical, widest at midlength, seven intervals visible at base. **Legs.** Procoxae narrowly separated; prolegs larger than meso- and metalegs; profemora fusiform, subequal in length to metafemora, one fifth wider than metafemora; protibiae straight, one fifth longer than metatibiae, inner margin with teeth. Tarsal claws free. **Abdomen.** Anterior margins of ventrites III, IV and V flat, not sulcate. **Genitalia**. ♂: Hemisternites VIII teardrop-shaped, outer corner not truncate; basal plate of sternite IX lightly sclerotized, distally bifurcate, tips of bifurcations sclerotized; spiculum gastrale as long as median lobe, distally continuing through basal plate; ring of tegmen without parameres; manubrium shorter than median lobe. ♀: Lamina of sternite VIII triangular, apex blunt. Ovipositor with distal gonocoxites slightly sclerotized laterally, styli represented by single thickened seta; two ventral baculi close to each other, separated by distance similar to width of one baculus; vagina with two pairs of lightly sclerotized proximal rods.

**Etymology.** From the combination of *Isodacrys* and the latin adjective *ilex*, which means false/fraudulent. *Isodillex* therefore means “false *Isodacrys*” based on their external resemblance. Gender neuter.

New combination

***Isodillex minutum*** (Sharp, 1911), **new combination**

(Fig. 2)

*Isodacrys minutus* Sharp, 1911: 176–177. Holotype or lectotype not designated; syntypes not examined.

*Isodacrys minuta*; Blackwelder, 1947: 799 (checklist).

*Isodacrys minutum*; Howden, 1961: 84 (redescription); O’Brien & Wibmer, 1982: 46 (checklist); Morrone, 1999: 145 (checklist); Cortes-Hernández & Morrone, 2019: 50.

**Diagnosis.** Setae of elytra lanceolate, completely arched at base, becoming erect towards declivity; frons convex, rounded; anterolateral margin of prothorax without modified scales, without postocular vibrissae; elytra in lateral view with dorsal outline evenly arcuate from base to apex; interval six slightly depressed at base; spermatheca Y-shaped, collum subcylindrical, short, ramus broadly subcylindrical, longer than collum, cornu curved near corpus, wide, short, not reaching apex of ramus.

*Isodillex minutum* can be readily separated from *I. plumosum* by its convex frons, anterolateral margin of the prothorax without modified scales and the elytra in lateral view with dorsal outline evenly arcuate from base towards apex. In *Isodillex plumosum* the frons is not prominent, anterolateral margin of the prothorax with plumose scales and elytra with apical declivity oblique.

**Material examined.** “MEXICO: MICHOACÁN, 2 mi. S Carapan, 6.VII.1985, Woolley, Zolnerowich” (5 specimens, TAMUIC; 3 specimens, CMNC), “MEXICO: MICHOACÁN, 18.5 km E Morelia, 28.VII.1988, Pine/oak/Acacia, 2,220 m, R. S. Anderson” (1 specimen, CMNC), “MEXICO: MICHOACÁN, 17 mi. W Pátzcuaro, 14.VIII.1982, 7700′, C. & L. O’Brien, G. Wibmer” (1 specimen, ASUCOB).

**Remarks.** Adults have been collected between ~850 and 2,440 m above sea level, some of them sweeping lupine plants (Fabaceae; see Howden, 1961). Males unknown.

**Comments.** *Isodillex minutum* was described based on four specimens from Omilteme, Guerrero, Mexico. One syntype is deposited at BMNH and deposition of remaining syntypes remains unknown. Syntypes not examined. Identity of the material examined was based on specimens identified by Anne Howden and by agreement with the original species description.

***Isodillex plumosum* Cortés-Hernández, new species**

(Figs. 1, 26–27)

urn:lsid:zoobank.org:act:955038E3-B522-4A22-9149-896ED0092C6B

**Diagnosis.** Setae of elytra spatulate, completely arched at base, becoming erect towards apical declivity (Fig. 26B); frons not prominent (Fig. 26B); anterolateral margin of prothorax with plumose scales; elytra with apical declivity oblique, intervals with no elevations or depressions; median lobe with basal half more curved in lateral view, apical opening subelliptical, apex in dorsal view rounded; spermatheca u-shaped, corpus cylindrical, collum and ramus inconspicuous.

*Isodillex plumosum* is easily separated from *I. minutum* by anterolateral margin of the prothorax with plumose scales, whereas in *I. minutum* the plumose scales are absent.

**Description - Habitus.** ♂ 2.8–3.0 mm long, 1.0–1.2 mm wide; ♀ 2.7–3.2 mm long, 1.1–1.4 mm wide. Scales polygonal, with raised margins, granulate, contiguous, not overlapping, from brown to pale brown in coloration (Fig. 26A). Head at vertex inconspicuous longitudinal brown vitta, frons mainly with brown scales; genae and rostrum covered pale brown scales. Disc of pronotum with three longitudinal vittae of brown scales; sides of prothorax with pale brown scales. Disc of elytra and epipleura mostly brown scales, with irregular patches of pale brown scales; intervals nine and ten mostly covered with pale brown scales. Setae lanceolate on head and pronotum, most completely arched to incompletely arched on head, inconspicuous on temple; completely arched in pronotum. Setae of elytra spatulate, completely arched at base, becoming erect at apical declivity (Fig. 26B). **Rostrum.** Dorsolateral margins of rostrum sub-parallel; dorsal surface flat; median sulcus conspicuous, posterior end marked by fovea between eyes (Fig. 26D). Epistome in continuous plane with remainder of rostrum, covered by three rows of shiny, small scales (Fig. 26D); anterior margin bearing five epistomal setae on each side; posterior margin limiting with median sulcus. Nasal plate with surface shiny, rugulose; anterior margin mesally indented; posterior margin V-shaped, carinate. Scrobe deep, bent, reaching ventral surface of rostrum; dorsal margin obtusely angled; anterior portion of scrobe about as long as posterior portion; posterior portion of scrobe separated from eye by distance shorter than width of scrobe. Antenna setose, without scales (Fig. 26A); scape capitate; funicular antennomere I clavate, wider and longer than remaining funicular antennomeres; funicular antennomere II clavate, longer than remaining funicular antennomeres; funicular antennomeres III to VII moniliform, distal antennomeres becoming broader. **Head.** In lateral view with dorsal outline smoothly continuing with rostrum, frons not prominent (Fig. 26B); eyes moderate in size, sub-oval, prominent, lateral, separated from outline of frons by distance shorter than half diameter of eye. **Prothorax.** Pronotum in dorsal view subcylindrical, nearly as wide as long, lateral outlines sinuate; in lateral view dorsal outline sinuate, anterior and posterior constrictions conspicuous; base of pronotum slightly emarginate medially (Fig. 26A). Anterolateral margin of prothorax without postocular vibrissae, with set of plumose scales projected anteriorly. **Elytra.** 2.2–2.4 times longer than pronotum, at base 1.1–1.2 times wider than base of prothorax. Basal margin of elytra emarginate. Elytra in dorsal view subelliptical, widest at midlength, seven intervals visible at base (Fig. 26A). Elytra in lateral view with dorsal outline nearly straight, becoming slightly convex towards summit of apical declivity; apical declivity oblique (Fig. 26B). Elytral intervals with no elevations or depressions. **Legs.** Procoxae narrowly separated (Fig. 26C), intercoxal process 1/6–1/4 width of procoxa; profemora fusiform, subequal in length to metafemora, 1.2 times wider than metafemora; protibiae straight, 1.2 times longer than metatibiae, inner margin with four to six teeth. Dorsal surface of tarsi setose, first and second tarsomeres with appressed scales or not (probably lost by abrasion). Tarsal claws free. **Abdomen.** Anterior margin of ventrites III, IV and V flat, not sulcate (Fig. 26C). **Genitalia.** ♂: Hemisternites VIII teardrop-shaped, outer corner not truncate (Fig. 27C); basal plate of sternite IX lightly sclerotized, distally bifurcate, tips of bifurcations sclerotized; spiculum gastrale as long as median lobe, distally continuing through basal plate; ring of tegmen without parameres; manubrium 0.7 times length of median lobe; median lobe 9.5 times longer than wide in dorsal view, basal half more curved in lateral view, apex in lateral view slightly bent (Fig. 27B), apical opening subelliptical, apex rounded. ♀: Lamina of sternite VIII triangular, apex blunt, bifurcate (Fig. 27G); spiculum ventrale 4.4–4.8 times length of lamina. Ovipositor with distal gonocoxites slightly sclerotized laterally, styli represented by single thickened seta; two ventral baculi close to each other, separated by distance similar to width of one baculus; vagina with two pairs of lightly sclerotized proximal rods (Fig. 27F). Spermatheca u-shaped, corpus cylindrical; collum and ramus inconspicuous; cornu strongly curved near corpus, distal 2/3 bent, elongate, reaching apex of ramus, apex abruptly narrowed (Fig. 27E). **Sexual dimorphism.** Ventrites I and II slightly flattened mesally in males, convex in females; apex of ventrite V emarginate in males, rounded in females.

**Type material.** Holotype (♂): [61 mi. W. Fresnillo/Zacatecas, MEX/8000 ft. VI-25-54/R. H. Brewer] [III#3] [Isodacrys/n.sp. #2/AH ‘62] [CASENT/8424336]. Paratypes (3 ♂♂ and 11 ♀♀): [61 mi. W. Fresnillo/same data as holotype] [III#3] [Cal Acad.] [CASENT/8424322] (1 ♂ CASENT); [61 mi. W. Fresnillo/same data as holotype] [III#3] [CASENT/8424323] (1 ♂ CASENT); [61 mi. W. Fresnillo/same data as holotype] [III#3] [CASENT/8424331] (1 ♂ CASENT); [61 mi. W. Fresnillo/same data as holotype] [III#3] [Cal Acad.] [Isodacrys/n.sp.#2] [CASENT/8424321] (1 ♀ CASENT); [61 mi. W. Fresnillo/same data as holotype] [III#3] [CASENT/8424324] (1 ♀ CASENT); [61 mi. W. Fresnillo/same data as holotype] [III#3] [CASENT/8424325] (1 ♀ CASENT); [61 mi. W. Fresnillo/same data as holotype] [III#3] [CASENT/8424326] (1 ♀ CASENT); [61 mi. W. Fresnillo/same data as holotype] [III#3] [CASENT/8424327] (1 ♀ CASENT); [61 mi. W. Fresnillo/same data as holotype] [III#3] [CASENT/8424328] (1 ♀ CASENT); [61 mi. W. Fresnillo/same data as holotype] [III#3] [CASENT/8424329] (1 ♀ CASENT); [61 mi. W. Fresnillo/same data as holotype] [III#3] [CASENT/8424330] (1 ♀ CASENT); [61 mi. W. Fresnillo/same data as holotype] [III#3] [CASENT/8424332] (1 ♀ CASENT); [61 mi. W. Fresnillo/same data as holotype] [III#3] [CASENT/8424333] (1 ♀ CASENT); [61 mi. W. Fresnillo/same data as holotype] [III#3] [CASENT/8424334] (1 ♀ CASENT); [61 mi. W. Fresnillo/same data as holotype] [III#3] [CASENT/8424335] (1 ♀ CASENT).

**Etymology.** From the latin word *plumosum*, referring to the plumose setae present at the anterolateral margin of prothorax.

**Comments.** Length of temones relative to median lobe is not described because temones were incomplete on the specimens dissected.

**Key to identify *Isodacrys* species**

1 Elytra with sutural interval at summit of declivity strongly tumid, apical declivity strongly concave (Fig. 3D); inner margin of protibiae without teeth; antennal scape covered with scales only at apex dorsally (Fig. 3D)2
1′ Elytra with sutural interval at summit of declivity not or only slightly tumid, apical declivity straight (Fig. 6A); inner margin of protibiae variable; antennal scape with scales or not3
2 Anterolateral margin of prothorax with one or two postocular vibrissaeI. buchanani
2′ Anterolateral margin of prothorax with tuft of postocular vibrissae (Fig. 5C)I. schwarzi
3 Elytra with intervals seven to nine strongly concave at base (Fig. 10B); lamina of female sternite VIII oval (Fig. 11C)*I. antrum* new species
3′ Elytra with intervals seven to nine at most slightly depressed; lamina of female sternite VIII triangular (Fig. 13C)4
4 Elytra in dorsal view with seven visible elytral intervals at base (Fig. 5F); inner margin of protibiae with teeth or small denticles; anterolateral margin of prothorax with postocular vibrissae usually present (Fig. 3B)5
4′ Elytra in dorsal view with five visible elytral intervals at base (Fig. 5E); inner margin of protibiae without teeth; prothorax with postocular vibrissae usually absent13
5 Antennal scape covered with scales at dorsal apex (Fig. 3D); elytra in dorsal view ovate, widest after midlength (Fig. 5F), apical junction of intervals two and ten, five and six swollen (Fig. 5F)6
5′ Antennal scape without scales (Fig. 5A); elytra in dorsal view elliptical (Fig. 5D) or obovate, never widest after midlength, apical junction of intervals two and ten, and intervals five and six not swollen7
6 Anterolateral margin of prothorax with conspicuous tooth, without postocular vibrissae; anterior portion of scrobe shorter than posterior portionI. burkei
6′ Anterolateral margin of prothorax without tooth, with three to six postocular vibrissae; anterior portion of scrobe as long as posterior portion (Fig. 4E)I. ovipennis
7 Prolegs larger than metalegs (Figs. 5D, F), promefoma wider than metafemora, protibiae longer than metatibiae8
7′ Prolegs subequal in size to metalegs (Fig. 6A)11
8 Anterolateral margins of prothorax without postocular vibrissae (Fig. 10B); posterior margin of scrobe separate from eye by distance equal to width of scrobe; setae of elytra long, from semierect to erect (Fig. 5D)9
8′ Anterolateral margins of prothorax with postocular vibrissae (Fig. 3B); posterior margin of scrobe separate from eye by distance at least one fifth longer than width of scrobe (Fig. 4E); setae of elytra moderate in length, curvature of setae various10
9 Setae of elytra very long, subparallel, erect (Fig. 5D); spermatheca with collum and ramus inconspicuous (Fig. 27E)I. guatemalenum
9′ Setae of elytra spatulate, from semierect to erect; spermatheca with collum inconspicuous, ramus very short, broadly cylindricalI. ellipticum
10 Setae of elytra spatulate, from incompletely arched to erect; eyes moderate in size, slightly convex, not prominent; prothorax in dorsal view with subtle constrictions, lateral margins slightly sinuateI. orizabae
10′ Setae of elytra lanceolate, small, completely arched; eyes large, subcircular, conspicuously convex (Fig. 4B); prothorax in dorsal view with lateral margins strongly sinuate between anterior and posterior constrictionsI. mexicanum
11 Setae of elytra lanceolate, completely arched; scrobe right-angled (Fig. 6A), anterior portion shorter than posterior portion; sulci of ventrites III to V with posterior margin sharp, conspicuously delimiting sulciI. crispum
11′ Setae of elytra spatulate, from incompletely arched to erect; scrobe obtusely angled (Fig. 4E), anterior portion as long as posterior portion; sulci of ventrites III to V with posterior margin effaced, ventrites becoming flat posteriorly (Fig. 6F)12
12 Anterolateral margin of prothorax with more than six postocular vibrissae, some well-developed (Fig. 4E); apical margins of elytra bisinuate (Fig. 6B); posterior portion of scrobe separated from eye by distance 1.2 times width of scrobe (Fig. 4E)I. apicale
12′ Anterolateral margin of prothorax without postocular vibrissae (Fig. 10B); apical margins of elytra straight, conjointly rounded; posterior portion of scrobe separated from eye by distance shorter than width of scrobe (Fig. 24B)*I. okuiltontli* new species
13 Scales of dorsal surface of body mostly contiguous and not overlapping (Fig. 22A); setae mostly erect (Fig. 22B); epistome with scattered scales, integument exposed (Fig. 22D)*I. obrienorum* new species
13′ Scales of dorsal surface of body variously overlapping non-linearly (Fig. 5E); setae usually not erect, at most erect at apical declivity of elytra; epistome with scattered scales but integument not substantially exposed (Fig. 24D)14
14 Rostrum with longitudinal sulci mesad of dorsolateral margins at bent of scrobes (Fig. 4D); scrobe with anterior portion as long as posterior portion; profemora as wide as metafemora15
14′ Rostrum without longitudinal sulci mesad of dorsolateral margins at bent of scrobes; scrobe with anterior portion shorter than posterior portion (Fig. 12B); profemora slightly wider than metafemora17
15 Scrobe right-angled (Fig. 6A); posterior portion separated from eye by distance shorter than width of scrobe; elytra obovate, widest before midlength, without depressionsI. geminatum
15′ Scrobe obtusely angled (Fig. 4E); posterior portion separated from eye at least by distance equal to width of scrobe; elytra subelliptical, widest at midlength (Fig. 5D), intervals seven to nine slightly depressed at base16
16 Frons not prominent (Fig. 5C); posterior portion of scrobe separated from eye by distance equal to width of scrobe; elytral setae spatulate, semierect, becoming erect at apex of elytraI. brevirostre
16′ Frons very prominent (Fig. 18B); posterior portion of scrobe separated from eye by distance at least twice width of scrobe (Fig. 18B); elytral setae lanceolate, completely arched to incompletely arched*I. frontalis* new species
17 Antennal scape covered with scales at apex dorsally (Fig. 3D); all tarsomeres with appressed scales (Fig. 14B); pronotum covered with white scales medially; spermatheca with collum produced, bent basally towards ramus, contiguous with corpus (Fig. 15F)*I. confusum* new species
17′ Antennal scape without scales (Fig. 5A), if present reduced to 1 or 2 scales; all tarsomeres with appressed scales or not; scale pattern of pronotum various; spermatheca with collum produced but not contiguous with corpus (Fig. 17E)18
18 Pronotum covered with white scales medially (Fig. 16A); spermatheca comma-shaped, collum produced, subcylindrical, bent basally towards ramus, not contiguous with corpus*I. fasciatum* new species
18′ Pronotum covered with brown scales medially (Fig. 12A); shape of spermatheca various, collum not bent basally towards ramus19
19 Posterior portion of scrobe separated from eye by distance equal to width of scrobe (Fig. 20B); spermatheca comma-shaped, collum produced, conical*I. kuchii* new species
19′ Posterior portion of scrobe separated from eye by distance 1.5–1.6 times width of scrobe (Fig. 12B); spermatheca peanut-shaped (Fig. 13D)*I. carlae* new species

## Discussion

### Generic relationships amongst *Isodacrys*-related Tanymecini

The present analysis has several implications on the relationships among the New World Tanymecini. The monophyly of Tanymecini has not been tested under a comprehensive approach. Nonetheless, current studies suggest that they may be polyphyletic (Gillet et al., 2018), with historical mistakes in classification and interpretation related to the presence of postocular vibrissae at the anterolateral margin of the prothorax in the tribe that consequently ended up in taxonomic amendments excluding several lineages from the tribe (Morrone, 1998; Franz, 2012; Marvaldi et al., 2018; Del Río & Lanteri, 2019b). Nine of the 16 genera of Tanymecini distributed in the New World were included in the analysis, but further discussion of the natural history of the tribe is out of the scope of this work. Further analyses should be carried out for tanymecine lineages that remain poorly understood (Cortes-Hernández & Morrone, 2019). As previously stated, traditional characters for grouping New World tanymecines are highly homoplastic and therefore should be taken with caution. This does not mean that they are not phylogenetically informative, instead they likely represent recent transformations that can contribute to the understanding of smaller clades within Tanymecini.

The Tanymecini clade is well supported by eight synapomorphies (epistome distinct 7:1, scrobe in lateral view strongly curved, directed to ventral surface of rostrum 15:1, rostrum in lateral view directed ventrally 19:1, antennal scape capitate, abruptly thickened at apical region 26:1, funicular antennomere I longer than funicular antennomere II 27:2, pronotum in dorsal subglobose 28:1, pronotum in dorsal view with constriction of margins 30:1 and prothorax in lateral view oblique, dorsally produced anteriorly 31:1) and one homoplastic character state (presence of postocular vibrissae 32:0). The basal split between *Minyomerus* and the remaining Tanymecini can be related with the psammophilic condition of the genus (i.e., adapted to live in sandy environments; Jansen & Franz, 2015).

Neither *Pandeleteinus* nor *Pandeleteius* were recovered as monophyletic. *Pandeleteinus* presented a high degree of overlap of several character states with both West Indian and continental lineages. In the case of *Pandeleteius*, which contains approximately 161 described species, the present taxon sampling is far from representing a comprehensive extent to go deep on insights about its naturalness.

The clade aff. *Pandeleteinus* sp.-*Isodrusus guajavus* is the sister group of the *Paradacrys ensiformis*- *I. antrum* clade, supported by one apomorphy (anterior margin of nasal plate slightly indented 5:1) and two homoplastic character states (dorsal scales contiguous, not overlapping 1:0 and manubrium of tegmen shorter than median lobe 72:0, both with different character states for *Isodacrys* clade). The clade aff. *Pandeleteinus* sp.-*Isodrusus guajavus* is supported by two homoplastic character states regarding the shape of the scrobe and its distance to eye (chars. 15 and 18). It has a basal division splitting aff. *Pandeleteinus* sp. from the clade aff. *Isodrusus* sp.-*Isodrusus guajavus*. The undescribed species from Colombia fits under the current *Isodrusus* circumscription (Cortés-Hernández & Anderson, 2019), here supported by three character states (tarsal claws connate 60:1, sulcus in ventrites III, IV and V along its entire width 62:1 and posterior margin of sulcus in ventrites III, IV and V present, sharply delimiting sulcus 63:0); although homoplastic, they could represent true synapomorphies under a different scope.

The West Indian *Paradacrys ensiformis*-*Scalaventer subtropicus* clade is the sister group to the *Isodillex*-*Isodacrys* clade based on one synapomorphy (seven elytral intervals visible in dorsal view 40:0, although state 1, which is five elytral intervals visible in dorsal view, is present in *Isodacrys kuchii*-*I. obrienorum* clade) and one homoplastic character state (postocular vibrissae at anterolateral margin of prothorax absent 32:0). Within the *Paradacrys ensiformis*-*S. subtropicus* clade, supported by two homoplastic characters (presence of longitudinal carina on the epistome 10:1 and posterior margin of scrobe ending before ventral surface of rostrum 16:0, with reversal in the clade *S. cyrillae*-*S. subtropicus*), each genus is recovered as monophyletic in the following sequence: *Paradacrys* (*Paululusus* (*Scalaventer*)).

There are four homoplastic character states supporting the relationship between *Isodacrys* and *Isodillex* (anterior portion of scrobe as long as posterior portion 17:1, elytra in dorsal view subelliptical 38:3, elytral humeri absent 39:1 and basal margin of elytra roundly emarginate 42:1). Some of these homoplastic character states (chars. 38, 39 and 42), all related to the condition of the elytra, as noted by Lanteri & Del Río (2017), likely evolve convergently in relation with the loss of metathoracic wings and if not accompanied by other synapomorphies should not be considered evidence of a close phylogenetic relationship. Although apterism can be homoplastic in certain lineages like island-inhabiting entimines (Howden, 1970; Franz, 2012; *Paululusus* as here presented), in continental clades like *Isodacrys* and *Minyomerus* (see Jansen & Franz, 2015: 117) it remained constant. This can be related to both historical and ecological constraints. Based on the information in hand on adult morphology, a close relationship between *Isodacrys* and *Isodillex* clade is proposed but additional sources of information should be added to strengthen this hypothesis.

### Delimitation of *Isodacrys* and phylogenetic insights

The present analysis represents a first approximation of the relationships within *Isodacrys* and is suited to address the research questions regarding the monophyly of *Isodacrys* and its relationships with other Tanymecini. None of the character states used to diagnose *Isodacrys* (Howden, 1961, p. 76) resulted as a synapomorphy when analyzed in a phylogenetic context.

Although the length of the tegmen relative to the median lobe (char. 72:1,2) represents an apomorphy for *Isodacrys* in the trees obtained with apparently two unique character states shared among its species, there is a reversal in *I. orizabae*.

Although there is apparently a high degree of incongruence, translated in many clades supported by one or several homoplastic characters, they still represent relevant grouping information for *Isodacrys* species. As one of the main research questions was to elucidate the generic position of the genus within the American Tanymecini (as discussed in the previous section), an extensive taxon sampling of putatively related genera was conducted. This inclusive approach implied the possibility of putative convergent evolution of non-immediate sister lineages, as supported by our results. A more exclusive approach could transform some of the homoplastic character states into synapomorphies but would also weaken/modify the displayed phylogenetic relationships and the question would not be even tested.

Howden (1961, 1969, 1970) highlighted the relevance of genitalia within Tanymecini lineages. According to our results and preliminary data matrices including some of the traits discussed by Howden (e.g., length/width ratio of aedeagus, curvature of aedeagus in lateral view), these characters did not show clear grouping information in the taxa included and exhibited high degree of overlap among New World Tanymecini. Some characters related to the spermatheca have been considered in previous works (Lanteri & Del Río, 2008; Girón & Franz, 2012; Franz, 2012; Jansen & Franz, 2015; Lanteri & Del Río, 2017). Efforts to include characters related to this structure were conducted in preliminary data matrices but the structures resulted quite variable interspecifically within several genera of Tanymecini, including *Isodacrys*, and also convergently similar in species of non-sister genera. If parthenogenesis takes place in *Isodacrys*, it could be a possible explanation for the strong variation of the spermatheca. Dybas (1966) stated that a spermatheca is a non-functional structure in completely parthenogenetic species. Such structures may become vestigial in time, or, at least, more variable because of less stringent selection. Additional to this, depending on how the specimens are prepared for examination (temperature, type of liquid), the spermathecal shape can visibly change during preparation. Based on this and on the high degree of incongruence depicted by the spermathecal characters, we decided to exclude them from the final analysis. Future analyses should consider intraspecific variation when studying reproductive characters in entimine weevils where parthenogenesis has been corroborated or suspicious. Further approaches remain to be explored (e.g., morphometry) in order to evaluate the phylogenetic significance of these characters for parthenogenetic species.

From the 20 species here included in *Isodacrys* males remain unknown for eight of them: *Isodacrys antrum*, *I. brevirostre*, *I. carlae*, *I. crispum*, *I. ellipticum*, *I. fasciatum*, *I. frontalis* and *I. geminatum* (see also comments on *Isodacrys schwarzi*). Based on the lack of males throughout different localities where specimens have been collected (see material examined for further information) we hypothesize that these species might be parthenogenetic (Howden, 1961). According to Lanteri & Normark (1995), this mode of reproduction is the most plausible explanation when males are absent in different populations of the same species in naupactine weevils. Further collecting will be needed as well as cytological and genetic studies to corroborate parthenogenesis in *Isodacrys* species and other genera of Tanymecini where parthenogenesis is suspicious (Jansen & Franz, 2015; Cortés-Hernández & Anderson, 2019). Additionally, parthenogenesis can be supported by its correlation with flightlessness given the potential difficulties of flightless insects to locate mates (Roff, 1990; Lanteri & Normark, 1995). Under this scenario, no energy is invested in mating and wing-development. The loss of metathoracic wings has been also correlated with greater fecundity and environmental stability where dispersal does not impact in long-term survival of populations (Roff, 1990; Wagner & Liebherr, 1992), as may be the case for most Isodacrys species (parthenogenetic or not), which are found in mountains and tropical montane forests, considered stable habitats (Roff, 1990; Wagner & Liebherr, 1992). Parthenogenesis can increase fecundity too, because all the eggs can produce females and are not “wasted” in producing males (Dybas, 1966). In stable environments, low genetic variability of parthenogenetic species may result in greater success of progeny (Jansen & Franz, 2015).

If parthenogenesis exists in *Isodacrys*, and assuming that all species where males remain unknown are parthenogenetic, accordingly to the MPT’s (Fig. 2) parthenogenesis evolved at least five times within *Isodacrys*: *I. ellipticum*, *I. crispum* clade, *I. apicale*, *I. kuchii* clade and *I. brevirostre* clade. Parallel evolution of parthenogenesis has been also discussed for naupactine weevils (Lanteri & Normark, 1995; Normark, 1996) and for *Minyomer*us *(Jansen & Franz, 2015). T*his can be reflected on the interspecific variation of the genitalia (e.g., shape of spermatheca, length/width of median lobe) among closely related *Isodacrys* species and the consistent information exhibited by the genitalia-related characters in major clades within the genus (Fig. 2, see optimizations in characters 65, 70, 71 and 72 for *Isodacrys*). Although genitalia characters are traditionally associated as species-grouping characters (Vaurie, 1976; Del Río & Lanteri, 2019a), it is not necessarily the pattern when alternative modes of reproduction take place. Other sources of information should be explored to better support the interspecific relationships among *Isodacrys* species.

According to the most-parsimonious trees, a remarkable tendency to certain traits within *Isodacrys* can be noticed: *Isodacrys kuchii*-*I. antrum* clade (Fig. 2) is represented by species with only five elytral intervals (char. 40:1); within this clade there is a tendency to lose the postocular vibrissae at anterolateral margin of the prothorax and loss of teeth along inner margin of the protibiae (chars. 32:0 and 54:0), reduction in size of the prolegs relative to the metalegs (chars. 52:0, although with reversal to state 1 in *I. kuchii* clade, and 53:0) and the manubrium longer than the median lobe (char. 72:2). These trends to reduction and loss seem to be an important division among *Isodacrys* species. Developmental studies should be carried out in order to obtain further information regarding these characters and their putative implications for the natural history of lineages within *Isodacrys*.

We believe in the potential existence of more species of *Isodacrys* unknown to science throughout Mexico and Central America. The compiled information from museum specimens indicates that adults of five species were found in leaflitter, a niche that has largely been unexplored for Tanymecini.

### Taxonomic amendments

We propose the following nomenclatural changes: (i) The genus *Isodillex* is here proposed for grouping *Isodillex minutum* (Sharp) new combination and *Isodillex plumosum* new species and (ii) *Isodacrys* is re-defined as a genus containing 20 species, eight here described as new.

## Conclusions

The phylogenetic analysis based on adult morphology recovers the genus *Isodacrys* as a monophyletic entity composed by 20 described species, eight of them herein described. Additionally, *Isodillex* new genus, which is sister to *Isodacrys*, is erected to accommodate one new species and species previously considered as *Isodacrys*. Finally, the West-Indian *Paradacrys*-*Scalaventer* clade is sister to the *Isodillex*-*Isodacrys* clade. Future directions to enhance our understanding of the natural history of these weevil lineages can be field observations, breeding experiments of potentially parthenogenetic species and biogeographic analyses regarding its relationships with West Indian tanymecines. Additional sources of information with potential phylogenetic signal (e.g., molecular data) will be necessary to strengthen/test the current species-level relationships inferred within *Isodacrys* as well as its relationships with other Tanymecini lineages.

## Supplemental Information

10.7717/peerj.10191/supp-1 Supplemental Information 1 Nexus file.

## Additional Information and Declarations

### Data Availability

The following information was supplied regarding data availability: Specimens examined Acronyms of the collections: MZFC, Museo de Zoología “Alfonso L. Herrera”, Facultad de Ciencias, Universidad Nacional Autónoma de México, Mexico City, Mexico. IEXA, Colección Entomológica del Instituto de Ecología A.C., Xalapa, Veracruz, Mexico. CNIN, Colección Nacional de Insectos, Instituto de Biología, Universidad Nacional Autónoma de México, Mexico City, Mexico. UAQE, Colección de Insectos de la Licenciatura en Biología, Facultad de Ciencias Naturales de la Universidad Autónoma de Querétaro, Campus Juriquilla, Querétaro, Mexico. ICZ, Insect Collection of Zacapa, Instituto de Investigaciones del Centro Universitario de Zacapa, Universidad de San Carlos de Guatemala, Zacapa, Guatemala. CMNC, Canadian Museum of Nature Collection, Ottawa, Ontario, Canada. TAMUIC, Texas A&M University Insect Collection, Department of Entomology, Texas A&M University, College Station, Texas, United States of America. CAS, California Academy of Sciences, Department of Entomology, San Francisco, California, United States of America. ASUCOB, Arizona State University Charles O’Brien Collection, Tempe, Arizona, United States of America. CNC, Canadian National Collection of Insects, Arachnids and Nematodes, Agriculture and Agri-Food Canada, Ottawa, Canada. Isodacrys antrum Holotype ♀: [MEXICO: Tamaulipas/El Cielo, 3 km SE Alta Cima/23.03440–99.186967 ± 20 m/860 m, 24.VIII.2009/L.D. Sáenz LSD-370/cloud forest, nest under rock] [WORLD/WEEVIL/DATABASE/WWD0133374] [Isodrusus/or/Isodacrys/det. R.S. Anderson, 20] (CMNC). Paratypes (5 ♀♀): [GUAT.: BAJA VERAPAZ/8.6 km.W. Chilascó, 1,560 m./24.V.1991, R. Anderson/oak|pine|liquidambar/forest litter, 91-18] (2, CMNC). [MEXICO: Chiapas/Mpio: Tapalapa/12 Km N Coapilla/12-III-94, R. Jones] [Isodacrys/Det. R.S. Anderson, 1995] (1, CMNC); [MEX.: CHIAPAS, 3 km./W. San Cristobal, 2,000 m./18.IX.1991, R. Anderson/oak|pine forest, 91-108] (1, MZFC); [MEXICO: Tamaulipas/same data as holotype] [WORLD/WEEVIL/DATABASE/WWD0133381] (1, CMNC). Isodacrys apicale Holotype: ♂, examined; labeled as [151|35/Choix, SIN./13-VIII-35] [HOLOTYPE/Isodacrys/apicale/A.T. Howden/No.7385] [CNC/379712]. MEXICO: Sinaloa, same data as holotype (1 paratype, CMNC); Sonora, 1 mi. W Álamos, 16.VII.1964, H. R. Burke, J. Apperson (1, TAMUIC). Isodacrys brevirostre Holotype: ♀, examined; labeled as [Cuernavaca 6 Mi.,/N., 7500′ Morelos/15-VIII-1954/J. G. Chillcott] [HOLOTYPE/Isodacrys/brevirostre/A.T.Howden/No. 7383] [CNC/379713]. MEXICO: Estado de México, 6 km al SE de Atlautla, 27.IX.2017, Redeo en vegetación, B. Pino-Encino, 2450, 19°00′46″ N, 98°45′26″ W, K. Cortés, L. Delgado (1, IEXA); Estado de México, La Mirasol, 7 km SW Santiago de Tianguistengo, 2.XI.1973, 2, 800 m, C.W. O’Brien (36, ASUCOB; 3, CASENT); Estado de México, Ocoyoacac, 28.X.1973, on misc. flowers, C.W. O’Brien (4, CASENT; 89, ASUCOB); Estado de México, Bejucos, Mex. Temascaltepec, 3.VII.1933, H.E. Hinton, R.L. Usinger (3 paratypes, CASENT); Estado de México, Real de Arriba, VII.1932, H. Hinton (1 paratype, TAMUIC); Estado de México, Hwy 190, 11 mi. W Río Frío, 5.VI.1983, 9800′, C. & L. O’Brien, G. Marshall (3, ASUCOB); Estado de México, Hwy15, 28 mi. W. Toluca, 6.VIII.1982, 9100′, L. O’Brien, G. Wibmer (38, ASUCOB); Guanajuato, 3.6 mi. NE Guanajuato, 5.VII.1985, Woolley, Zolnerowich (1, TAMUIC); Guerrero, 6 km NE Ayotoxtla (Tlatlauquitepec Rd), 24.X.1973, A.N. García A. (2, ASUCOB); Guerrero, 9 km SE Tlatlauquitepec, 24.X.1974, A.N. García A. (1, ASUCOB); Hidalgo, Hwy105 16 mi. N Metzquititlán, 30.VII.1982, 7200′, L. O’Brien, G. Wibmer (23, ASUCOB); Hidalgo, Hwy105 Mineral Real del Monte, 14.VI.1983, 9400′, C. & L. O’Brien, G. Marshall (1, ASUCOB); Hidalgo, Hwy105, 3 mi. S Zacualtipan, 3.VIII.1982, 6959′, C. & L. O’Brien, G. Wibmer (2, ASUCOB); Mexico City, Cañada Contreras, 5.X.1982, s/composit, 2, 550 m, K. Luna (10, MZFC); Mexico City, Road from Mexico City P.N. Cumbres de Ajusco, 4.IX.1982, 2, 750 m, C. & L. O’Brien, G. Wibmer (17, ASUCOB); Mexico City, Delegación Tlalpan, Tlalpuente, 4.XI.1993, L. Torres-Miller (1, CMNC); Mexico City, El Pedregal, 4.IX.1982, 2,340 m, C. & L. O’Brien, G. Wibmer (1, ASUCOB); Mexico City, Unión Perif. Ajusco, 4.IX.1982, 2,340 m, K. Luna (1, MZFC); Mexico City, 7 mi. N Valle de Bravo, 7.VIII.1982, 8300′, C. & L. O’Brien, G. Wibmer (1, ASUCOB); Michoacán, 2 mi. S Carapan, 6.VII.1986, H. & A. Howden (1, CMNC); Michoacán, 2 mi. S Carapan, 6.VII.1985, Woolley, Zolnerowich (1, TAMUIC); Michoacán, 28.5 mi. S Nueva Italia, 9.VII.1985, Woolley, Zolnerowich (1, TAMUIC); Michoacán, 17 mi. W Pátzcuaro, 4.VIII.1982, 7700′, C. & L. O’Brien, G. Wibmer (6, ASUCOB); Morelos, 8 km N Cuernavaca, Hwy 95, 5.IX.1982, C. & L. O’Brien, G. Wibmer (5, ASUCOB); Morelos, Santa María Ahuacatitlán, 6.XI.1980, F. Aguirre (1, CNIN); Morelos, Tetela del Volcán, 23.VIII.1984, V. Butze (1, CNIN); Oaxaca, Hwy 175, 27 km NE Oaxaca, 29.VIII.1982, 8500′, C. & L. O’Brien, G. Wibmer (1, ASUCOB); Oaxaca, Hwy 175, 66 km NE Oaxaca, 29.VIII.1982, 8500′, C. & L. O’Brien, G. Wibmer (1, ASUCOB); Oaxaca, Carr. Oaxaca-Tuxtepex, bosque de pino-encino, en hojarasca, 2,296 m, 17°20′57.8″ N, 96°30′57.5″ W, 9.VI.2018, M. Barrios (6, MZFC); Oaxaca, 2 mi. north San José Pacífico, 16.VII.1974, Clark, Murray, Ashe, Schaffner (4, CMNC); Oaxaca, 2 miles north San José Pacífico, 17.VII.1974, Clark, Murray, Ashe, Schaffner (2, TAMUIC); Oaxaca, 8 km S Suchixtepec, Río Molino, 19.VI.1979, 2,200 m, H. & A. Howden (30, CMNC; 2, ASUCOB); Oaxaca 8 km S Suchixtepec, Río Molino, 6.VIII.1986, H. & A. Howden (1, CMNC); Oaxaca, 10 km S Suchixtepec, 24.VII.1992, Roadside beating, 2,000 m, R.S. Anderson (24, CMNC); Oaxaca, 1 mi. S Suchixtepec Hwy 175, 2.VI.1983, 9200′, C. & L. O’Brien, G. Marshall (2, ASUCOB); Puebla, 67 mi. E Puebla, 26.VI.1971, 7800′, C.W. & L. O’Brien, Marshall (1, CASENT); Puebla, 7 mi. N Zacapoaxtla, 12.VI.1983, 5000′, C. & L. O’Brien, G. Marshall (1, ASUCOB); Puebla, 6 mi. NE Zacatepec, 27.VI.1975, sifting leaf litter, D.S. Chanddler (1, ASUCOB); Puebla, 6 mi. NE Zacatepec, 27.VI.1975, L.E. Watrous (1, ASUCOB); Puebla, Zacatlán km 2 desv. a Piedras Encimadas, 6.VII.2006, 2,449 m, 20°01′07″ N, 98°04′43″ W, L. Cervantes, D. Brzoska (1, CNIN); Veracruz, Hwy 140, 17 mi. NW Jalapa, 20.VIII.1982, 8000′, C. & L. O’Brien, G. Wibmer (1, ASUCOB). Isodacrys buchanani MEXICO: Puebla, 6 mi. SE Tehuacán, 7.VII.1973, Mastro, Schaffner (2, TAMUIC; 1, CMNC); Tamaulipas, 4 mi. S San Fernando, Hwy 101, 5.V.1983, C. & L. O’Brien, G. Marshall (1, ASUCOB). UNITED STATES OF AMERICA: Texas, Duval Co., 1 mi. W San Diego, 10.IV.1973, W.E. Clark (1, TAMUIC); Texas, 15 mi. SW Jct FR 3073 y Hwy 16, 10.IV.1987, B.F. & J.L. Carr (1, CMNC; 2, CNC); Texas, Jim Hogg Co., 5 mi. W Hebbronville, 3.XI.1990, T. Carlow (3, TAMUIC); Texas, 37 mi. N Laredo, Webb Co., 15.X.1970, C.W. O’Brien (53, ASUCOB; 6, CMNC). Isodacrys burkei UNITED STATES OF AMERICA: Texas, Brazos Co., 8.V.1956, H. R. Burke (1 paratype, TAMUIC); Texas, Brazos Co., 12.V.1960, H. R. Burke (1 paratype, TAMUIC); Texas, Brazos Co. Bastrop St. Pk., 24-27.V.1963, M. Kaulbars (2, CMNC); Texas, Brazos Co. Bastrop St. Pk., 5.VI.1989, Sweeping composites, R.S. Anderson (6, CMNC); Texas, Brazos Co., College Station, Lick Creek Park, 12.VI.1993, R. Jones (2, UAQE); Texas, College Station, Brazos Co., 15.IV.1963, H.R. Burke (20, CMNC); Texas, College Station, 3.V.1931, swept from weeds, H.J. Reinhard (1 paratype, TAMUIC); Texas, Brazos Co., on Hwy30 7.6 miles east jct. Hwy. 6, 7.V.1981, Sweeping Coreopsis sp., S. J. Merritt (1, TAMUIC); Texas, Brazos Co., Lick Ck. Pk., ca.3 mi. S College Station, 18.X.1987, R.S. Anderson (4, CASENT; 50, CMNC); Texas, Brazos Co. Lick Creek Park ca. 5 mi. S College Station, 16.IV.1989, Sweeping flowers, R.S. Anderson (2, CMNC); Texas, Lee Co., 29.V.1959, S.D. & R.H. Burke (1 paratype, TAMUIC); Texas, 0.5 mile west of Minter Springs, Brazos Co., 20.IV.1970, V.V. Board (1, TAMUIC); Texas, Leon Co. 9 km N Flynn, 28.V.1995, Quercus incanus, H. & A. Howden (5, CMNC); Texas, Milano, Milam Co., 28.IV.1939, #7315, On tomato plants, very numerous, eating stems particularly, causing plants to fall over (1 paratype, TAMUIC). Isodacrys carlae Holotype ♀: [7000′ nr. Jame,/33 mi. S.E. Saltillo,/Coah. Mex. VII.18′63/A. Howden Acacia] [Isodacrys/n.sp.] (CMNC). Paratypes (45 ♀♀): [7000′ nr. Jame,/33 mi. S.E. Saltillo,/Coah. Mex. VII.18′63/A. Howden Acacia] (1, CMNC); [7500′, nr. Jame,/33 mi.S.E.Saltillo,/Coah. Mex. VII.18′63/A. T. Howden] (6, CMNC; 3, MZFC); [7500′ nr. Jame,/33 mi. S.E. Saltillo,/Coah. Mex. VII.25′63/A. T. Howden] (7, CMNC; 1, UAQE; 1 IEXA); [20 mi. S.E. Saltillo,/6000′, Rt. 015, Coah./Mex., VI.20-21, 1971/H. F. Howden]; [MEXICO: Coahuila/12.4 mi S Saltillo/4-VII-1985, J. Woolley/G. Zolnerowich 85/023] [TAMU-ENTO/X0725914] (1, TAMUIC); [MEXICO: Coahuila/12.4 mi S Saltillo/4-VII-1985, J. Woolley/G. Zolnerowich 85/023] [TAMU-ENTO/X0725888] (1, TAMUIC); [MEXICO, Coah., Hwy./57, 15 mi. SE. Saltillo,/7000′ 12Sept.1982 C.&/L. O’Brien & G. Wibmer] (1, ASUCOB; 1, MZFC); [MEX., Coah., 10 mi./E. Saltillo 7000′/VIII-14-1971 C&L/O’Brien & Marshall] (1, ASUCOB]; [MEXICO, Hidalgo,/Municipio Metztitlán/1 km W. San Cristobal,/20.635°N, 98.846°W,/15-VIII-2017, S.M. Clark] (1, ASUCOB); [MEXICO: Nuevo Leon/road to Galeana, 1/km. N jct. rte. 58/17 July 1988/R. Turnbow] (1, CMNC); [MEXICO: Nuevo Leon/9 mi. west Iturbide/July 3, 1974/Clark, Murray,/Ashe, Schaffner] [TAMU-ENTO/X0725413] (1, TAMUIC); [MEXICO: Nuevo Leon/9 mi. west Iturbide/July 3, 1974/Clark, Murray,/Ashe, Schaffner] [TAMU-ENTO/X0724919] (1, TAMUIC); [MEXICO: Nuevo Leon/9 mi. west Iturbide/July 3, 1974/Clark, Murray,/Ashe, Schaffner] [TAMU-ENTO/X0725718] (1, IEXA); [MEXICO: Nuevo Leon/9 mi. west Iturbide/July 3, 1974/Clark, Murray,/Ashe, Schaffner] [TAMU-ENTO/X0726871] (1, TAMUIC); [MEXICO: Nuevo Leon/9 mi. west Iturbide/July 3, 1974/Clark, Murray,/Ashe, Schaffner] [TAMU-ENTO/X0730294] (1, TAMUIC); [MEXICO: Nuevo Leon/9 mi. west Iturbide/July 3, 1974/Clark, Murray,/Ashe, Schaffner] [TAMU-ENTO/X0726000] (1, UAQE); [MEX. N.L. 40 mi./NW Jcn. Hwy 57&60/VI-22-1971 C.W./O’Brien & Marshall] (1, ASUCOB; 1, MZFC); [MEXICO, N.L., Hwy./58, 32 mi. W. Linares,/6000′ 11Sept.1982 C.&/L. O’Brien & G. Wibmer] (3, ASUCOB); [MEXICO, N.L., 11 km./NE. S. Antonio Peña/Nevada, 2,000 m. Aug16/1977A.N.Garcia A.] [beating pine and/miscellaneous] (1, ASUCOB); [MEX., N.L., 29 mi./SW. Linares 5700′/VIII-15-1971 C&L/O’Brien & Marshall] (1, ASUCOB); [MEXICO: San Luis Potosi/4 km. W Guadalcazar/18 July 1988/R. Turnbow] [Isodacrys/geminatum Howd./det. R.S. Anderson 90] (1, CMNC); [MEXICO: Tamaulipas/19.6 mi. sw. Jaumave/18-VII-1973/Gaumer and Clark] [TAMU-ENTO/X0725801] [Isodacrys/sp./DET./A. Howden] (1, TAMUIC); [MEXICO: Tamaulipas/19.6 mi. sw. Jaumave/18-VII-1973/Gaumer and Clark] [TAMU-ENTO/X0727602] (1, TAMUIC); [MEXICO: Tamaulipas/19.6 mi. sw. Jaumave/18-VII-1973/Gaumer and Clark] [TAMU-ENTO/X0725553] (1, TAMUIC); [MEXICO, Tam., Hwy./101, 28 mi. NE. Tula/4500′ 22July1982 CW&/L. O’Brien & G. Wibmer] (1, ASUCOB). Isodacrys confusum Holotype ♂: [MEXICO: Tamaulipas/16.3 mi. ne. Jaumave/18-VII.1973/Gaumer and Clark] [taken on/Mimosa/monancistra] [TAMU-ENTO/X0729035] (TAMUIC); paratypes (8 ♂♂, 28 ♀♀): [MEXICO, Tam., Hwy 101/18 mi. SW. Cd. Victoria,/4300′ 22July1982 C.W.&/L. O’Brien & G. Wibmer] (1 ♂, 1 ♀, ASUCOB; 1 ♂, MZFC); [MEXICO, Tam., Hwy 101/24 mi. SW. Cd. Victoria,/2150′ 23July1982 C.W.&/L. O’Brien & G. Wibmer] (1 ♂, 2 ♀, ASUCOB); [MEXICO, Tam., Hwy 101/22 mi. SW. Cd. Victoria,/3000′ 22July1982 C.W.&/L. O’Brien & G. Wibmer] (1 ♂, 1 ♀, ASUCOB); [MEXICO: Tamaulipas/same data as holotype] [taken on/same data as holotype] [TAMU-ENTO/X0726618] (1 ♀, TAMUIC); [MEXICO: Tamaulipas/same data as holotype] [taken on/same data as holotype] [TAMU-ENTO/X0727115] (1 ♀, TAMUIC); [MEXICO: Tamaulipas/same data as holotype] [taken on/same data as holotype] [TAMU-ENTO/X0725169] (1 ♀, TAMUIC); [MEXICO: Tamaulipas/same data as holotype] [taken on/same data as holotype] [TAMU-ENTO/X0724605] (1 ♀, TAMUIC); [MEXICO: Tamaulipas/same data as holotype] [taken on/same data as holotype] [TAMU-ENTO/X0725410] (1 ♀, TAMUIC); [MEXICO: Tamaulipas/same data as holotype] [taken on/same data as holotype] [TAMU-ENTO/X0726220] (1 ♀, TAMUIC); [MEXICO: Tamaulipas/same data as holotype] [taken on/same data as holotype] [TAMU-ENTO/X0726502] (1 ♀, TAMUIC); [MEXICO: Tamaulipas/same data as holotype] [taken on/same data as holotype] [TAMU-ENTO/X0727010] (1 ♂, TAMUIC); [MEXICO: Tamaulipas/same data as holotype] [taken on/same data as holotype] [TAMU-ENTO/X0726280] (1 ♀, TAMUIC); [MEXICO: Tamaulipas/same data as holotype] [taken on/same data as holotype] [TAMU-ENTO/X0725437] (1 ♀, TAMUIC); [MEXICO: Tamaulipas/same data as holotype] [taken on/same data as holotype] [TAMU-ENTO/X0725446] (1 ♀, TAMUIC); [MEXICO: Tamaulipas/same data as holotype] [taken on/same data as holotype] [TAMU-ENTO/X0725407] (1 ♀, TAMUIC); [MEXICO: Tamaulipas/same data as holotype] [taken on/same data as holotype] [TAMU-ENTO/X0725141] (1 ♀, TAMUIC); [MEXICO: Tamaulipas/same data as holotype] [taken on/same data as holotype] [TAMU-ENTO/X0725168] (1 ♀, TAMUIC); [MEXICO: Tamaulipas/same data as holotype] [taken on/same data as holotype] [TAMU-ENTO/X0725412] (1 ♀, TAMUIC); [MEXICO: Tamaulipas/same data as holotype] [taken on/same data as holotype] [TAMU-ENTO/X0726973] (1 ♀, IEXA); [MEXICO: Tamaulipas/same data as holotype] [taken on/same data as holotype] [TAMU-ENTO/X0725112] (1 ♀, IEXA); [MEXICO: Tamaulipas/same data as holotype] [taken on/same data as holotype] [TAMU-ENTO/X0725393] (1 ♀, UAQE); [MEXICO: Tamaulipas/same data as holotype] [taken on/same data as holotype] [TAMU-ENTO/X0729284] (1 ♀, UAQE); [MEXICO: Tamaulipas/same data as holotype] [taken on/same data as holotype] [TAMU-ENTO/X0728579] (1 ♂, TAMUIC); [MEXICO: Tamaulipas/same data as holotype] [taken on/same data as holotype] [Isodacrys/sp./DET./A. Howden] [TAMU-ENTO/X0728911] (1 ♀, MZFC); [MEXICO: Tamaulipas/same data as holotype] [taken on/same data as holotype] [TAMU-ENTO/X0728902] [♂] (1 ♂, MZFC); [MEXICO: Tamaulipas/same data as holotype] [TAMU-ENTO/X0726515]; [MEXICO: Tamaulipas/same data as holotype] [taken on/same data as holotype] [Isodacrys/geminatum/Howden/det. R.S. Anderson 1981] (1 ♀, CMNC); [MEXICO: Tamaulipas/same data as holotype] [taken on/same data as holotype] (1 ♂, 3 ♀♀, CMNC). Isodacrys crispum MEXICO: Durango, 25 mi. W Durango, 10.VI.1964, Oak, H. Howden (5, CMNC); Durango, 25 mi. W Durango, 29.VI.1964, H. Howden (30, CMNC; 2 ASUCOB); Durango, 25 mi. W Durango, 3.VII.1964, H. & A. Howden (3, CNC); Durango, 28 mi. W Durango, 9.VI.1967, Quercus clivicola *F. consanguinea*, C.H. Maen (1, CMNC); Durango, 25 mi. W Durango, 18.VIII.1974, on Quercus, 7800′, C.W. & L. O’Brien, Marshall (2, UAQE); Durango, 25 mi. W Durango, 18.VIII.1974, on pine/on Quercus, 7800′, C.W. & L. O’Brien, Marshall (34, ASUCOB); Durango, 26.5 mi. SW Durango, 21.VII.1982, General sweeping, Fred. G. Andrews (9, CMNC); Zacatecas, 27 mi NW Fresnillo, Hwy 45, 17.VIII.1974, on Quercus, 7800′, C.W. & L. O’Brien, Marshall (32, ASUCOB). Isodacrys ellipticum GUATEMALA: Zacapa, San Lorenzo Quarry Road, 3-7 km N Sta. Cruz, CA9, 17.VII.2008, Dry forest, C. & L. O’Brien, F. Skillman (1, ASUCOB). HONDURAS: Comayagua, Comayagua, 2.VI.1978, Gary V. Manley (1, CMNC); Cortés, 5 km N Cofradía, 5.VIII.1977, C. & L. O’Brien, G. Marshall” (41, ASUCOB); Francisco Morazán, Distrito Central, 5 mi. S Tegucigalpa, 9.VI.1974, night, 4000′, C. & L. O’Brien, G. Marshall (3, CASENT); Francisco Morazán, 17 mi. NE Talanga, 16.VI.1974, 2900′, C. & L. O’Brien, G. Marshall (129, ASUCOB; 3, CASENT); Francisco Morazán, 23.8 km SW Talanga, 3.VI.1993, R. Turnbow (1, CMNC); Francisco Morazán, Tamara valley, 5.X.1993, R. Turnbow (1, CMNC). Isodacrys fasciatum Holotype ♀: [MEX., Coah., 10 mi./E. Saltillo 7000′/VIII-14-1971 C&L/O’Brien & Marshall] (ASUCOB). Paratypes (11 ♀♀): [MEX., Coah.,10 mi./same data as holotype] (2, ASUCOB); [MEXICO: Coahuila/12.4 mi S Saltillo/4-VII-1985, J. Woolley/G. Zolnerowich 83/023] [TAMU-ENTO/X0727876] (1, TAMUIC); [MEXICO, Dgo., 22 mi./NE. Durango, 6200′/Aug. 20, 1974 C.W.&L./O’Brien & Marshall] (7, ASUCOB); [MEX. N.L. 10 mi./S. Jcn. 57 & 60/VI-23-1971 L&CW/O’Brien & Marshall] (1, ASUCOB). Isodacrys frontalis Holotype ♀: [MEX.: OAX.; 20 mi S/Juchatengo, 6000′/29.V.1971, S. Peck/Ber207, oak litter] (CMNC). Paratypes (23 ♀): [GUATEMALA: Sacatepéquez/5 km SE Antigua/14.53495–90.69367 ± 50 m/2,150 m, 10.VI.2009/LLAMA Wa-B-08-1-49] [WORLD/WEEVIL/DATABASE/WWD0041418] (1 CMNC); [GUATEMALA: Sacatepéquez/5 km SE Antigua/14.53495–90.69367 ± 50 m/2,150 m, 10.VI.2009/LLAMA Wa-B-08-1-49] [WORLD/WEEVIL/DATABASE/WWD0041419] (1, CMNC); [GUATEMALA: Sacatepéquez/5 km SE Antigua/14.53495–90.69367 ± 50 m/2,150 m, 10.VI.2009/LLAMA Wa-B-08-1-49] [WORLD/WEEVIL/DATABASE/WWD0041420] (1, CMNC); [GUATEMALA: Sacatepéquez/5 km SE Antigua/14.53510–90.69384 ± 50 m/2,150 m, 10.VI.2009/LLAMA Wa-B-08-1-44] [WORLD/WEEVIL/DATABASE/WWD0041311] [Isodacrys/GUA 1/det. R.S. Anderson, 20] (1, CMNC); [GUATEMALA: Sacatepéquez/5 km SE Antigua/14.53529–90.69404 ± 50 m/2,150 m, 10.VI.2009/LLAMA Wa-B-08-1-38] [WORLD/WEEVIL/DATABASE/WWD0041144] (1, CMNC); [GUATEMALA: Sacatepéquez/5 km SE Antigua/14.53504–90.69377 ± 50 m/2,150 m, 10.VI.2009/LLAMA Wa-B-08-1-46] [WORLD/WEEVIL/DATABASE/WWD0041358] (1, CMNC); [GUATEMALA: Sacatepéquez/5 km SE Antigua/14.53504–90.69377 ± 50 m/2,150 m, 10.VI.2009/LLAMA Wa-B-08-1-46] [WORLD/WEEVIL/DATABASE/WWD0041359] (1, CMNC); [GUATEMALA: Sacatepéquez/5 km SE Antigua/14.53504–90.69377 ± 50 m/2,150 m, 10.VI.2009/LLAMA Wa-B-08-1-46] [WORLD/WEEVIL/DATABASE/WWD0041360] (1, CMNC); [GUATEMALA: Sacatepéquez/5 km SE Antigua/14.53504–90.69377 ± 50 m/2,150 m, 10.VI.2009/LLAMA Wa-B-08-1-46] [WORLD/WEEVIL/DATABASE/WWD0041361] (1, CMNC); [GUATEMALA: Sacatepéquez/5 km SE Antigua/14.53504–90.69377 ± 50 m/2,150 m, 10.VI.2009/LLAMA Wa-B-08-1-46] [WORLD/WEEVIL/DATABASE/WWD0041363] (1, CMNC); [GUATEMALA: Sacatepéquez/5 km SE Antigua/14.53507–90.69380 ± 50 m/2,150 m, 10.VI.2009/LLAMA Wa-B-08-1-45] [WORLD/WEEVIL/DATABASE/WWD0041326] (1, CMNC); [GUATEMALA: SACATEPEQUEZ/Guatemala City, Cerro/Alux, 2,260 m., 11.VI.1991/R. Anderson, wet oak/forest, 91-60] (1, CMNC); [GUATEMALA, 9 mi.W./Cd. Guatemala 6900′/VI-7-1974C.W. &L./O’Brien &Marshall] (2, ASUCOB). [MEX.: OAX, same data as holotype] (4, CMNC); [MEXICO: Oaxaca 3.2 km./S. San Jose del Pacifico/2,440 m, 22.VII.1992, J.S./Ashe,oak-pine for. litt.] (1, CMNC); [MEXICO: Oaxaca, 5.1 km./S. Suchixtepec, 2,150 m/24.VII.1992,92-024,R.S./Anderson, oak|alder|pine/for., leaf litter Berlese] (3, CMNC); [MEXICO: Oaxaca, 23 km./S.W. Valle Nacional, KM76/1,300 m, 26.VII.1992,92-029/R.S. Anderson, cloud forest/leaf litter Berlese] (1, CMNC). Isodacrys geminatum MEXICO: Mexico City, Mpio. Xilotepec, Hwy57, 25.VII.2006, oak forest litter, 2,700 m, 20°04′.797′ N, 99°37.906′ W, R.S. Anderson (1, CMNC); Oaxaca, 22 km NE Oaxaca, 18.VI.1979, 2,600 m, H. & A. Howden (1, CMNC); Oaxaca, 3 mi. N Suchixtepec, 4.VI.1971, Ber209, oaklitter, 9500′, S. Peck (1, CMNC); Puebla, 6 mi. NE Zacatepec, 27.VI.1975, L.E. Watrous (9, ASUCOB); Querétaro, Mpio. Colón, Cerro Zamorano, 26.VII.2006, oyamel/oak forest litter, 3,100 m, 20°55.967′ N, 100°11.021′ W, R.S. Anderson (1, CMNC); Querétaro, Mpio. Huimilpan, 1 km NW La Beata, 2.XII.2003, sample #6, leaf litter, 2,500 m, 20° 28.42′ N, 100° 14.49′ W, R. Jones (1, UAQE); Tlaxcala, 6.8 km N Taxco, Hwy 119, 10.VII.1992, ex. misc. Mushrooms, 2,820 m, J.S. Ashe #36 (1, CMNC). Isodacrys guatemalenum GUATEMALA: Sacatepéquez, Antigua, 16.VIII.1947, 5000′, C. & P. Vaurie” (1, CMNC); Sacatepéquez, Santa Lucía Milpas Altas, 24.VI.1993, 1,800 m, B.D. Gill (1, CMNC). HONDURAS: Choluteca, 1 km W San Marcos de Colón, 23.VII.1977, C. & L. O’Brien, G. Marshall (12, ASUCOB); Comayagua, Comayagua, 18.VII.1977, J.V. Mankins (2, CMNC); Comayagua, 22 km N Comayagua, 18.VII.1977, C. & L. O’Brien, G. Marshall (80, ASUCOB; 12, CASENT). Isodacrys kuchii Holotype ♂: [MEXICO: Puebla/Mpio: Zapotitlán Salinas/1 km N San Juan Raya] [18° 19′ 11″, 97° 36′ 50″/19-vii-2011, A. Obregón/1,755 m, Matorral] [Isodacrys/geminatum/Howden 1961/Det. Jesús Luna Cozar 2015] (UAQE). Paratypes (1 ♂, 1 ♀): [MEXICO: Puebla/same data as holotype] [18° 19′ 11″, 97° 36′ 50″/same data as holotype] [Isodacrys/same data as holotype] (1 ♂, UAQE); [MEXICO: Puebla/6 Mi. SW. Tehacan/July 8, 1973/Schaffner & Mastro] [TAMU-ENTO/X0725504] [Isodacrys/?geminatum/DET./A. Howden] (1 ♀, TAMUIC). Isodacrys mexicanum MEXICO: Aguascalientes, 24 miles West of Aguascalientes, 5.VI.1956, B. Banta (1, CASENT); Mexico City, Milpa Alta, 26.VII.1980, H. Brailovsky (1, CNIN); Mexico City, Pedregal de San Ángel, Loc. 21, 22.VIII.1961 (2, CNIN); Mexico City, Zumpango, 18.VII.1981, Peter Jump (1, CMNC; 1, TAMUIC); Michoacán, km 154 car. Irapuato-Guadalajara, 26.VII.1984, M. García” (1, CNIN); Morelos, Tepoztlán, 11.X.1968, R. Muñíz V. (1, CMNC; 1, ASUCOB); Morelos, YMCA camp. Tepoztlán, 21.VIII.1958, H.F. Howden (4, CMNC); Oaxaca, 1.1 mi. W El Tule, 87/048, 5400′, Woolley, Zolnerowich (2, TAMUIC). Isodacrys obrienorum Holotype ♂: [GUATEMALA, 12 mi.SE./San Cristobal de To-/tonicapan, 9800′/VII-28.1974] [under stones] [C.W. & L.B. O’Brien/& G.B. Marshall] (ASUCOB). Paratypes (6 ♂♂, 6 ♀♀): [GUATELAMA: Jalapa/4 km E Mataquescuintla/14.52705–90.14671 ± 55 m/2,660 m, 2.VI.2009, /LLAMA #Go-B-07-1-04/cloud forest beating vegetation] [WORLD/WEEVIL/DATABASE/WWD0125181] (1 ♀, CMNC); [GUATELAMA: Jalapa/4 km E Mataquescuintla/14.52705–90.14671 ± 55 m/2,660 m, 2.VI.2009,/LLAMA #Go-B-07-1-04/cloud forest beating vegetation] [WORLD/WEEVIL/DATABASE/WWD0125188] (1 ♂, CMNC); [GUATELAMA: Jalapa/4 km E Mataquescuintla/14.52705–90.14671 ± 55 m/2,660 m, 2.VI.2009,/LLAMA #Go-B-07-1-04/cloud forest beating vegetation] [WORLD/WEEVIL/DATABASE/WWD0125177] (1 ♂, CMNC); [GUATEMALA, 12 mi.SE./San Cristobal deTo-/tonicapan, same data as holotype] (3 ♂♂, 4 ♀♀, ASUCOB); [GUATEMALA, 10 mi.NW./SanCristobal de/Totonicapan, 9300′] [VII-29-1974C.W.&L./O’Brien & Marshall] [under stones/and dead/grass clumps] (1 ♀, ASUCOB); [GUATEMALA: San Marcos/above Sibinal/3,009 m, 15.14366–92.06662/8 Jun 2015, R. Anderson/alder litter, 2015-127] (1 ♂, CMNC). Isodacrys okuiltontli Holotype ♂: [MEXICO, Oax., Hwy131/5 mi. N. Telixtlahuaca/6200′ 31Aug.1982 CW. &/L. O’Brien & G. Wibmer] (ASUCOB). Paratypes (5 ♀♀): [MEXICO, Oax, 19.5/km. NW. Huitzo, Hwy./190, 2,260 m, 9.VII./1992 C.L. Bellamy] (1 ♀, ASUCOB); [MEXICO, Oax., Hwy131/same data as holotype] (3 ♀♀, ASUCOB); [MEXICO, Oax., Hwy131/same data as holotype] [Isodacrys/minutus/Sharp/det. C. W. O’Brien, 1999] (1 ♀, ASUCOB). Isodacrys orizabae MEXICO: Oaxaca, 1.8 km S El Tule, 16.VII.1987, dry Acacia scrub, 1,524 m, R.S. Anderson (1, CMNC); Oaxaca, 20 km E Mitla, 3.VIII.1986, 1,800 m, H. & A. Howden (2, CMNC); Oaxaca, Hwy 175, 10 km NE Oaxaca, 16.VI.1979, 1,800 m, H. & A. Howden (1, CMNC); Oaxaca, 5 mi. SW Oaxaca, 4.VIII.1983, Acacia thorn scrub, 4700′, R.S. Anderson, W. Maddison (9, CMNC); Oaxaca, 13 mi. SE Tlalcolula, 2.VI.1974, 6100′ C. & L. O’Brien, G. Marshall (4, ASUCOB); Puebla, 4.4 mi. SW Acatepec, 26.VII.1974, Clark, Murray, Ashe, Schaffner (5, CMNC; 6, TAMUIC); Puebla, 6 km S Acatepec, 19.VIII.1982, Clark, Cave (1, ASUCOB); Veracruz, VIII.1942, Koebele (2, CMNC); Veracruz, Hwy 150, 31 km W Orizaba, 15.VIII.1975, beating Pine, 1,880 m, B. García G. (1, ASUCOB). Isodacrys ovipennis MEXICO: Tamaulipas, Hwy101 11 mi. SW Cd. Victoria, 22.VII.1982, 4000′, C. & L. O’Brien, G. Wibmer (37, ASUCOB); Tamaulipas, 2 mi. NE San Fernando, Hwy 101, 31.VII.1970, C.W. O’Brien (1, ASUCOB); Tamaulipas, 4 mi. S San Fernando, Hwy 101, 5.V.1983, C. & L. O’Brien, G. Marshall (2, ASUCOB). UNITED STATES OF AMERICA: Texas, Cameron Co. Laguna Atascosa Nat. Wild. Ref. rd. to Laguna Madre, 20.X.1989, sweeping *Palafoxia texana* var. ambigua, R.S. Anderson, E. Riley (13, CMNC); Texas, Cameron Co. Laguna Atascosa Wdlf. Ref, 20.X.1989, E.G. Riley (5, ASUCOB); Texas, Encino, 13.III.1966, *Modiola caroliniana*, M.F. Schuster (1, CMNC); Texas, Kenedy Co. Los Olmos Creek at #77, 2 mi. S Riviera, 20.X.1989, sweeping *Palafoxia texana* var. ambigua, R.S. Anderson, E. Riley (13, CMNC; 1, CASENT); Texas, Kenedy Co. 10 mi. N Norias, 10.X.1989, D.J. Heffern (1, CMNC); Texas, Edinburg, 3.VI.1962, W.L. Sterling (1, TAMUIC); Texas, Kingsville, Kleberg Co., 25.VII.1951, H.R. Burke (1, TAMUIC); Texas, 10 miles NW La Gloria, 6.XI.1989, W.F. Chamberlain (1, TAMUIC); Texas, Kleberg Co., Jct. Los Olmos Creek & Hwy 77, 19.X.1990, T. Carlow, E. Riley (2, TAMUIC); Texas, Palmitto Hill, 10 mi. W Boca Chica, 13.X.1985, R. Turnbow (1, CMNC). Isodillex minutum MEXICO: MICHOACÁN, 2 mi. S Carapan, 6.VII.1985, Woolley, Zolnerowich” (5 specimens, TAMUIC; 3 specimens, CMNC), “MEXICO: MICHOACÁN, 18.5 km E Morelia, 28.VII.1988, Pine/oak/Acacia, 2,220 m, R. S. Anderson” (1 specimen, CMNC), “MEXICO: MICHOACÁN, 17 mi. W Pátzcuaro, 14.VIII.1982, 7700′, C. & L. O’Brien, G. Wibmer” (1 specimen, ASUCOB). Isodillex plumosum Holotype (♂): [61 mi. W. Fresnillo/Zacatecas, MEX/8000 ft. VI-25-54/R. H. Brewer] [III#3] [Isodacrys/n.sp. #2/AH ‘62] [CASENT/8424336]. Paratypes (3 ♂♂ and 11 ♀♀): [61 mi. W. Fresnillo/same data as holotype] [III#3] [Cal Acad.] [CASENT/8424322] (1 ♂ CASENT); [61 mi. W. Fresnillo/same data as holotype] [III#3] [CASENT/8424323] (1 ♂ CASENT); [61 mi. W. Fresnillo/same data as holotype] [III#3] [CASENT/8424331] (1 ♂ CASENT); [61 mi. W. Fresnillo/same data as holotype] [III#3] [Cal Acad.] [Isodacrys/n.sp.#2] [CASENT/8424321] (1 ♀ CASENT); [61 mi. W. Fresnillo/same data as holotype] [III#3] [CASENT/8424324] (1 ♀ CASENT); [61 mi. W. Fresnillo/same data as holotype] [III#3] [CASENT/8424325] (1 ♀ CASENT); [61 mi. W. Fresnillo/same data as holotype] [III#3] [CASENT/8424326] (1 ♀ CASENT); [61 mi. W. Fresnillo/same data as holotype] [III#3] [CASENT/8424327] (1 ♀ CASENT); [61 mi. W. Fresnillo/same data as holotype] [III#3] [CASENT/8424328] (1 ♀ CASENT); [61 mi. W. Fresnillo/same data as holotype] [III#3] [CASENT/8424329] (1 ♀ CASENT); [61 mi. W. Fresnillo/same data as holotype] [III#3] [CASENT/8424330] (1 ♀ CASENT); [61 mi. W. Fresnillo/same data as holotype] [III#3] [CASENT/8424332] (1 ♀ CASENT); [61 mi. W. Fresnillo/same data as holotype] [III#3] [CASENT/8424333] (1 ♀ CASENT); [61 mi. W. Fresnillo/same data as holotype] [III#3] [CASENT/8424334] (1 ♀ CASENT); [61 mi. W. Fresnillo/same data as holotype] [III#3] [CASENT/8424335] (1 ♀ CASENT).

### New Species Registration

The following information was supplied regarding the registration of a newly described species: Publication LSID: urn:lsid:zoobank.org:pub:F5D5C6F7-3FB4-4D1F-91F3-0D6380C8FF59 Isodacrys antrum LSID: urn:lsid:zoobank.org:act:FBE7F34A-9C93-4BDC-9423-3D3A915B926A. Isodacrys carlae LSID: urn:lsid:zoobank.org:act:146A1489-52AE-477D-A304-86136B5880BA. Isodacrys confusum LSID: urn:lsid:zoobank.org:act:3EDF5293-38FD-4217-8730-164074806BAA. Isodacrys fasciatum LSID: urn:lsid:zoobank.org:act:881D4538-9CB8-49E5-9641-CCF3973623E1. Isodacrys frontalis LSID: urn:lsid:zoobank.org:act:DB24A34B-7EF1-44AD-8DFD-5823FC083899. Isodacrys kuchii LSID: urn:lsid:zoobank.org:act:CB805B2D-80BA-4B36-9B6D-90740B222917. Isodacrys obrienorum LSID: urn:lsid:zoobank.org:act:9DB9D7AA-F4AB-4424-A810-3DD5BF6ABA56. Isodacrys okuiltontli LSID: urn:lsid:zoobank.org:act:01FC3BDC-D6AC-48D1-BF6A-60CF65BB6626. Isodillex LSID: urn:lsid:zoobank.org:act:2AD213BD-E6DE-4146-A578-426D26C2002E. Isodillex plumosum LSID: urn:lsid:zoobank.org:act:955038E3-B522-4A22-9149-896ED0092C6B.
